# Co-Calibrating Physical and Psychological Outcomes and Consumer Wearable Activity Outcomes in Older Adults: An Evaluation of the coQoL Method

**DOI:** 10.3390/jpm10040203

**Published:** 2020-10-31

**Authors:** Vlad Manea, Katarzyna Wac

**Affiliations:** 1 Quality of Life Technologies Lab, University of Copenhagen, Sigurdsgade 41, 2200 Copenhagen, Denmark; katarzyna.wac@unige.ch or; 2 Quality of Life Technologies Lab, University of Geneva, Route de Drize 7, 1227 Carouge, Switzerland

**Keywords:** ambulatory assessment, physical activity, social support, anxiety, depression, nutrition, memory, sleep, health-related quality of life, wearable

## Abstract

Inactivity, lack of sleep, and poor nutrition predispose individuals to health risks. Patient-Reported Outcomes (PROs) assess physical behaviours and psychological states but are subject of self-reporting biases. Conversely, wearables are an increasingly accurate source of behavioural Technology-Reported Outcomes (TechROs). However, the extent to which PROs and TechROs provide convergent information is unknown. We propose the coQoL PRO-TechRO co-calibration method and report its feasibility, reliability, and human factors influencing data quality. Thirty-nine seniors provided 7.4 ± 4.4 PROs for physical activity (IPAQ), social support (MSPSS), anxiety/depression (GADS), nutrition (PREDIMED, SelfMNA), memory (MFE), sleep (PSQI), Quality of Life (EQ-5D-3L), and 295 ± 238 days of TechROs (Fitbit Charge 2) along two years. We co-calibrated PROs and TechROs by Spearman rank and reported human factors guiding coQoL use. We report high PRO—TechRO correlations ($r_{S}$ ≥ 0.8) for physical activity (moderate domestic activity—light+fair active duration), social support (family help—fair activity), anxiety/depression (numeric score—sleep duration), or sleep (duration to sleep—sleep duration) at various durations (7–120 days). coQoL feasibly co-calibrates constructs within physical behaviours and psychological states in seniors. Our results can inform designs of longitudinal observations and, whenever appropriate, personalized behavioural interventions.

## 1. Introduction

Chronic diseases represent a significant share of the burden of disease globally [1]. They are responsible for 86% of all deaths [2]. In Europe, chronic diseases affect over 80% of adults over 65 and incur 70% of the increasing healthcare costs [3]. The most common chronic diseases are cardiovascular, pancreatic, pulmonary, and neoplastic. Unhealthy lifestyle and behaviours, such as physical inactivity, insufficient sleep, poor nutrition, and tobacco intake, explain up to 50% of the risk of chronic disease [4]. We expect the importance of the long-term risk of disease to increase as the world population is ageing [5]. As age dramatically contributes to the risk of multiple diseases [1], the healthy old is a population both inherently at risk and appropriate for primary disease prevention.

Currently, human health studies assess behaviours through a combination of self-reported outcomes [6], in particular patient-reported outcomes (*PRO*, [6]), and, more recently, patient-generated technology-reported outcomes (*TechRO*, [6]). Patient-reported outcomes include questionnaires with validated scales that assess individual outcomes momentarily or for a given recall period (e.g., *“During the past month, how often have you had trouble sleeping?”*). However, self-reports are known to be the subject of biases related to the inherent shortcomings of participant reporting. The questionnaires are inconvenient, infrequent, memory-biased, socially conditioned, and qualitative. For example, seniors reporting physical activity tend to overestimate the amount undertaken [7], while subjective sleep is less reliable than objective sleep according to studies of sleep, ageing, and cognition [8,9].

In an attempt to address the shortcomings of self-reports and based on technological advances, we propose the *coQoL* PRO-TechRO co-calibration method. Our research primarily focuses on assessing behaviours and outcomes by combining questionnaires with devices such as smartphones and wearables, assessing multiple outcomes (e.g., physical activity, sleep, and heart rate) *momentarily*, and, if collected for a long time, also *longitudinally* [10]. Numerous studies used validated, expensive, and bulky lab-grade devices (e.g., ActiGraph), although for a limited time due to the user burden and discomfort of wearing them [11]. Conversely, consumer-friendly wearables measure continuously and objectively TechROs, increasingly more accurately, as technology progresses [12]. Also, more individuals opt for consumer-friendly wearable devices; the market size for consumer wearables will likely double by 2022 [13]. More recent research showed that consumer wearables could assess multiple behaviours accurately [14], unobtrusively [15], and continuously [16] while worn by participants during the natural unfolding of their daily lives. Overall, consumer devices are accurate and used enough to be leveraged in human health studies.

There exist prior work aiming at co-calibration of physical and psychological outcomes with technology-related ones, as discussed in this paper. We identify the previous work by following by following a semi-structured literature review detailed in Appendix A.1. Table 1 presents the PRO-TechRO co-calibration studies resulting from our literature review for the following outcomes: physical activity, social support, anxiety and depression, memory, sleep, and health-related Quality of Life. For each study, the table presents the PROs and TechROs used for co-calibration, the study design, the analysis methodology, and a summary of results. As for the PRO, the table presents the long names of the PRO instruments leveraged in the study, followed by the TechRO details, at least including the name and its form factor (consumer wearable or research-grade accelerometer, and position on the body). The study design details include its target population, sample size and age, and study duration. Past co-calibration methods range from simple descriptive statistics to inferential statistics via correlation methods, to machine learning, including regression and classification. The results bring a summary of PRO-TechRO co-calibration efforts, as presented in the paper.

To better emphasize the difference between state of the art and our work, we recall that we focus on healthy seniors and our method implies repeated sets of different PRO assessments in longitudinal daily life TechRO assessment settings, based on consumer wearables. All studies presented in Table 1 have at least one feature (marked in violet) that excludes them from co-calibrating PRO questionnaires with TechRO consumer wearables in healthy seniors *in the wild* over long periods (above the typical 7–14 days found in the literature).

Table 1 does not include studies on nutrition, since, to our best knowledge, the co-calibration of the *diet* with distant measures such as *steps* or *sleep* using questionnaire PROs and consumer wearables (or, at the very least, accelerometers) does not exist in the literature. However, there are numerous articles on energy expenditure estimates measured by consumer wearables that guide the energy intake (food types and qualities) for individuals following dietary recommendations [17,18,19].

As can be seen from Table 1, most studies focus on specific PROs suitable for the study aim; some of the PROs are disease-specific, which also relate to the user groups in the study (e.g., students, patients with a given condition). As for the TechROs, we observe few research-grade wearables, and many consumer-grade ones (Fitbit); mostly worn as wearable bracelets. The study design is characterized by diverse sample sizes (20–70, with very few examples of 500+ participants) and usually very short duration (7 days or less, very few beyond three weeks). We can call these co-calibration efforts momentary, as valid in these specific periods, for which the data was collected. The co-calibration method themselves used usually leverage descriptive statistical methods and correlations. The results of these co-calibrations rarely report values ≥0.5. In summary, little research focused on assessing the relationships between sets of different outcomes assessed via PROs and consumer wearable TechROs in healthy seniors, in the wild, for extended periods (beyond the typical study duration of 7–14 days).

Our paper is the result of research conducted as part of the EU AAL Caregiver and ME (*CoME*, No. 14-7, 2017–2020) research project and software application. CoME aimed at self-management of health for individuals of old age at risk of mild cognitive impairments and their informal caregivers [20]. The project used numerous PROs to obtain a holistic view of the participants’ health and wellbeing, by covering constructs that are both reflective (physical activity, anxiety, depression, memory, sleep) and formative (nutrition and social support) for the individual’s Quality of Life (*QoL*) [21]. These constructs assess participants’ health state and correspond to behavioural risk factors of dementia, as guided by the goals of the project [22,23,24,25].

Our study involved 42 seniors from Hungary and Spain. The seniors provided PROs on questionnaires chosen by the consortium of the CoME project partners along [22]. The measured outcomes included physical activity (using the International Physical Activity Questionnaire Long, or *IPAQ* [26]), social support (Multidimensional Scale of Social Support, *MSPSS* [27]), anxiety and depression (Goldberg Anxiety and Depression Scale, *GADS* [28]), nutrition (Prevention with Mediterranean Diet, *PREDIMED* [29,30] and Self-Reported Mini Nutritional Assessment, *SelfMNA* [31]), memory (Memory Failures of Everyday, *MFE* [32]), sleep (Pittsburgh Sleep Quality Index, *PSQI* [33]), and health-related Quality of Life (EuroQoL with five dimensions and three levels, *EQ-5D-3L* [34]) (Appendix B.1.1 describes the questionnaires and their validated scales in depth). Participants also provided TechROs of physical activity, sleep, and heart rate (Fitbit Charge 2 consumer wearable, [35]) during the study, for up to two years.

Our paper has three objectives. First, we aim at demonstrating the feasibility of our co-calibration method, *coQoL*, by quantifying relationships between PROs and TechROs for our sample. Second, we aim at assessing the quality of the data collected while daily life unfolded for our participants. Third, we aim at informing the design of observational (and potentially interventional) personalized behavioural studies by leveraging the results from the first two objectives.

Our paper is structured as follows. Section 1 provides an introduction. Section 2 describes our materials and methods. Section 3 foregrounds our results. Section 4 discusses our findings. Section 5 concludes the paper.

## 2. Materials and Methods

In this section, we describe the coQoL method applied within our study context (Section 2.1), participants (Section 2.2), protocol (Section 2.3), measured outcomes (Section 2.4), and data analysis (Section 2.5).

### 2.1. Study Context

We conducted this research as part of the EU AAL Caregiver and ME (CoME, No. 14-7), a research project and software application (2017–2020) aimed at self-management of health for individuals of old age at risk of mild cognitive impairments and their informal caregivers [20]. The goals of the CoME project were (1) to relieve the caregiver pressure through monitoring of physical, intellectual, emotional, and social wellbeing of the persons in need of care and (2) to increase seniors’ wellbeing and autonomy in their environment and lower the risk of dementia [62] and healthcare costs in the long term. We achieved the goals by monitoring the seniors’ state, behaviours (including physical activity and sleep), and other factors that influence the risk of dementia [22]. The study was purely observational; it did not include any behaviour intervention elements.

### 2.2. Study Participants

Individuals of older age, owning a smartphone or willing to use a smartphone provided to them, were invited to the care centre in their city (Spain and Hungary) to participate in the study. Forty-two individuals (mean age 69.8 ± 7.4) agreed to join CoME from January 2017 to December 2019.

### 2.3. Study Protocol

All individuals were informed about the study goals and gave their written informed consent for inclusion before the start of the study. We conducted the study under the Declaration of Helsinki. The institutional review board at the University of Geneva (Switzerland) approved the protocol (CoME, No. 14-7) on April 28, 2016. The study protocol pseudonymized all participant identities.

Upon the first visit at the care centre, the participants attended an informational workshop about the project aims. They received Fitbit Charge 2 wearable devices as their own (for the study duration and beyond). Furthermore, they filled a profile questionnaire and registered personal accounts in the CoME software application. Then they associated the Fitbit wearables to their accounts.

In the first and subsequent visits spread through a few months to a year from January 2017 to December 2019, the participants answered several questionnaires (PROs). Whenever needed, they were assisted by caregivers through this process. However, the participants were not explicitly informed about when they will have filled which of the questionnaires to avoid any activity pattern change before the visit.

### 2.4. Measured Outcomes

**Figure 1:**
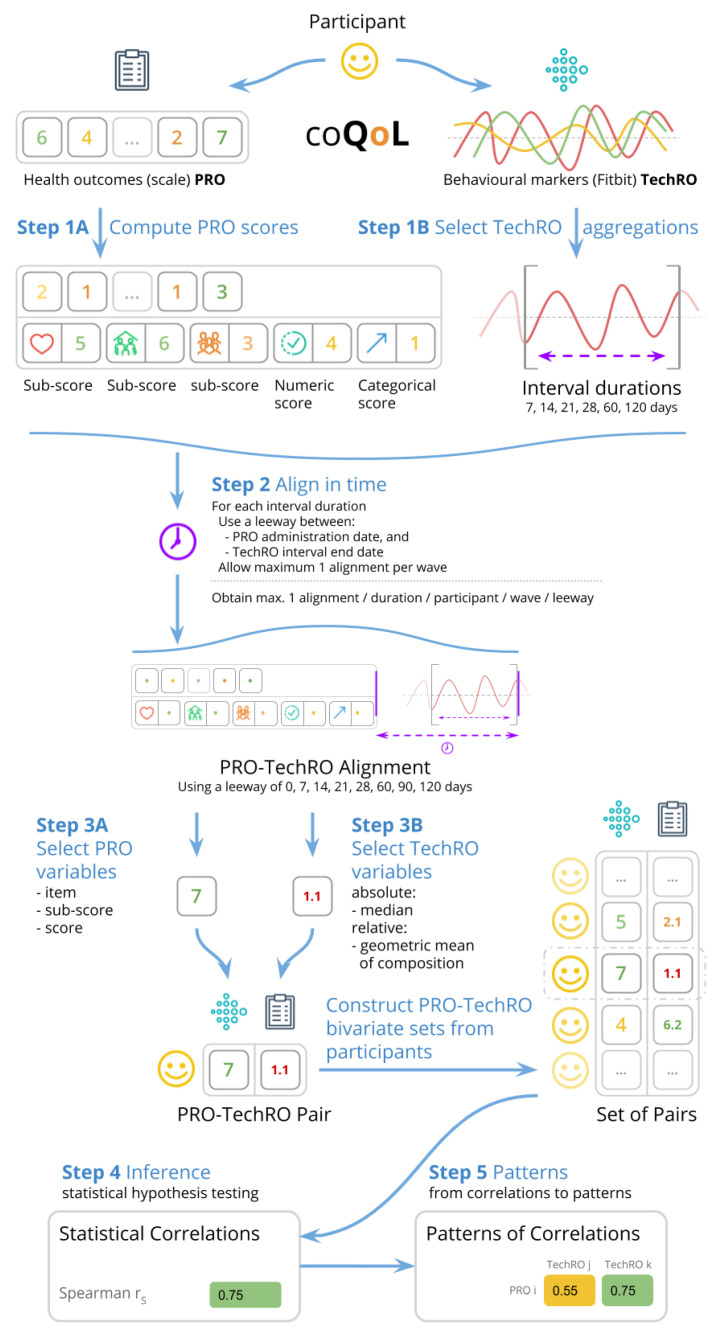
coQoL: a method for PRO and TechRO co-calibration (example for MSPSS PRO).

The study collected PROs from questionnaires with validated scales and TechROs from Fitbit Charge 2 consumer wearables. The PROs and TechROs were then co-calibrated by using the coQoL method illustrated in Figure 1.

#### 2.4.1. Patient-Reported Outcomes (Profile)

At the first visit, in the profile, participants provided their age, gender, ethnicity, profession, education, cohabitants status, height, weight, blood pressure, cholesterol, smoking, alcohol, medication (hypertension), history of personal health issues (diabetes, apnea, insomnia, hyperglycemia, stroke, infarct, depression), and history of family health issues (hypertension, diabetes, stroke, heart attack, dementia).

We included in the analysis participants who self-reported mild disease. We selected participants into three *health groups*: (1) all participants (denoted as the *all* health group), (2) only the healthy participants (*healthy*), and (3) only those with mild disease (*diseased*).

#### 2.4.2. Patient-Reported Outcomes (PROs)

During several study visits, the participants provided answers to questionnaires for eight PROs: physical activity (IPAQ), social support (MSPSS), anxiety and depression (GADS), nutritional adherence to the Mediterranean diet (PREDIMED), nutrition (SelfMNA), memory (MFE), sleep (PSQI), and health-related QoL (EQ-5D-3L). Appendix B.1.1 describes the questionnaires in depth.

We administered the questionnaires in the languages of the respondents (Spanish or Hungarian). Appendix B.1.2 elaborates on the administration of the questionnaires.

The days of administration resulted in distinct periods of answers separated by a few months to one year. We denote these periods as *waves* of participation.

We coded the answers and computed the *scores* (and *sub-scores*, where available) according to the validated scale of each questionnaire. This procedure is depicted as Step 1A in Figure 1. Appendix B.1.3 provides details on the scoring.

We derived for the analysis the following PRO-based *variables*: (1) the individual questions in the questionnaire (denoted *items*), the *sub-scores* (where available), and the *scores* (where available). Most scales have a *numeric score* and a *categorical score*. Most sub-scores are numeric.

This procedure corresponds to Step 3A in Figure 1. All variables can be seen in Table 2. Appendix B.1.4 details the variable derivation for PROs.

#### 2.4.3. Technology-Reported Outcomes (TechROs)

We collected the behavioural wearable markers from the daily aggregates provided by the Fitbit daily activity summary application programmable interface (API) [63]. Appendix B.2.1 motivates our choice for Fitbit as a personal wearable activity monitor in the context of our study.

We processed the wearable data by aggregating it over consecutive days in *aggregate intervals* spanning from 7 to 120 days. We included in the analysis only days with at least 21 hours of Fitbit measurement as *valid days*. Then we required each aggregate interval to have at least 70% valid days. This procedure corresponds to Step 1B in Figure 1. Appendix B.2.2 details the data processing.

The Fitbit consumer wearables provided TechROs as *raw* (energy expenditure, steps, heart rate) and *processed* according to Fitbit’s internal activity recognition algorithms (sedentary duration, durations of physical activity at the light, fair, and vigorous intensities, and sleep) [35].

We derived TechRO-based *variables* in two *amounts*. The *absolute* amount refers to the TechROs enumerated above. For this amount, we computed for each interval the median of daily measurements.

We derived the *relative* amount variables from the total daily durations of physical activity (and, separately, physical activity and sleep for all 24 h [64]), transformed into compositions [65], and expressed as centred log-ratios (*CLR*). For this amount, we computed for each interval the geometric mean of the daily compositions.

Each amount has two *families*. The absolute amount has the *(absolute) raw* family (for *energy* expenditure, *steps*, and *heart rate*) and the *(absolute) processed* family (for the durations of *sleep* and physical activity at the four intensities reported by Fitbit: *sedentary*, *light*, *fair*, and *vigorous*). As Fitbit had not provided thresholds for the reported physical activity intensities (see [66,67,68]), we also included cumulative variables of adjacent pairs of intensities, e.g., *light+fair*. Furthermore, we included a total daily *active* duration that added all non-sedentary intensity durations.

The relative amount has the *(relative) centred log-ratio for physical activity* family (*CLR PA*) that adds for each day the durations of physical activity at the four intensities above, and the *(relative) centred log-ratio for physical activity and sleep* family (*CLR PA+S*) that adds for each day the durations of physical activity (four intensities) and sleep.

This procedure corresponds to Step 3B in Figure 1. All variables can be seen in Table 3. Appendix B.2.3 provides details on the variable derivation for TechROs.

#### 2.4.4. Co-Calibration (PROs vs. TechROs)

We co-calibrated PROs with TechROs by alignment. Concretely, for a PRO variable to align to a TechRO variable, the administration date of the former must have been within a set duration (0–120 days) from the end date of the latter.

To account for small samples, we allowed a *leeway* (0–120 days) between the end of the TechRO monitoring interval and the PRO scale administration date.

For each participant, we included only the last alignment in a wave, to discard repeated answers within a few minutes and reduce bias towards overly diligent responders.

When we aligned PROs with TechROs of increasing durations, the number of paired observations decreased; we thus required a minimum of 10 observations to have a nontrivial size [69].

For each PRO-TechRO pair, we reported the highest correlation among all *aggregation intervals* of TechRO (7–120 days) aligned to match the PRO administration date. We included only *significant* correlations, i.e., those correlation coefficients whose 95% confidence interval maintained sign. This procedure corresponds to Step 2 in Figure 1. Appendix B.3 elaborates on the details of the PRO-TechRO variable alignment.

### 2.5. Data Analysis

We conducted descriptive and inferential analyses of the PROs and TechROs. We then analyzed patterns from the analyses.

#### 2.5.1. Descriptive Analysis (PROs and TechROs)

The descriptive analysis consisted of summary statistics (median, mean, and standard deviation, or *SD*) based on *groups* of participant-wave characteristics. In our study, we analyzed the participants by their *health*, *country*, and *gender* self-reported groups. For PROs, we observed the statistics across waves. Appendix B.1 elaborates on the analysis of the PRO variables. For TechROs, we observed the statistics across the entire study period and by counting valid days, described in depth in Appendix B.2. Appendix B.3.1 details the descriptive analysis procedure.

#### 2.5.2. Inferential Analysis (PROs vs. TechROs)

We co-calibrated PRO variables with TechRO variables by applying the Spearman [70] statistical test on each pair of PRO-TechRO variables resulting from the alignments. The Spearman $r_{S}$ statistical correlation coefficient measures the direction and strength of the association between two variables. We used the SciPy library [71] to implement the Spearman correlations. Appendix B.3.2 elaborates on the motivation and assumptions for the inferential analysis. This procedure corresponds to Step 4 in Figure 1.

#### 2.5.3. Pattern Analysis (PROs vs. TechROs)

We used the results from the inferential analysis to highlight informative PRO variables and pairs of PRO-TechRO. This procedure corresponds to Step 5 in Figure 1. We employed two metrics that focus on the number of correlations (a high number of significant correlations with TechRO variables indicates that the PRO variable is informative) and the quality of the correlations (where possible, a strong significant correlation with other significant correlations in its vicinity indicates that the PRO-TechRO correlation is informative).

The first metric, denoted *total*, counts all strong correlations ($r_{S}$ ≥ 0.5) for a given PRO variable and highlights those PRO variables that correlate with the most TechRO variables. We applied this metric to all PRO variables.

The second metric, denoted *contour*, can only apply for variables that can be ordered by a criterion. For our study, we ordered TechRO physical activity variables by their intensities (from *sedentary* to *vigorous*). We applied this metric on strong and significant correlations ($r_{S}$ ≥ 0.8) between a PRO and a TechRO physical activity intensity variable. The metric counted the maximum number of adjacent significant correlations of the same PRO variable (at *lower* and, separately, *higher* intensities) such that they would form a contiguous sequence of significant correlations that maintained the sign. Appendix B.3.3 further explains and exemplifies this metric.

## 3. Results

In this section, we report the results from the study participants (Section 3.1) and analyses (descriptive in Section 3.2, inferential in Section 3.3, and patterns in Section 3.4) as well as two use case examples for coQoL (Section 3.5).

### 3.1. Study Participants

Forty-two seniors (mean age 69.8 ± 7.4) signed up for the study. From these, 39 participants (mean age 70.0 ± 7.2, 22 women, 26 from Spain 26 and 13 from Hungary) provided at least one PRO; three participants were disqualified. Out of the qualified participants, 28 reported no health condition (thus being in the *healthy* health group) and 11 reported a mild health condition (forming the *diseased* health group). Participant characteristics are available in Table 4.

### 3.2. Descriptive Analysis (PROs and TechROs)

#### 3.2.1. Patient-Reported Outcomes (Questionnaires)

Three waves of PRO participation resulted from January 2017 to December 2019: wave 1 (mid-2018), wave 2 (end-2018 and start-2019), and wave 3 (mid-2019). Table 5 illustrates the waves of participation for each participant and questionnaire.

**Figure 2:**
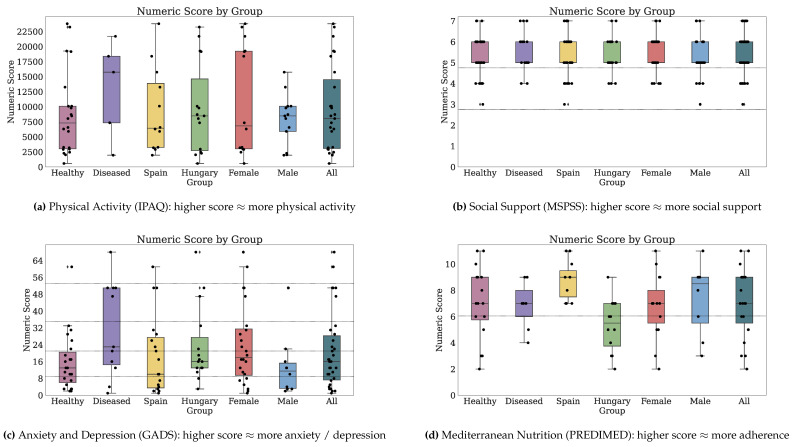
Numeric scores for Physical Activity, Social Support, Anxiety and Depression, and Mediterranean Nutrition. Dotted markings delimit levels of the categorical score, where available (1 of 2).

**Figure 3:**
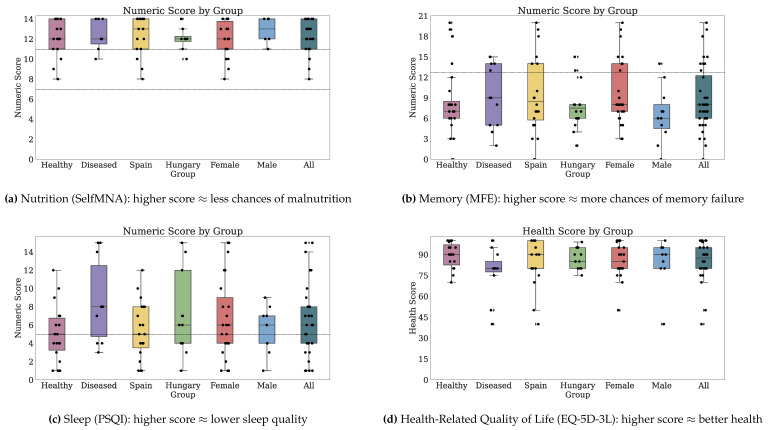
Numeric scores for Nutrition, Memory, Sleep, and Health-Related Quality of Life. Dotted markings delimit levels of the categorical score, where available (2 of 2).

Figure 2 and Figure 3 depict the numeric scores for all patient-reported outcome scales. Appendix B.1 details the results in-depth for each PRO variable.

#### 3.2.2. Technology-Reported Outcomes (Fitbit)

**Figure 4:**
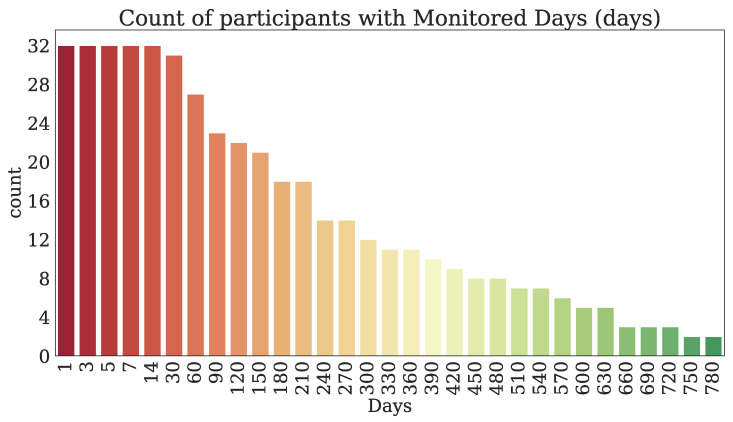
Count of seniors with at least the given monitored days of Fitbit (TechRO).

**Figure 5:**
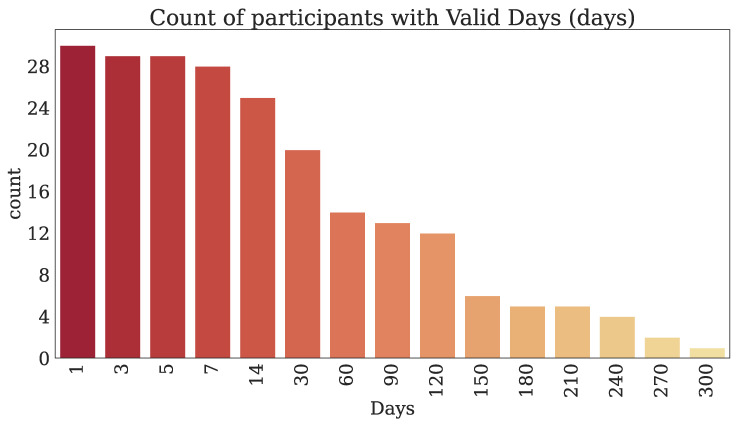
Count of seniors with at least the given valid days of Fitbit (TechRO).

**Figure 6:**
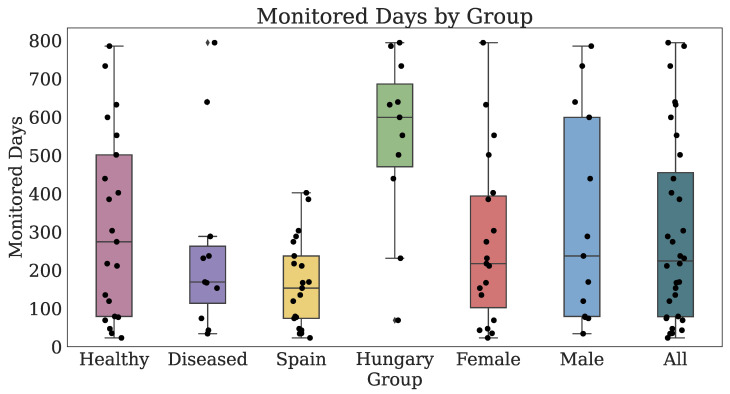
Days of Fitbit (TechRO) monitored days for seniors with at least one PRO.

**Figure 7:**
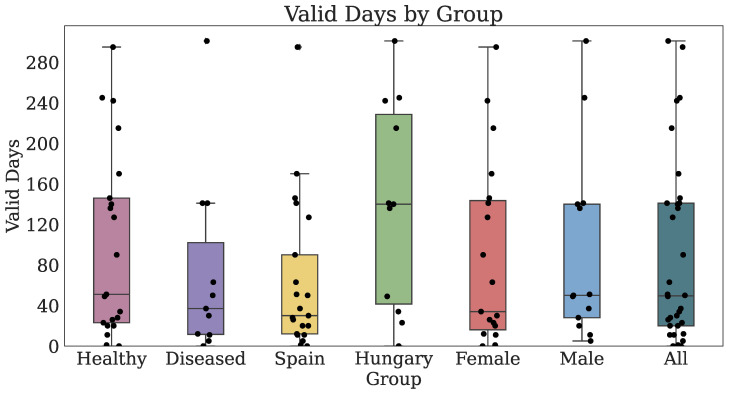
Days of Fitbit (TechRO) valid days data for seniors with at least one PRO.

**Figure 8:**
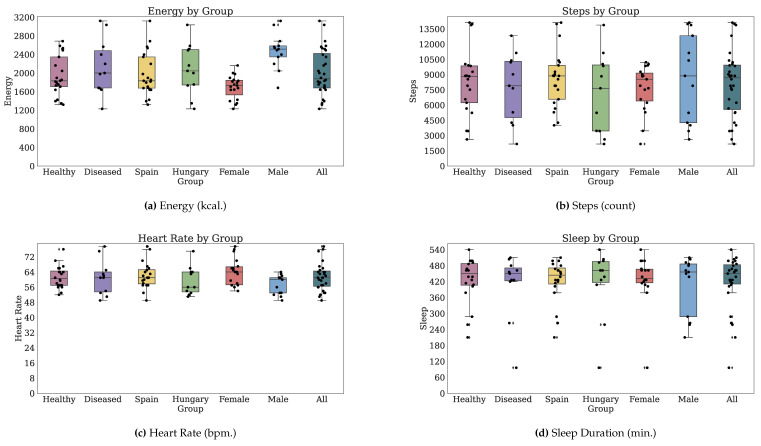
Median values of TechROs (Fitbit) across the entire monitoring period: energy, steps, heart rate, and sleep (1 of 2).

**Figure 9:**
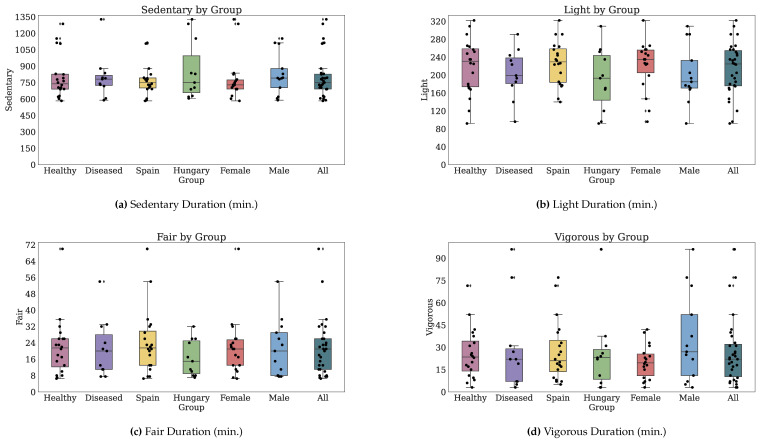
Median values of TechROs (Fitbit) across the entire monitoring period: physical activity (2 of 2).

Thirty-two participants provided both PROs and TechROs. Figure 4 and Figure 5 depict the counts of participants by monitored and valid Fitbit days, respectively. Figure 6 and Figure 7 depict the distribution of monitored and valid Fitbit days, respectively. Figure 8 and Figure 9 depict the medians of TechROs across the entire monitoring period for the participants. Appendix B.2 provides additional details on compliance and analyses each TechRO in-depth.

### 3.3. Inferential Analysis (PROs vs. TechROs)

Appendix C.2 elaborates on the Spearman rank correlations resulted from the inferential analysis on each questionnaire and PRO-TechRO variable pair.

### 3.4. Pattern Analysis (PROs vs. TechROs)

We report further the results of the pattern analysis for each questionnaire: physical activity (Section 3.4.1), social support (Section 3.4.2), anxiety and depression (Section 3.4.3), Mediterranean nutrition (Section 3.4.4), nutrition (Section 3.4.5), memory (Section 3.4.6), sleep (Section 3.4.7), and health-related Quality of Life (Section 3.4.8).

#### 3.4.1. coQoL for Physical Activity (IPAQ vs. Fitbit)

We report the correlations of PRO physical activity variables (IPAQ) with TechRO variables (Fitbit) by using the *total* and *contour* metrics.

##### Physical Activity Outcomes by Total Numbers of Correlations

Table 6 highlights the PROs that correlated with the most TechROs ($r_{S}$ ≥ 0.5) across all TechRO families by health group.

In the health group with all participants, when assessing totals of correlations, PRO *moderate* activity in the *domestic*, *garden*, and *leisure* domains correlated with the most TechROs (Table 6).

In the group with healthy participants, PRO *moderate activity* in the *domestic* and *garden* domains had the most correlations with TechROs as well. The *domestic moderate* and *garden moderate* activity were also the only two PROs highlighted by the total metric in the groups with all and healthy participants.

In the group with diseased participants, PRO *vigorous* in the *garden* and *leisure* domains correlated with the most TechROs, followed by the PRO *moderate* and *vigorous* activities in the *work* domain (Table 6).

##### Physical Activity Outcomes by Contours of Correlations

We report the strong correlations ($r_{S}$ ≥ 0.8) and their contours between PRO variables (IPAQ) and TechRO variables (Fitbit) in Table 7.

In the health group with all participants, when assessing strong correlations, the PRO *domestic moderate* activity had a small contour of correlations with the TechRO *light+fair* physical activity. Also, the PRO *work vigorous* activity may explain the TechRO *active* duration without a contour (Table 7, rows with Health: All).

In the group with healthy participants, only two strong correlations emerged without contours. PRO work moderate and total activity correlated with the TechRO fair activity duration (Table 7, rows with Health: Healthy).

In the group with diseased participants, we found numerous correlations with and without contours in the *work* domain. A positive relationship with a broad contour occurred between PRO *work moderate* activity and TechRO *fair* activity duration. Furthermore, PRO *work moderate* activity correlated negatively with TechRO *sedentary* duration. However, work activity at the two extreme intensities (*walking* and *vigorous*) also correlated negatively with relative *light* activity (Table 7, rows with Health: Diseased and PRO Domain: Work).

For the PRO *garden* domain, PRO *vigorous* activity correlated negatively with contours with TechRO relative *sedentary* and *light* activity, indicating that it may redistribute physical activity across the other intensities over the day (Table 7, rows with Health: Diseased and PRO Domain: Garden).

For the PRO *leisure* domain, *walking* activity correlated without contours with *energy* and *steps*. PRO *leisure vigorous* activity correlated positively with TechRO *fair+vigorous* activity durations and negatively with TechRO absolute *sedentary* and relative *light* durations. The PRO *leisure total* activity had a correlation with contour consistent with the previous correlation: negative relationship with TechRO *sedentary+light* activity (Table 7, rows with Health: Diseased and PRO Domain: Leisure).

The PRO *vigorous* activity in the *work* domain appeared in both groups with all and diseased participants. However, its correlations were divergent: for all participants, the *work vigorous* associated with the total daily *activity*, while for the mildly diseased, it may replace *light* activity. The *moderate activity* at *work* had inverse relations with *fair* activity for diseased (positive) and healthy (negative) participants. However, for the diseased, the correlation had a broad contour, while for the healthy it had none. In this case, the latter relation may have been a false positive (Table 7, rows with PRO Domain: Work).

Across numerous PROs, the TechRO of *sedentary* activity correlated strongly only for diseased participants and mostly in relative families. PRO *moderate* to *vigorous* activity at *work*, in the *garden*, and for *leisure* all negatively correlated with TechRO daily *sedentary* duration. These results indicate that *moderate* activity may contribute to lower measured TechRO *sedentary* duration, but the redistributions of daily time to other TechRO intensities may vary between TechRO *fair* and *vigorous* intensities. (Table 7, rows with Health: Diseased and TechRO Variable: Sedentary).

##### Physical Activity Outcomes Highlighted by Both Metrics

For the health group with all participants, the *domestic moderate* activity appeared with both metrics. This result is in concordance with the strong correlations in the PRO *domestic* domain mentioned above (Table 6 and Table 7, rows with Health: All).

In the group with diseased participants, the total metric results confirmed those using the contour metric for the PRO *work* domain at *moderate* and *vigorous* intensities (Table 6 and Table 7, rows with Health: Diseased).

##### Physical Activity Outcomes Interpretation

In the health group with all participants, we observed several “expected” correlations. The PRO *domestic moderate* activity associated with the TechRO absolute *light+fair* activity duration. This effect is only visible for the total metric, indicating that PRO *domestic* and *garden moderate* activity may redistribute physical activity across numerous TechRO intensities.

In the group with diseased participants, PRO *work moderate* associated with the TechRO absolute *fair* activity duration. For the same health group, *leisure walking* activity correlated with both *energy* and *steps*, while PRO *vigorous* activity correlated with both absolute *fair+vigorous* activity and relative *vigorous* activity (when including sleep).

In this group, we also found “expected” correlations between PROs and TechRO *sedentary* duration. PRO *moderate* activity at *work*, *vigorous* activity in the *garden*, and *vigorous* activity for *leisure* associated negatively with TechRO *sedentary* duration. The TechRO *sedentary+light* duration associated negatively with the PRO *total active* effort as well.

Other associations indicate potential activity replacements (within TechRO) for the same health group (diseased). Walking at *work* associated negatively with the relative duration of activity at the *light* intensity, indicating that, when they *walk* at *work*, they tend to perform less *light* activity elsewhere. Also, the *vigorous* activity effort may replace *light* activity duration during the day, indicating that the participants tend to limit their physical activity to a narrow spectrum of intensities.

The distribution of results per families of TechROs indicates that for the groups with all participants and the healthy, the absolute families may provide most, if not all, strong correlations. However, for the diseased group, measuring the entire physical activity duration and including sleep uncovered associations weaker or non-significant otherwise. For this group, measuring only raw *energy* or *steps* TechROs may be indicative of their *leisure walking* efforts, potentially useful for more sedentary participants who do not work.

Both metrics highlighted all IPAQ domains except *transport*. The PRO *transport* physical activity was not indicative of TechRO physical activity measures, potentially due to the lower and fewer correlations with *transport*. However, the raw responses indicate that *transport walking* activity may associate with the *numeric score* of physical activity.

#### 3.4.2. coQoL for Social Support (MSPSS vs. Fitbit)

We report the correlations of PRO social support variables (MSPSS) with TechRO variables (Fitbit) by using the *total* and *contour* metrics.

##### Social Support Outcomes by Total Numbers of Correlations

Table 6, rows with Outcome: Social Support, enumerates the PROs that correlated with the most TechROs ($r_{S}$ ≥ 0.5) across all families by health group.

In the health group with all participants, PRO *family* items Q8 (*talks about problems*) and Q11 (*willing to help make decisions*) correlated with the most TechROs.

In the group with healthy participants, PRO *friends* items, Q6 (*friends try to help*), Q9 (*friends share joys and sorrows*), and Q12 (*friends talk about problems*), had relatively more correlations with TechRos than PRO *significant other* or *family* items. Furthermore, the PRO *friends numeric score* had many correlations with TechROs.

In the group with diseased participants, PRO *family* Q4 (*family gives emotional help and support*) correlated negatively with TechRO absolute sedentary duration and Q12 (*friends talk about problems*) positively with the TechRO *steps* (Table 8, rows with Health: Diseased).

##### Social Support Outcomes by Contours of Correlations

We report the strong correlations ($r_{S}$ ≥ 0.8) and their contours between PRO variables (MSPSS) and TechRO variables (Fitbit) in Table 8.

In the health group with all participants, several PRO items related to the *significant other* social support, Q2 (*a special person shares joys and sorrows*), Q5 (*a special person is a real source of comfort*), and Q10 (*a special person cares about my feelings*) correlated strongly and with a broad contour with TechRO relative *vigorous* activity durations when including sleep (Table 8, rows with Health: All and PRO Source: Significant other). Also, several PRO *family* items, Q3 (*family tries to help*) and Q8 (*family talks about problems*) as well as the *family numeric sub-score* correlated strongly and with a broad contour with TechRO relative *fair* and *vigorous* activity durations when including sleep. These two strong co-calibrations only appeared as highlighted in the CLR PA+S family (Table 8, rows with Health: All and PRO Source: Family).

In the group with healthy participants, we observed numerous strong negative correlations with broad contours between numerous PRO items. Several are related to the *significant other* source: Q1 (*a special person is around when in need*), Q2 (*a special person shares joys and sorrows*), Q5 (*a special person is a real source of comfort*), and Q10 (*a special person cares about my feelings*) as well as the *significant other numeric sub-score* and the TechRO *fair* physical activity duration. However, we also observed a strong, positive correlation with a similarly sized contour with PRO item Q5 (*a special person is a real source of comfort*) and TechRO *fair* activity duration in the relative CLR PA+S family. These results indicate that measuring daily sleep is necessary to co-calibrate this PRO source and TechRO physical activity intensity (Table 8, rows with Health: Healthy and PRO Source: Significant other).

Also, several PRO *family* items, Q3 (*family tries to help*), Q8 (*family talks about problems*), and Q11 (*family is willing to help make decisions*) correlated negatively with TechRO absolute *fair* activity, but positively with the relative duration at the same physical activity intensity (Table 8, rows with Health: Healthy and PRO Source: Family), yielding a similar interpretation.

Few PRO *friends* items such as Q9 (*friends share joys and sorrows*) and Q12 (*friends talk about problems*) correlated with broad contours with the TechRO absolute *light* physical activity duration (Table 8, rows with Health: Healthy and PRO Source: Friends).

Also, the PRO *categorical score* strongly correlated without contour with the TechRO absolute daily duration of physical activity (*active*) and the relative CLR PA *light* activity. The PRO *numeric score* also correlated with the TechRO absolute *light+fair* activity and relative CLR PA+S *fair* activity, indicating a positive relationship between social support and light to fair activity (Table 8, rows with Health: Healthy and PRO Source: All).

In the group with diseased participants, we only observed two isolated strong correlations. PRO *family* item Q4 (*gives emotional help and support*) correlated negatively with TechRO *sedentary* duration. PRO *friends* item Q12 (*talk about problems*) correlated positively with daily *steps* (Table 8, rows with Health: Diseased).

PRO items Q2, Q3, Q5, Q8, Q10, and the *numeric score* appeared in both groups of all and healthy participants. However, only Q8 maintained the correlation with TechRO *fair* physical activity across health groups. Q12 had strong correlations in both groups of healthy and diseased participants. However, the relationship was expressed through separate outcomes: *light* activity and *steps*, respectively (Table 8).

##### Social Support Outcomes Highlighted by Both Metrics

In the health group with all participants, PRO *friends* Q9 (*friends share joys and sorrows*) and Q12 (*friends talk about problems*) were highlighted as strongly correlated by both contour and total metrics, and thus informative for co-calibration with TechROs (Table 6 and Table 8, rows with Health: All).

In the group with healthy participants, for the *significant other* and *family* sources of social support, Q10 (*a special person cares about my feelings*) and Q3 (*family tries to help*) appeared as informative with both metrics (Table 6 and Table 8, rows with Health: Healthy).

##### Social Support Outcomes Interpretation

In the health group with all participants, several PRO items related to the *significant other* and *family* social support. They alternatively correlated with TechRO relative *fair* and *vigorous* activity: *family* items to the *fair* activity, and *significant other* items to the *vigorous* activity. All correlations resulted from relative TechROs including sleep. For this reason, the assessment of social support may benefit from the inclusion of sleep in the analysis.

In the group with healthy participants, the PRO social support from the *significant other* had negative correlations with TechRO *fair* activity in the absolute amount and positive correlations with *fair* activity in the relative amount (including sleep). This pattern was also pronounced for the items related to *family* social support. Sleep changed the ordering of durations throughout the day across the healthy participants. We argue for including sleep in the analysis of *significant other* and *family* social support for healthy seniors. Having *friends* who *share joys and sorrows* and, in general, *talk about problems*, associated with more light activity.

In the group with diseased participants, *emotional help* and *support* from the *family* associated with less *sedentary* time throughout the day. Also, having *friends* who *talk about problems* associated with more *steps*.

In general, the *significant other being a real source of comfort* appeared in most instances, followed by *having someone who cares about feelings*, then having someone who *shares joys and sorrows*, and then (at a distance) having a special person *around when in need*. Having a *significant other* who is *a source of comfort* may serve as a proxy item for more frequent assessments of the relationships between *significant other* social support and physical activity at the *fair* to *vigorous* intensities.

Having a *family* that *tries to help*, *talks about problems*, and *wishes to help make decisions* appeared in three groups across metrics. However, *getting emotional help and support* from the family only appeared once. Frequent administrations of the MSPSS may choose to assess the relationships between *family* social support and *fair* physical activity by using only the first three items.

Having *friends* with whom to *talk about problems* appeared in three groups across metrics. Having *friends* who *try to help* and *share joys and sorrows* appeared less often with strong correlations and contours but had numerous correlations in total. We argue that *counting on friends when things go wrong* is a less prominent item in assessing relationships between *friends* social support and physical activity.

#### 3.4.3. coQoL for Anxiety and Depression (GADS vs. Fitbit)

We report the correlations of PRO anxiety and depression (GADS) with TechRO variables (Fitbit) by using the *total* and *contour* metrics.

##### Anxiety and Depression Outcomes by Total Numbers of Correlations

Table 6, rows with Outcome: Anxiety and depression, enumerates the PROs that correlated with the most TechROs ($r_{S}$ ≥ 0.5) across all families by health group.

In the health group with all participants, PRO *anxiety* item Q8A (*worried about own health*), as well as PRO *depression* items Q1D (*lacking energy*) and Q6D (*lost weight due to poor appetite*), recorded the most correlations with TechROs (Table 6, row with Outcome: Anxiety and depression, Health: All).

In the group with healthy participants, PRO item Q2D (*lost interest in things*) had the most correlations (Table 6, row with Outcome: Anxiety and depression, Health: Healthy).

In the group with diseased participants, PRO item Q2A (*worrying a lot*) had the most correlations with TechROs (Table 6, row with Outcome: Anxiety and depression, Health: Diseased).

##### Anxiety and Depression Outcomes by Contours of Correlations

We report the strong correlations ($r_{S}$ ≥ 0.8) and their contours between PRO variables (GADS) and TechRO variables (Fitbit) in Table 9.

In the health group with all participants, PRO *anxiety* item Q5A (*sleeping poorly*) correlated strongly with a broad contour with TechRO relative CLR PA+S *light* physical activity. We found other isolated correlations for *anxiety*. PRO item Q3A (*irritable*) correlated with the TechRO relative *vigorous* activity. PRO item Q7A (*trembling [...]*) negatively correlated with the TechRO daily *active* duration. PRO *depression* items Q1D (*lacking energy*) and Q6D (*lost weight due to poor appetite*) had isolated correlations. The PRO *numeric score* had a strong correlation with the TechRO relative *sleep* duration (Table 9, rows with Health: All).

In the group with healthy participants, PRO *anxiety* item Q7A (*trembling [...]*) correlated positively with TechRO *vigorous* activity and negatively with TechRO *light* and *light+fair* activity durations (the last with a broad contour) in both absolute and relative families. PRO item Q7A correlated negatively with the total daily *active* duration. PRO item Q3A (*irritable*) correlated negatively with total daily *active* duration. PRO *depression* items Q2D (*lost interest in things*) and Q9D (*worse in the morning*) had isolated correlations, the first negative with TechRO relative CLR PA *light* activity duration, and the second with TechRO relative CLR PA+S *sedentary* duration. PRO item Q6D (*lost weight due to poor appetite*) recorded a positive correlation as well, with TechRO relative *sleep* duration (Table 9, rows with Health: Healthy).

In the group with diseased participants, we did not observe strong correlations ($r_{S}$ ≥ 0.8) by using the contour metric (Table 9, rows with Health: Diseased).

PRO items Q3A, Q7A, and Q6D appeared in both groups with all and healthy participants. However, only Q7A kept the same strong correlation against total daily *active* duration in the two groups (Table 9).

##### Anxiety and Depression Outcomes Highlighted by Both Metrics

In the health group with all participants, PRO items Q1D (*lacking energy*) and Q6D (*lost weight due to poor appetite*) were highlighted by both metrics (Table 6 and Table 9, rows with Health: All).

For healthy participants, PRO item Q2D (*lost interest in things*) appeared in both metrics as well (Table 6 and Table 9, rows with Health: Healthy).

##### Anxiety and Depression Outcomes Interpretation

In the health groups with all and healthy participants, *irritability* and *trembling* may expediently assess *anxiety* while having *lost interest in things* and *losing weight due to poor appetite* may assess *depression*. Follow-up investigations may establish whether the health state is momentary or deteriorating over time.

PRO *Trembling, tingling, dizziness, sweating, diarrhoea, or passing urine* yielded numerous correlations for healthy participants: negative correlations with TechRO *light*, *light+fair*, and total daily *active* duration as well as a positive correlation with *vigorous* physical activity duration. When a daily life monitor observed a gradual replacement of *light* to *fair* activity with *vigorous* activity (as reported by the wearable), it may be worth investigating whether an otherwise healthy participant also becomes gradually more anxious (by using items).

In the group with healthy participants, a decrease in *light* physical activity may indicate that the participants experience an increase in *depression*. Researchers can then assess this hypothesis by administering, e.g., the corresponding item in the EQ-5D-3L scale. A similar process could be employed for all seniors by longitudinally monitoring the *sleep* duration relative to the 24 h of the day, based on the corresponding strong correlations between the *numeric score* and the relative *sleep* duration. In the case of increasingly longer *sleep*, the participant may enter a state of *anxiety* or *depression*.

In general, *depression* and *anxiety* positively associated with the *sedentary* duration, in both absolute and relative TechRO families, especially for participants who self-report disease. The two items in the scale referring to sleep may provide additional insights towards not only the anxiety and depression status of the participant, but also sleep quality.

#### 3.4.4. coQoL for Mediterranean Nutrition (PREDIMED vs. Fitbit)

We report the correlations of PRO Mediterranean nutrition variables (PREDIMED) with TechRO variables (Fitbit) by using the *total* and *contour* metrics.

##### Mediterranean Nutrition Outcomes by Total Numbers of Correlations

Table 6, rows with Outcome: Mediterranean nutrition, enumerates the PROs that correlated with the most TechROs ($r_{S}$ ≥ 0.5) across all families by health group.

In the health group with all participants, the PRO *categorical score*, *numeric score* and items Q12 (*nuts use*) and Q14 (*sofrito use*) had the most correlations with TechROs (Table 6, rows with Outcome: Mediterranean nutrition, Health: All).

In the group with healthy participants, PRO item Q4 (*fruit use*) and the *categorical score* had the most correlations with TechROs (Table 6, rows with Outcome: Mediterranean nutrition, Health: Healthy).

In the group with diseased participants, we only observed PROs with reduced numbers of correlations with TechROs across families (Table 6, rows with Outcome: Mediterranean nutrition, Health: Diseased).

The *categorical score* is the only PRO that appeared with numerous correlations in the two groups with all and healthy participants (Table 6).

##### Mediterranean Nutrition Outcomes by Contours of Correlations

We report the strong correlations ($r_{S}$ ≥ 0.8) and their contours between PRO variables (PREDIMED) and TechRO variables (Fitbit) in Table 10.

In the health group with all participants, PRO item Q12 (*nuts use*) had an isolated negative correlation with the TechRO absolute *fair* activity, but a positive correlation (with a contour) with the TechRO relative CLR PA+S *light* activity. The PRO *numeric score* also registered two correlations with contours: negative with TechRO absolute *vigorous* activity duration and positive with TechRO relative CLR PA+S *light* activity duration (Table 10, rows with Health: All).

In the group with healthy participants, PRO item Q3 (*vegetables use*) correlated negatively with the TechRO relative *fair* activity in both CLR PA and CLR PA+S families (Table 10, rows with Health: Healthy). While the two correlations had no contour, their presence in both families highlights an effect.

In the group with diseased participants, PRO item Q5 (*red meat, hamburger, or meat use*) correlated positively with TechRO *energy* expenditure. For the same group, PRO item Q11 (*commercial sweets or pastries use*) correlated positively with TechRO *heart rate* (Table 10, rows with Health: Diseased).

##### Mediterranean Nutrition Outcomes Highlighted by Both Metrics

For all participants, PRO item Q12 (*nuts use*) and the *numeric score* were highlighted by both metrics (Table 6 and Table 10, rows with Health: All).

##### Mediterranean Nutrition Outcomes Interpretation

In the health group with all participants, the nutrition *numeric score* associated with the relative *sleep* duration, and *using nuts* had a similar correlation (both correlations with contours). Further studies may assess whether this item can be administered independently of the full scale (for the *numeric score*) to assess the relationship between (mal)nutrition and *light* physical activity in seniors.

With regards to poor nutrition choices and their potentially magnified effects on people with mild disease, the *consumption of red meat and hamburgers* by participants with mild disease correlated with higher *energy* expenditure. The consumption of *commercial sweets or pastries* also associated with an increased *heart rate*.

The PRO *numeric* and *categorical scores* correlated with numerous TechROs, indicating a replacement of *fair* to *vigorous* activity with the *light* activity.

Participants from Spain had on average more adherence than those from Hungary (Appendix C.1.1), making the country of residence a potential confounder for the relationships above.

#### 3.4.5. coQoL for Nutrition (SelfMNA vs. Fitbit)

We report the correlations of PRO nutrition variables (SelfMNA) with TechRO variables (Fitbit) by using the *total* and *contour* metrics.

##### Nutrition Outcomes by Total Numbers of Correlations

Table 6, rows with Outcome: Nutrition, enumerates the PROs that correlated with the most TechROs ($r_{S}$ ≥ 0.5) across all families by health group.

For all health groups, we found PROs correlated with few TechROs when compared to other outcomes (Table 6, row with Outcome: Nutrition, Health: All).

In the groups with all participants and the healthy, the PRO *categorical score* had the most correlations (Table 6, row with Outcome: Nutrition, Health: Healthy).

In the group with diseased participants, PRO items Q1 (*food intake declined*) and Q2 (*weight lost*) recorded the most correlations with TechROs (Table 6, row with Outcome: Nutrition, Health: Diseased).

The *categorical score* is the only PRO that appeared in two health groups: the group with all participants and the group with healthy participants (Table 6).

##### Nutrition Outcomes by Contours of Correlations

We report the strong correlations ($r_{S}$ ≥ 0.8) and their contours between PRO variables (SelfMNA) and TechRO variables (Fitbit) in Table 11.

We only found strong correlations ($r_{S}$ ≥ 0.8) in the group with diseased participants. PRO items Q1 (*food intake declined*) and Q2 (*weight lost*) correlated negatively with the TechRO relative *sleep* duration. PRO item Q4 (*stressed or severely ill*) correlated negatively with the TechRO absolute *sedentary* duration (Table 11).

##### Nutrition Outcomes Highlighted by Both Metrics

In the group with diseased participants, PRO items Q1 (*food intake declined*) and Q2 (*weight lost*) were highlighted by both metrics (Table 6 and Table 11, rows with Health: Diseased).

##### Nutrition Outcomes Interpretation

In the health group with all participants, the PRO *categorical score* correlated with numerous TechROs. In general, better nutrition coincided with less *sedentary* and light *physical* activity and more *fair* and *vigorous* physical activity. In the group with healthy participants, both *numeric* and *categorical scores* exhibited this pattern (Appendix C.2).

In the group with diseased participants, a long-term decrease in *sleep* duration may indicate a *decline in food intake* or a *loss of weight*—two outcomes that appeared in both metrics and may lead to malnutrition.

#### 3.4.6. coQoL for Memory (MFE vs. Fitbit)

We report the correlations of PRO memory variables (MFE) with TechRO variables (Fitbit) by using the *total* and *contour* metrics.

##### Memory Outcomes by Total Numbers of Correlations

Table 6, rows with Outcome: Memory, enumerates the PROs that correlated with the most TechROs ($r_{S}$ ≥ 0.5) across all families by health group.

In the health group with all participants, the PRO items that correlated with the most TechROs were Q12 (*having difficulty picking up a new skill*), Q14 (*forgetting to do planned things*), and Q6 (*forgetting the time of events*) (Table 6, rows with Outcome: Memory and Health: All).

In the group with healthy participants, PRO items Q6 (*forgetting the time of events*), Q15 (*forgetting details of done things*), Q12 (*having difficulty picking up a new skill*), and Q14 (*forgetting to do planned things*) correlated with the most TechROs (Table 6, rows with Outcome: Memory and Health: Healthy).

In the group with diseased participants, PRO items Q13 (*having a word on the tip of the tongue*) and Q25 (*getting lost in often visited place*) had the most correlations (Table 6, rows with Outcome: Memory and Health: Diseased).

PRO items Q12 (*having difficulty picking up a new skill*) and Q14 (*forgetting to do planned things*) were the only outcomes that had numerous correlations with TechROs across two groups: all and healthy (Table 6).

##### Memory Outcomes by Contours of Correlations

We report the strong correlations ($r_{S}$ ≥ 0.8) and their contours between PRO variables (MFE) and TechRO variables (Fitbit) in Table 12.

In the health group with all participants, there was only one strong correlation with contour between PRO item Q24 (*forgetting where things are normally kept*) and PRO *fair* activity in the CLR PA family. The PRO *numeric score* had a negative correlation with the TechRO total daily *active* duration. PRO item Q7 (*completely forgetting to take things*) had a strong correlation with TechRO relative *sleep* duration. PRO items Q12 (*having difficulty picking up a new skill*) and Q13 (*finding a word on the tip of the tongue*) had negative and positive relations with TechRO relative *light* and *fair* CLR PA+S activity durations, respectively (Table 12, rows with Health: All).

In the group with healthy participants, PRO item Q14 (*forgetting to do planned things*) had a contour of two strong correlations with TechRO *fair+vigorous* and *vigorous* activity. PRO item Q16 (*forgetting the topic of an ongoing conversation*) had a strong correlation with contour TechRO absolute *fair* activity duration. PRO items Q10 (*letting ramble about unimportant things*) and Q24 (*forgetting where things are normally kept*) had isolated negative correlations with TechRO *fair* activity duration. PRO item Q7 (*completely forgetting to take things*) recurred in correlating strongly with *sleep*. The *numeric score* also correlated negatively with TechRO relative CLR PA *fair* activity duration (Table 12, rows with Health: Healthy).

In the group with diseased participants, PRO item Q18 (*forgetting to tell somebody something important*) had a broad contour with the TechRO *fair*, *fair+vigorous*, and *vigorous* physical activity duration. PRO item Q6 (*forgetting the time of events*) had a positive correlation with the TechRO *heart rate*, a positive correlation (having a contour) with the *light* activity, and a negative correlation with the *sleep* duration. PRO item Q1 (*forgetting objects put*) had a negative correlation (contour) with the TechRO relative *vigorous* activity in the PA+S family. Q13 (*finding a word on the tip of the tongue*) correlated negatively with TechRO daily *active* duration and positively with relative *sedentary* duration in the CLR PA+S family. Q8 (*being reminded about things*) had a positive correlation with the TechRO *light+fair* activity duration. The PRO *numeric score* correlated negatively with the TechRO total *active* duration (Table 12, rows with Health: Diseased).

PRO items Q7 (*completely forgetting to take things*) and Q24 (*forgetting where things are normally kept*), as well as the *numeric score*, appeared in both groups with all and healthy participants. Items Q7 and Q24 maintained the strong correlations between groups: positive with *sleep* duration and negative with relative *fair* activity. The *numeric score* expressed the inverse relation with physical activity in different ways depending on the health status. For all participants and the mildly diseased, it had a negative correlation with the total daily *active* duration. For the healthy participants, it had a negative correlation with the relative *fair* activity duration (Table 12).

##### Memory Outcomes Highlighted by Both Metrics

In the health group with all participants, Q12 (*having difficulty picking up a new skill*) was highlighted by both metrics as an informative PRO for memory (Table 6 and Table 12, rows with Health: All).

In the group with healthy participants, PRO item Q14 (*forgetting to do planned things*) was informative in both metrics (Table 6 and Table 12, rows with Health: Healthy).

In the group with diseased participants, PRO item Q13 (*finding a word on the tip of the tongue*) was informative through both metrics (Table 6 and Table 12, rows with Health: Diseased).

##### Memory Outcomes Interpretation

In the health group with all participants, the memory *numeric score* strongly associated with shorter durations of any physical activity during the day. A negative correlation with relative *fair* physical activity also reflected this pattern in the group with healthy participants. A decrease in *active* duration may provide an opportunity for a long-term monitoring system to assess whether an otherwise healthy senior is experiencing a gradual increase in memory failures.

In the groups with all participants and the healthy, *forgetting where things are normally kept* associated positively with *fair* physical activity; however, only when accounting for sleep as well.

In the group with diseased participants, *forgetting to tell somebody something important* associated with numerous TechROs, suggesting a replacement of *fair* and *vigorous* activity durations with *sedentary* and *light* duration throughout the day. By observing this TechRO pattern longitudinally in time, a study may administer this item towards assessing memory failures. *Finding a word is on the tip of the tongue* is another PRO item that also correlated with TechRO *sedentary* duration and negatively correlated with daily *active* duration. Further research may investigate the reliability of a more frequent assessment than the MFE scale consisting of the items above for seniors with mild disease.

#### 3.4.7. coQoL for Sleep (PSQI vs. Fitbit)

We report the correlations of PRO sleep variables (PSQI) with TechRO variables (Fitbit) by using the *total* and *contour* metrics.

##### Sleep Outcomes by Total Numbers of Correlations

Table 6, rows with Outcome: Sleep, enumerates the PROs that correlated with the most TechROs ($r_{S}$ ≥ 0.5) across all families by health group.

In the health group with all participants, PRO items Q7 (*trouble staying awake driving, eating, socialising*) and Q4 (*duration of actual sleep*), followed by the *daily dysfunction numeric sub-score*, had the most correlations with TechROs across families (Table 6, rows with Outcome: Sleep and Health: All).

In the group with healthy participants, PRO items Q4 (*duration of actual sleep*), Q5C (*trouble sleeping due to using the bathroom*), Q7 (*trouble staying awake driving, eating, socialising*) had the most correlations with TechROs, followed by the *daily dysfunction numeric sub-score* (Table 6, rows with Outcome: Sleep and Health: Healthy).

In the group with diseased participants, the PROs that correlated with the most TechROs had relatively fewer correlations. The *daily dysfunction numeric sub-score* and Q6 (*duration of actual sleep*) registered the most correlations (Table 6, rows with Outcome: Sleep and Health: Diseased).

The PRO *daily dysfunction numeric sub-score* had numerous correlations in all three health groups. The PRO item Q4 (*duration of actual sleep*) appeared in the groups with all participants and the healthy (Table 6).

##### Sleep Outcomes by Contours of Correlations

We report the strong correlations ($r_{S}$ ≥ 0.8) and their contours between PRO variables (PSQI) and TechRO variables (Fitbit) in Table 13.

In the health group with all participants, PRO *sleep disturbance* item Q5A (*trouble sleeping due to not getting to sleep*) correlated positively with TechRO relative *sleep* duration. PRO items Q5E (*trouble sleeping due to coughing or snoring loudly*) and Q5F (*trouble sleeping due to feeling too cold*) correlated with TechRO relative *vigorous* activity duration (negative, CLR PA family) and *light* activity duration (positive, CLR PA+S family), respectively. PRO item Q7 (*trouble staying awake while driving, eating, socialising*) correlated negatively with TechRO relative *sleep* duration and *light* activity durations. Two *numeric sub-scores* yielded correlations with relative *sleep*: *latency* (positive) and *daily dysfunction* (negative). The *daily dysfunction numeric sub-score* also correlated with TechRO *vigorous* activity (broad contour) and the relative *light* activity (contour). The *efficiency numeric sub-score* had an isolated correlation with TechRO *fair* activity (Table 13, rows with Health: All).

In the group with healthy participants, numerous PROs correlated with TechRO *sleep*: Q2 (*duration to fall asleep*), Q5A (*trouble sleeping due to not getting to sleep*), Q11 (*duration stayed in bed*), and the *latency numeric sub-score*. Among the *sleep disturbance* items, Q5C (*trouble sleeping due to using the bathroom*) had two contoured correlations: negative with *light+fair* and *light* activity (the latter with a broad contour) in absolute and relative CLR PA families, respectively. The PRO *efficiency numeric sub-score* correlated again with TechRO *fair* activity. The *numeric score* correlated positively (and having a contour) with *fair+vigorous* activity (Table 13, rows with Health: Healthy).

In the group with diseased participants, PRO item Q4 (*duration of actual sleep*) registered a broad contour of 3 strong correlations (including $r_{S}$ = 0.9) with *fair*, *fair+vigorous*, and *vigorous* TechRO absolute durations. PRO item Q1 (*time gone to bed at night*) correlated inversely with the TechRO absolute *sleep* duration. *Sleep disturbance* items Q5B (*trouble sleeping due to waking up in the middle of the night*) and Q5C (*trouble sleeping due to using the bathroom*) correlated negatively with *energy* expenditure (Table 13, rows with Health: Diseased).

PRO items Q5A (*trouble sleeping due to not getting to sleep*) and Q5E (*trouble sleeping due to coughing or snoring loudly*), and the *latency* and *efficiency numeric sub-scores* appeared for the groups with all participants and the healthy. Q5A and the *latency numeric sub-score* maintained a strong correlation with the TechRO *sleep* duration. The *efficiency numeric sub-score* maintained the strong correlation with the *fair* activity. Q5E had an inverse relation with TechRO physical activity across these two groups, but expressed through negative correlations with the relative *vigorous* duration and the relative *light* duration, respectively. Q5C (*trouble sleeping due to using the bathroom*) was highlighted in both healthy and diseased groups, but expressed an inverse relation with physical activity through different outcomes: *light-fair* activity duration and *energy* expenditure, respectively (Table 13).

##### Sleep Outcomes Highlighted by Both Metrics

In the health group with all participants, PRO item Q7 (*trouble staying awake driving, eating, socialising*) appeared as informative in both metrics (Table 6 and Table 13, rows with Health: All).

In the group with healthy participants, Q5C (*trouble sleeping due to using the bathroom*) was an informative PRO item that appeared in both metrics (Table 6 and Table 13, rows with Health: Healthy).

##### Sleep Outcomes Interpretation

Several PRO items strongly correlated with sleep-specific TechROs. In the health group with all participants, *having trouble sleeping due to not being able to get to sleep* as well as the *sleep latency numeric sub-score* correlated with relative *sleep* duration while *having trouble staying awake while driving, eating, or socialising* as well as the *daily dysfunction numeric sub-score* correlated negatively with relative *sleep* duration. In the group with healthy participants, the *duration to fall asleep*, *having trouble sleeping due to not getting to sleep*, the *duration to stay in bed*, and the *latency numeric sub-score* correlated with longer relative *sleep* during the day. In the group with diseased participants, only the *time gone to bed at night* correlated negatively with absolute *sleep* duration. Studies assessing sleep in healthy adults may benefit from the monitoring of the entire day, not only the sleep duration, to find a higher amount of significant outcomes.

In the health group with all participants, PRO decreased sleep quality correlated negatively with TechRO relative *light* and *vigorous* activity. In the group with healthy participants, the *sleep efficiency numeric sub-score* correlated with the relative *fair* activity, and *using the bathroom* correlated negatively with relative *light* physical activity (with a broad contour). In the group with diseased participants, the *duration of actual sleep* correlated with absolute *fair*, *fair+vigorous*, and *vigorous* durations. *Having trouble sleeping due to waking up in the middle of the night* may be an indicator of already low sleep quality in participants with mild disease.

#### 3.4.8. coQoL for Health-Related Quality of Life (EQ-5D-3L vs. Fitbit)

We report the correlations of PRO health-related Quality of Life variables (EQ-5D-3L) with TechRO variables (Fitbit) by using the *total* and *contour* metrics.

##### Health-Related Quality of Life Outcomes by Total Numbers of Correlations

Table 6, rows with Outcome: Quality of Life, enumerates the PROs that correlated with the most TechROs ($r_{S}$ ≥ 0.5) across all families by health group.

In the health group with all participants, the PRO items with the most correlations were the *health score* and Q4 (*pain/discomfort*). The items in this scale had relatively fewer correlations than the other scales such as social support (MSPSS) or memory (MFE) (Table 6, rows with Outcome: Quality of Life and Health: All).

In the group with healthy participants, PRO item Q4 (*pain/discomfort*) had the most correlations with TechROs (Table 6, row with Outcome: Quality of Life and Health: Healthy).

In the group with diseased participants, PRO item Q5 (*anxiety/depression*) had the most correlations with TechROs (Table 6, row with Outcome: Quality of Life and Health: Diseased).

Q4 (*pain/discomfort*) was the only PRO item that appeared in two groups: the group with all participants and the group with the healthy (Table 6).

##### Health-Related Quality of Life Outcomes by Contours of Correlations

We report the strong correlations ($r_{S}$ ≥ 0.8) and their contours between PRO variables (EQ-5D-3L) and TechRO variables (Fitbit) in Table 14.

We only found one strong correlation in the group of participants with mild disease, between the PRO *depression and anxiety* item (Q5) and the TechRO absolute *sedentary* duration (Table 14).

##### Health-Related Quality of Life Outcomes Highlighted by Both Metrics

In the group with diseased participants, Q5 (*anxiety/depression*) recurred in both metrics (Table 6 and Table 14, rows with Health: Diseased).

##### Health-Related Quality of Life Outcomes Interpretation

The PRO *health state today* correlated with numerous TechROs, in particular with a replacement of *vigorous* physical activity duration with *sleep*, *sedentary*, and *fair* durations across all participants, with a replacement of *fair* and *vigorous* durations with *light* activity for the healthy, and with a decrease in *fair* and *vigorous* activity among the diseased (Appendix C.2).

*Pain and discomfort* also had numerous correlations with TechROs, but only for the groups with all participants and the healthy. In participants with mild disease, having *anxiety/depression* correlated with *sedentary* physical activity. An increase in sedentary duration for participants with already existing mild disease may be an indication of decreased quality of life on the *anxiety/depression* domains which, in the affirmative, could be further assessed by administering specialized scales.

### 3.5. Use Case Examples for coQoL

The coQoL method allows for the in-depth analysis of the results both in terms of measured outcomes and individual participants. We provide two examples below, pertaining to longitudinal data (Section 3.5.1) and the story of a participant (Section 3.5.2).

#### 3.5.1. Longitudinal Data Example

We exemplify a very strong correlation ($r_{S}$ = 0.9) between PROs and TechROs, to report how the interval and leeway durations influenced the correlations. In healthy participants, the MSPSS item Q3 (*family is trying to help*, PRO) correlated the strongest with the Fitbit fair physical activity duration in the CLR PA+S family, TechRO) for the TechRO aggregation interval of 28 days with a decreasing pattern as the leeway increases. Table 15 presents the resulting gradients of correlations for all combinations of TechRO aggregation interval-leeway durations and the TechRO raw data that yielded the strongest correlation. Table 16 depicts the raw results. In this table, the relative *fair* column is a centred log-ratio that has both negative (for less relative *fair* activity) and positive quantities (for more relative *fair* activity).

#### 3.5.2. Participant Story Example

Participant 169 is a 69-year-old female from Hungary who self-reported mild disease. She has a university degree, lives with her partner (no children), does not smoke, and drinks alcohol daily. She is a diligent responder who answered in all three waves of our study, wore the Fitbit for 794 days from which 141 were valid.

When aligning the numeric scores from the PRO scales and the TechROs (Table 17), Wave 1 (mid-2018) had the worst PRO depression and anxiety, (close to the worst) memory, and sleep as well as (close to) the worst TechRO sedentary duration, light activity duration, (close) fair activity, and vigorous activity duration. Wave 2 (end-2018 and start-2019) had the least adequate PRO physical activity, adherence to the Mediterranean diet, memory, sleep, and quality of life, reflected in the least adequate TechRO energy expenditure, steps, heart rate, sedentary duration, fair activity duration, and total active duration per day. In Wave 3 (mid-2019), Participant 169 registered better PRO for physical activity, depression and anxiety, memory, and sleep as well as more steps, a shorter sedentary duration, and longer light, fair, and vigorous durations. Social support was always high but never optimal. Nutrition and Quality of Life maintained high, but not optimal for waves 1 and 3. During the winter, the sleep duration was higher than during the summer. This real user example illustrates and emphasizes the importance of longitudinal state and behaviour assessments; we observed the change of state in participant 169 as a change in the TechRO variables that indeed associated with worse PRO-based self-reported states.

## 4. Discussion

In this section we discuss our methodological approach (Section 4.1), the coQoL method in the perspective of past evidence (Section 4.2), observations on data quality (Section 4.3), and pathways towards personalized medicine (Section 4.4). We then review several limitations of our study (Section 4.5) and envision future work (Section 4.6).

### 4.1. Overall Methodological Approach in PROomics

The coQoL method explored patterns of correlations between PROs and TechROs towards their co-calibration. Consequently, we focused on identifying groups of strong correlations between PROs with a given recall period and TechROs, aggregating weeks to months of wearables data available before the administration day of the PRO. We considered correlations between similar latent constructs, e.g., PRO and TechRO physical activity or sleep, as high from 0.8 and above. However, for different latent constructs, such as PRO social support and TechRO sleep, where the probability of random correlation is low, correlations of even 0.5 are high. Hence, we presented in here correlations of 0.5 and above as of importance.

Due to the exploratory nature of our method, we deliberately omitted adjustments for multiple comparisons. The results of our method can guide future observational studies, as well as personalized, adaptive interventional studies, where the observational component will inform the intervention design *as we go*. Researchers can power such studies for enough confidence to exclude trivial effects.

### 4.2. coQoL in Perspective of Past Evidence

We recall that little prior research focused on assessing the relationships between sets of different outcomes assessed via PROs and consumer wearable TechROs in healthy seniors, in the wild, for extended periods (beyond the typical study duration of 7–14 days). On the one hand, past studies may have had similar to larger sample size, yet they have not yielded stronger statistical results; these co-calibrations rarely report values $r_{S}$ ≥ 0.5, as we do. On the other hand, we report a more prolonged study duration (up to 2 years). The study duration of over a few weeks is essential to overcome the “novelty” effect of the technology (TechRO) on the state and behaviour of the user. Namely, the user, motivated by the feedback provided by the device while the study is being conducted, may move more or sleep differently, which then would be erroneously co-calibrated with the self-reports (PROs). The coQoL method leads to more accurate, real-world PRO- and TechRO-based datasets representing the real states and behaviours of the users. We define the past evidence in the context of *momentary co-calibration* efforts, where the PRO-TechRO co-calibrations may have been valid only for the short interval of data collection. Our proposed method coQoL expands the state of the art.

### 4.3. Observations on Data Quality

The wearable monitored some TechROs for more days than others. For example, the energy expenditure and steps appeared in most days. However, some days did not include durations of physical activity at increasing intensities, due to some seniors not wearing the wearable for enough hours that Fitbit recognized the activity or they did not reach the increased intensity physical activity on those days. Also, the TechROs that combine other TechROs, e.g., *fair+vigorous*, appeared in at most the minimum of the numbers of days when their constituent TechROs appeared. We acknowledge errors of a few days in long-term monitoring stemming from conditions beyond our control, such as errors at the device setup, at the recruitment site which took days to correct, or when running the automated data collectors from the seniors that were beyond our control in the project. These technological and human factors influenced the quality of the available data.

The wearable monitoring period may depend on the measured outcome, frequency of answers, and human factors. While the recall period of many scales is short (e.g., one week), collecting wearable data only for that duration may prove too strict. If the design is too strict, numerous participants will disqualify, and the results may bias in favour of diligent or adherent responders, who may also exhibit positive behaviours, e.g., exercising more diligently as well. Although some results indicate that 14–28 days of data could be enough for significant co-calibrations, the observations used in the co-calibration depend on the PRO answers and the TechRO data alike. If the participants are adherent to data collection for four weeks, but do not answer the questionnaire, the quality of the data may be insufficient to derive correlations. For some questionnaires, coQoL may relax the alignment (*leeway*) to account for human factors that contributed to data loss. On the other hand, a monitoring window of 120 days (4 months) may prove too wide to collect data reflecting the same behaviour as the reported one (the recall period), also because of the potential influence of seasonal effects. These seasonal, as well as other context dependencies, are illustrated when applying the coQoL to the MSPSS social support PRO. Our results indicate that having approximately one month of data before the administration of the MSPSS is sufficient to obtain significant correlations between *family trying to help* social support and *fair* activity even within a small sample of 39 participants. We observe that the MSPSS is time context-specific. Overall, across all questionnaires, we argue for an intermediary period of aggregation interval for TechRO not extending beyond 60–90 days.

### 4.4. Pathways towards Personalized Medicine

There is growing evidence within the medical domain that personal data paves a path towards personalized medicine, including genetics data and population-specific data, as well as, on a growing scale, data originating in the individuals’ daily life environments and representing their natural, objective behaviours unfolding in different contexts of daily life. Daily life datasets are, in turn, collected via consumer wearables and smartphones with sensing capabilities.

From our study, we learn that an ideal wearable in the context of personalized medicine study would be comfortable to wear; should have a long battery life (at least a few days); should be accepted by individuals to use as their own, such that they forget they are in the study (implying minimal reactivity); and should provide relevant TechRO related to behavioural patterns (e.g., activity status, steps, as opposed to only heart rate, which would be hard to co-calibrate by itself).

Given our results, we also observe that for some PROs, different self-reported health status of the individuals yield different co-calibration results, even though our definition of disease refers only to mild self-reported cases. When the participants have a disease, other TechROs become correlated more strongly with other PROs than for the healthy ones. An observational study involving healthy individuals can leverage the coQoL method by monitoring a relevant subset of PRO/TechROs longitudinally, and occasionally co-calibrating the PROs with TechROs assuming the sensitivity of the coQoL method for when long-term, significant changes in TechRO occur. Based on the occasionally collected PRO answers, further in-depth examination of the individual’s state may seek to understand if the TechRO change signals coincide with a significant and relevant PRO change, potentially implying a real change of the individual’s health state. Once diagnosed, the individual’s health state may be followed up, assuming another set of PRO/TechRO outcomes co-calibrated in time, to assess the change in the state of the disease accurately.

For example, in the case of diseased Participant 169, we observed that improvements or deteriorations in the state (as self-reported via the PROs for physical activity, Mediterranean diet, memory, and Quality of Life) coincided with TechROs (of physical activity in the sedentary, and light-vigorous spectrum, as well as the total physically active duration). Such trends are likely to differ between persons. As observed with Participant 169, administering the PROs only three times in two years and monitoring the TechRO behaviours using the wearable (minimally obtrusively, continuously, during daily life) yielded numerous trends across not only pairs of PROs and TechROs, but also across different PROs and TechROs.

The coQoL can provide a frontline approach to further triage the individual state assessment, for the healthy or diseased, without burdening the individuals with self-assessments, and at the same time without excluding participants who develop diseases and need to be monitored for long periods. In the context of the latter, the coQoL may be very suitable to assess changes of behaviour and health state in chronically ill patients.

We envision the following coQoL use case. The coQoL results can inform the design of longitudinal observations for selected individual PRO/TechRO outcomes, leveraged in personalized medicine solutions. The procedure consists of the observation for several consecutive days (for more TechRO-adherent participants, four weeks; for the less adherent participants, up to 3 months, from which one can derive around four weeks of quality data) followed by the co-calibration of TechROs with PROs. While monitoring, a potential gradual change in a subset of TechROs of interest can lead to contacting the individual for further health outcome assessments, via PRO or even clinical examination.

In new study designs, we suggest the study participation period of 60–90 days at most, and leverage behavioural techniques for participant wearable-adherence, to maximize the validity of the results acquired. The study design may imply repeated measures longitudinally over the years, e.g., PRO/TechRO co-calibration efforts over 60–90 consecutive days, repeated every few months up to a year (assuming same season every year).

### 4.5. Study Limitations

Several limitations characterize the presented here preliminary coQoL study. The first limitation is the small sample size, specific to an exploratory feasibility study. A second limitation is the resulting lack of power that reduced the complexity of the analysis method (i.e., statistical hypothesis tests). A third limitation is the presence of multiple PRO answers per individual for the same wave, albeit with high variability. However, we only included one answer per participant-wave to reduce bias towards diligent responders. In case of multiple answers per participant-wave, we chose the latest answer in time, to account for any form submission issues in the CoME software application or the participant changing their mind after submitting the answers once. A fourth limitation is a significant decrease in the number of participants data leveraged for the co-calibrations; we allowed for a *leeway* to allow PRO and TechRO alignments that are both (1) short-term, but accurate (e.g., 7–14 days, close to the recall period), and (2) longitudinal, but permissive (e.g., 60–120 days, sufficient for the long-term behaviours to unfold). The study highlights the challenge of retaining individuals (shared by many health studies) that can provide outcomes through both self-report and a wearable that must be worn daily, over long periods.

### 4.6. Future Work

In the ongoing and future work, we expect to involve more participants for shorter periods (60–90 days), repeated every few months to a year, and focus on the PROs and TechROs delineated in this paper to deepen our knowledge about these specific co-calibration efforts and results. We plan to employ more advanced techniques and obtain more results within statistical significance as we increase the sample size in further studies aimed at calibrating PROs and TechROs for health outcomes and longitudinal behaviours such as physical activity and sleep in seniors. We aim to derive individual co-calibration trajectories models, as well as population models, e.g., similar groups of healthy or diseased individuals.

## 5. Conclusions

In this study, we present the coQoL method for co-calibrating the relationships between PROs and TechRO for eight PRO outcomes and TechRO behavioural markers of physical activity, sleep, and heart rate in a cohort of 42 seniors contributing data for two years. We reported human factors and quality properties from the data collected while their daily life unfolded. Our results can inform the design of personalized observational that assess daily life behaviours continuously and longitudinally, and that enable interventional studies towards reducing the risk of chronic disease and improve health and Quality of Life in the long term.

## Figures and Tables

**Table 1:** Previous PRO-TechRO Co-Calibration Studies.

Outcome	PRO: Name	TechRO: Name, Position on Body	Study: Population, Sample, Duration	Co-Calibration: Method	Results	Reference
Physical Activity	International Physical Activity Questionnaire (IPAQ); Physical Activity for Adults Questionnaire (PAAQ)	Actical (research-grade accelerometer), right hip	Individuals, N=112, age range 18–79, mean age 47, 7 days, in the wild	Spearman correlation	PAAQ and IPAQ agreed for moderate and vigorous activity ($r_{S}$ = 0.44, $r_{S}$ = 0.2, respectively).	Garriguet et al. (2015) [36]
Physical Activity	International Physical Activity Questionnaire (IPAQ)	Fitbit (consumer wearable), non-dominant arm; ActiGraph GT3X+ (research-grade accelerometer), right waist	Students, N=53, mean age 28.10 ± 9.12, 7 days	Paired t-test, Bland Altman	No significant correlations were found between the IPAQ and the two devices.	Brewer et al. (2017) [37]
Physical Activity	Godin Leisure-Time Exercise Questionnaire (GLTEQ)	Fitbit Alta (consumer wearable), wrist	Endometrial cancer survivors, N=25, mean age 62 ± 9, 30 days	U statistic	No significant correlations were found between the GLTEQ and steps.	Rossi et al. (2018) [38]
Physical Activity	International Physical Activity Questionnaire (IPAQ)	Fitbit Zip (consumer wearable), wrist; ActiGraph GTX3 (research-grade accelerometer)	Seniors, N=70, age range 62–77, mean age 70.1 ± 3.3), 7 days (ActiGraph, Fitbit), 70 days (study)	Descriptive	IPAQ good for duration of activities but not intensity.	Meyer et al. (2019) [39]
Physical Activity	International Physical Activity Questionnaire (IPAQ), Patient Health Questionnaire (PHQ)	Fitbit Charge 3 (consumer wearable), wrist	Individuals with depression, N=8, age range 18–95, mean age 45, 8 weeks	Descriptive	IPAQ score associated with Fitbit steps.	Santomas et al. (2020) [40]
Social Support	Pittsburgh Sleep Quality Index (PSQI), Pittsburgh Sleep Diary (PghSD), Interpersonal Support Evaluation List (ISEL), Hamilton Rating Scale for Depression (HRSD), Comorbidity Questionnaire, and others	Actiwatch 64 (accelerometer), wrist	Individuals with and without chronic insomnia, N=119 (79 with insomnia), min. age 60, 7 days	Analysis of covariance (ANCOVA), ordinal logistic regression	Social support associated with lower wakefulness after sleep onset for all participants, and shorter sleep latency for those with insomnia.	Troxel et al. (2010) [41]
Social Support	Social Support Scale for Exercise Behaviour and others	ActiGraph (accelerometer)	Seniors, N=718, mean age 74.4 ± 6.3, 7 days	Mixed effects regression	Socially supportive environment related to 30 min. to 1 h. of physical activity in participants with positive psycho-social attributes and up to 30 min. for those with less positive psycho-social attributes.	Carlson et al. (2012) [42]
Social support	Hospital Anxiety and Depression Score (HADS), Short Form Health Survey (SF-36)	RT3 (accelerometer), waist	Seniors, N=547, mean age 79 ± 8, 7 days	Multiple regression	Number of people nearby to turn to associated with higher physical activity ($R_{2}$ = 0.32).	McMurdo et al. (2012) [43]
Social support	Custom questionnaire to estimate social networks and social engagement, Center for Epidemiological Studies Depression (CES-D), Montreal Cognitive Assessment (MoCA), and others	Actiwatch Spectrum (accelerometer), non-dominant wrist	Seniors, N=673, mean age 71.9 ± 7.2, 3 days	Multivariate linear regression	Larger social networks (p=0.04), higher network proportion of friends (p=0.01), more frequent visiting with neighbors (*p* < 0.01), and more frequent attendance at organized group meetings (p=0.03) associated with higher physical activity intensity levels.	Ho et al. (2018) [44]
Social support	Iowa-Netherlands Comparison Orientation Measure, Rochester Social Comparison Record, and others	Fitbit Flex (consumer wearable), wrist	College women, N=80, mean age 20 ± 1.07, 7 days	Multilevel regression	Increase in negative social interactions (especially with friends) were consistently associated with decreases in daily physical activity with high variability.	Arigo et al. (2019) [45]
Social support	University of California Los Angeles Loneliness Questionnaire	Fitbit Flex 2 (consumer wearable), wrist	First-year college students, N=160, 16 weeks (one semester)	Data mining (Apriori), machine learning classification (gradient boosting, logistic regression)	Binary level of loneliness can be detected with 80.2% accuracy. More physical activity and less sedentary behaviour associated with less loneliness.	Doryab et al. (2019) [46]
Anxiety and Depression	Patient Health Questionnaire (PHQ-9), Generalised Anxiety Disorder 7-Item Scale (GAD-7), International Physical Activity Questionnaire (IPAQ), Social Support, and others	SenseWear (accelerometer), arm	Individuals with chronic major depressive disorder or a bipolar 2 disorder, N=14, age range 42–72, mean age 54.5 ± 8.7, 7 days (wear), 14 weeks (study)	Wilcoxon signed rank difference test	Physical activity results in an improvement in anxiety and depression in patients with chronic depression (median depression score decreased 38%, *p* < 0.05).	Adams et al., 2015 [47]
Anxiety and Depression	Patient Health Questionnaire (PHQ-9), Mini International Neuropsychiatric Interview (MINI), Montgomery-ÅDepression Rating Scale (MADRS)	ActiGraph GT3X+ (accelerometer)	Anxiety and depression patients, N=165, age range 18–65, mean age 41.8 ± 11.6, 7 days	Analysis of variance (ANOVA), analysis of covariance (ANCOVA), paired t-tests	No significant results; depressed participants tended to be less active at light intensity (β = −2.21, *p* < 0.01).	Helgadóttir et al. (2015) [48]
Anxiety and Depression	Depression Anxiety Stress Scale (DASS)	Fitbit (consumer wearable), wrist	University students and staff, N=85, mean age 22 ± 3, 3 weeks	Analysis of variance (ANOVA)	An increase in steps correlated with a decrease in depression for female participants.	Liau et al. (2018) [49]
Anxiety and Depression	University of California Los Angeles Life Stress Interview (LSI), Generalized Anxiety Disorder 7-Item Scale (GAD-7), Patient Health Questionnaire (PHQ-9)	Fitbit Charge 2 (consumer wearable), wrist	Female adolescents, *N* = 30, mean age 16.4 ± 0.8, 1 year, mean wear 7 months	Pearson correlation, Bayesian multilevel models	Within-person fluctuations in stressful life events were associated with variability in sleep duration (*r* = 0.48, *p* < 0.05). Within-person increases in sleep duration variability correlated with greater depression symptoms ($r_{S}$ = 0.38, *p* < 0.05) while sleep regularity correlated with lesser depression ($r_{S}=-0.44$, *p* < 0.05).	Vidal Bustamante et al. (2020) [50]
Memory	Montreal Cognitive Assessment (MoCA), Alzheimer Disease Assessment Scale-Cognitive-Plus (ADAS-Cog Plus)	MotionWatch 8 (accelerometer), wrist	N = 151, min. age 55, mean age 71.1 ± 7.2, 5 days	Paired t-test, analysis of covariance (ANCOVA), multiple linear regression	Participants with probable mild cognitive impairment were less active and more sedentary, better ADAS-Cog Plus performance correlates with more physical activity and less sedentary behavior.	Falck et al. (2017) [51]
Memory	Self-reported learning experience (satisfaction, usefulness, and performance)	Empatica E4 (accelerometer), non-dominant wrist	College students, N=31, age range 21–53, mean age 24 ± 5.9, 35 min	Machine learning (random forest, support vector machine with 3 separate kernels)	Students’ perceived learning can be predicted accurately from the physiological data (89% accuracy).	Giannakos et al. (2020) [52]
Memory	Enroll-HD cognitive battery	Fitbit (consumer wearable)	Individuals with Huntington’s disease, N=70 (20 healthy controls), 3 uses across 8 days	Correlation tests	Medium to strong correlations between motor symptoms and cognitive tasks (r = −0.34–0.54).	McLaren et al. (2020) [53]
Sleep	Pittsburgh Sleep Quality Index (PSQI), Perceived Stress Scale (PSS), Short Form Health Survey (SF-12)	Q-sensor (accelerometer), dominant hand	Undergraduate students, N=66, mean age 20.1 ± 1.5, 30 days	Machine learning (classification, support vector machine with 2 separate kernels)	Skin conductance, skin temperature, and acceleration classified poor/good sleep with 80–90% accuracy.	Sano et al. (2015) [54]
Sleep	Pittsburgh Sleep Quality Index (PSQI), Charlotte Attitudes Towards Sleep Scale (CATS), Sleep Hygiene Practice Scale (SHPS), and others	Fitbit Flex (consumer wearable), wrist	College students, N=218, age range 18–38, mean age 20.3 ± 2.5, 7 days	Path model, Spearman correlation	Correlations between sleep duration from PSQI and Fitbit ($r_{S}$ = 0.33, *p* < 0.01).	Peach et al. (2018) [55]
Sleep	Pittsburgh Sleep Quality Index (PSQI)	Fitbit Flex 2 (consumer wearable), wrist	Military individuals, N=17, 2 weeks	Wilcoxon signed rank difference test, Spearman rank correlation test	Moderate correlation between PSQI and Fitbit sleep durations ($r_{S}$ = 0.643, p = 0.005). Top contextual factors disrupting sleep were pain, noises, and worrying.	Thota et al. (2020) [56]
Quality of Life	Self-reported health scale (5 levels)	ActiGraph GT1M (accelerometer)	Seniors, N=560, age range 65–85, mean age 71.6 ± 5.6, 7 days	Analysis of variance (ANOVA)	51% higher physical activity level was registered in those with very good health compared to those with poor and very poor health.	Lohne-Seiler et al. (2014) [57]
Quality of Life	Short Form Health Survey (SF-12), Oswestry Disability Index (ODI)	Fitbit Zip (consumer wearable)	Lumbar spine surgery patients, N=30, mean age 42.6 ± 10.3, 7 days (pre-operatory wear), 6 months (post-operatory wear)	Paired t-test, Pearson correlation	No significant correlation between the improvement in steps (p>0.2) or distance traveled per day (p>0.3).	Mobbs et al. (2015) [58]
Quality of Life	Eastern Cooperative Oncology Group Performance Status (ECOG-PS), Karnofsky Performance Status (KPS), Patient-Reported Outcomes Measurement Information System (NIH PROMIS)	Fitbit Charge HR (consumer wearable), wrist	Advanced cancer patients, N=37, age range 34–81, median age 62, 2 weeks	Spearman correlation, Kaplan-Meier curves, multivariate proportional hazards	Correlations were observed between average daily steps and ECOG-PS ($r_{S}=-0.63$, *p* < 0.05) and KPS ($r_{S}$ = 0.69). Correlations were also observed between distance and ECOG-PS ($r_{S}=-0.61$) and KPS ($r_{S}=0.66$).	Gresham et al. (2018) [59]
Quality of Life	EuroQoL with 5 Dimensions and 3 Levels (EQ-5D-3L)	Fitbit One (consumer wearable), belt	Stroke patients, N=27, mean age 69.5, 7 days	Correlation tests	Quality of Life health score correlates with the number of steps (r = 0.46, *p* < 0.03).	Sasaki et al. (2018) [60]
Quality of Life	Short Form Health Survey (SF-12), Knee Injury and Osteoarthritis Outcome Score (KOOS)	Fitbit Flex (consumer wearable), non-dominant wrist	Knee arthroplasty patients, N=91, mean age 67 ± 13, 7 days for 3 times points (2 weeks before surgery, day after surgery, and 2 weeks after surgery)	Multiple linear regression, Spearman rank correlation	Significant correlations of SF-12 (physical component summary) and post-operative step count ($r_{S}$ = 0.521, *p* < 0.05).	Twiggs et al. (2018) [61]

The magenta font color highlights important limitations to the existing studies.

**Table 2:** Variables derived from the PROs.

Outcome	Scale	Item Variables	Score Variables	Total
Physical Activity	International Physical Activity Questionnaire (IPAQ) [26]	15: 11 for the combinations of domains and intensities, 4 for the domain totals	8: 4 for the domain numeric scores, 3 for the intensity numeric scores, and 1 for the overall numeric score	23
Social Support	Multi-Dimensional Scale Perceived Social Support (MSPSS) [27]	12 for the items	5: 3 for the numeric sub-scores and 2 for the numeric and categorical scores	17
Anxiety and Depression	Goldberg depression and anxiety scale (GADS) [28]	18 for the items	2 for the numeric and categorical scores	20
Nutrition Mediterranean	Prevention with Mediterranean Diet (PREDIMED) [29,30]	14 for the items	2 for the numeric and categorical scores	16
Nutrition	Self-Reported Mini Nutritional Assessment (SelfMNA) [31]	5 for the items	2 for the numeric and categorical scores	7
Memory	Memory Failures of Everyday (MFE) [32]	28 for the items	2 for the numeric and categorical scores	30
Sleep	Pittsburgh Sleep Quality Index (PSQI) [33]	18 for the items	10: 8 for the sub-scores and 2 for the numeric and categorical scores	28
Health-Related Quality of Life	EuroQoL health questionnaire (EQ-5D-3L) [34]	6 for the items	0 (the scores coincide with the items)	6

**Table 3:** Variables derived from the TechROs.

Amount	Family	Outcome	Variable	Unit
Absolute	Raw	Energy	Median count over 7 days	kcal.
			Median count over 14 days	
			Median count over 21 days	
			Median count over 28 days	
			Median count over 60 days	
			Median count over 90 days	
			Median count over 120 days	
		Steps	Median count over [...] days	count
		Heart rate	Median beats over [...] days	bpm.
	Processed	Sedentary	Median duration over [...] days	min.
		Sedentary+Light	Median duration over [...] days	
		Light	Median duration over [...] days	
		Light+Fair	Median duration over [...] days	
		Fair	Median duration over [...] days	
		Fair+Vigorous	Median duration over [...] days	
		Vigorous	Median duration over [...] days	
		Active	Median duration over [...] days	
		Sleep	Median duration over [...] days	
Relative	CLR PA	Sedentary	Geometric mean over [...] days	-
		Light	Geometric mean over [...] days	
		Fair	Geometric mean over [...] days	
		Vigorous	Geometric mean over [...] days	
	CLR PA+S	Sedentary	Geometric mean over [...] days	
		Light	Geometric mean over [...] days	
		Fair	Geometric mean over [...] days	
		Vigorous	Geometric mean over [...] days	
		Sleep	Geometric mean over [...] days	

**Table 4:** Characteristics of Study Participants.

Variables	Mean (SD) or n [%]		Variables	Mean (SD) or n [%]	
	Spain	Hungary		Spain	Hungary
Count	26 [66.7%]	13 [33.3%]	Health status		
Age	69.2 (±5.7)	71.5 (±9.1)	Healthy	18 [46.2%]	10 [25.6%]
Gender			Diseased	8 [20.5%]	3 [7.7%]
Women	15 [38.5%]	7 [17.9%]	Smoking		
Men	11 [28.2%]	6 [15.4%]	Yes	5 [12.8%]	1 [2.6%]
Education			No	21 [53.8%]	12 [30.8%]
Primary	7 [17.9%]	0 [0.0%]	Alcohol		
Secondary	5 [12.8%]	3 [7.7%]	Never	10 [25.6%]	4 [10.3%]
High school	5 [12.8%]	1 [2.6%]	Monthly	5 [12.8%]	5 [12.8%]
University	9 [23.1%]	9 [23.1%]	Weekly	7 [17.9%]	1 [2.6%]
Living			Few days	1 [2.6%]	2 [5.1%]
Alone	11 [28.2%]	3 [7.7%]	Daily	3 [7.7%]	1 [2.6%]
+Partner	14 [35.9%]	10 [25.6%]	Systolic blood pressure	146.2 (±63.2)	124.7 (±15.0)
+Children	1 [2.6%]	0 [0.0%]	Body mass index	25.5 (±4.64)	28.5 (±4.1)

+: addition to the previous row.

**Table 5:** PRO count answers by wave and questionnaire (N=39 participants).

							Wave 1									Wave 2									Wave 3							
	PID	Health	Country	Gender	Age		Physical Activity (IPAQ)	Social Support (MSPSS)	Anxiety and Depression (GADS)	Mediterranean Nutrition (PREDIMED)	Nutrition (SelfMNA)	Memory (MFW)	Sleep (PSQI)	Quality of Life (EQ-5D-3L)		Physical Activity (IPAQ)	Social Support (MSPSS)	Anxiety and Depression (GADS)	Mediterranean Nutrition (PREDIMED)	Nutrition (SelfMNA)	Memory (MFW)	Sleep (PSQI)	Quality of Life (EQ-5D-3L)		Physical Activity (IPAQ)	Social Support (MSPSS)	Anxiety and Depression (GADS)	Mediterranean Nutrition (PREDIMED)	Nutrition (SelfMNA)	Memory (MFE)	Sleep (PSQI)	Quality of Life (EQ-5D-3L)
	575	Healthy	Hungary	Female	65												•	•	•		•	•	•									
	569	Healthy	Hungary	Female	67			•	•			•		•		•	•						•			•	•	•	•	•	•	•
	133	Healthy	Hungary	Female	71			•	•		•	•	•	•		•	•						•		•	•	•	•		•	•	•
	420	Healthy	Hungary	Female	71		•	•						•		•	•	•	•	•	•	•	•									
	215	Healthy	Hungary	Female	87		•	•	•	•	•	•	•	•																		
	576	Healthy	Hungary	Male	60											•	•						•									
	535	Healthy	Hungary	Male	69																				•	•	•	•	•	•	•	•
	170	Healthy	Hungary	Male	70											•	•						•		•	•	•	•		•	•	•
	212	Healthy	Hungary	Male	72		•	•						•		•	•						•			•						•
	419	Healthy	Hungary	Male	95			•						•		•	•	•	•	•	•	•	•									
	643	Healthy	Spain	Female	67			•	•		•	•	•	•		•	•	•	•		•	•	•									
	798	Healthy	Spain	Female	67																					•						•
	803	Healthy	Spain	Female	67																										•	•
	617	Healthy	Spain	Female	69			•						•		•	•	•	•	•	•	•	•									
	620	Healthy	Spain	Female	69											•	•	•	•	•	•	•	•									
	640	Healthy	Spain	Female	69			•	•		•	•	•	•																		
	628	Healthy	Spain	Female	70			•	•		•	•	•	•																		
	638	Healthy	Spain	Female	71			•	•		•	•	•	•		•	•						•									
	648	Healthy	Spain	Female	72			•	•	•	•	•	•	•																		
	649	Healthy	Spain	Female	72							•		•																		
	795	Healthy	Spain	Female	72																				•	•	•	•	•	•	•	•
	630	Healthy	Spain	Female	74			•	•		•	•	•	•																		
	411	Healthy	Spain	Male	45			•						•																		
	790	Healthy	Spain	Male	66																				•	•	•	•	•	•	•	•
	700	Healthy	Spain	Male	67												•	•	•		•	•	•									
	636	Healthy	Spain	Male	68			•						•		•	•						•									
	793	Healthy	Spain	Male	68																				•	•						•
	796	Healthy	Spain	Male	74																				•	•	•	•	•	•		•
	502	Diseased	Hungary	Female	63																											•
	169	Diseased	Hungary	Female	69			•	•	•	•	•	•	•		•	•	•	•		•	•	•		•	•	•	•	•	•	•	•
	132	Diseased	Hungary	Male	71			•	•			•		•			•				•		•			•	•	•		•	•	•
	800	Diseased	Spain	Female	65																					•	•	•	•	•	•	•
	641	Diseased	Spain	Female	71			•	•		•	•		•		•	•	•	•		•	•	•									
	624	Diseased	Spain	Female	72			•	•		•	•	•	•																		
	644	Diseased	Spain	Male	70			•						•																		
	625	Diseased	Spain	Male	72			•	•		•	•	•	•																		
	634	Diseased	Spain	Male	72			•					•	•		•	•						•									
	791	Diseased	Spain	Male	72																				•	•						•
	799	Diseased	Spain	Male	79																					•	•	•	•	•	•	•

Color coding: from orange (fewer scales answered in a wave) to yellow to green (more answered).

**Table 6:** PROs with high total count of significant Spearman correlations ($r_{S}$ ≥ 0.5) with TechROs.

Outcome	PRO	Health	PRO	TechRO Families				
			Item/Sub-Score/Score	Raw	Processed	CLR PA	CLR PA+S	All
Physical activity	IPAQ	All	Domestic moderate activity		4	2	2	8
Physical activity	IPAQ	All	Domestic+garden total activiy		3	2	3	8
Physical activity	IPAQ	All	Garden moderate activity		4	2	1	7
Physical activity	IPAQ	All	Leisure moderate activity	1	3	2	1	7
Physical activity	IPAQ	Healthy	Domestic moderate activity	2	4	3	2	11
Physical activity	IPAQ	Healthy	Garden moderate activity		6	4		10
Physical activity	IPAQ	Diseased	Garden vigorous activity	1	6	3	2	12
Physical activity	IPAQ	Diseased	Leisure vigorous activity	2	6	2	2	12
Physical activity	IPAQ	Diseased	Work vigorous activity	1	5	3	2	11
Physical activity	IPAQ	Diseased	Work moderate activity	2	5	1	2	10
Social support	MSPSS	All	Q8: family talks about problems		4	3	3	10
Social support	MSPSS	All	Q11: family willing to help make decisions	1	5	2	2	10
Social support	MSPSS	Healthy	Q3: family tries to help	1	6	3	4	14
Social support	MSPSS	Healthy	Q6: friends try to help	1	7	2	4	14
Social support	MSPSS	Healthy	Q9: friends share joys and sorrows	1	6	2	4	13
Social support	MSPSS	Healthy	Q12: friends talk problems	1	7	2	3	13
Social support	MSPSS	Healthy	Q10: special person cares about feelings		7	1	4	12
Social support	MSPSS	Healthy	Friends numeric sub-score	1	6	2	3	12
Social support	MSPSS	Diseased	Q2: special person shares joys and sorrows	1	5			6
Social support	MSPSS	Diseased	Significant other numeric sub-score	1	4		1	6
Anxiety and depression	GADS	All	Q6D: lost weight due to poor appetite		5	3	4	12
Anxiety and depression	GADS	All	Q8A: worried about own health		4	4	2	10
Anxiety and depression	GADS	All	Q1D: lacking energy		3	3	4	10
Anxiety and depression	GADS	Healthy	Q2D: lost interest in things		6	3	3	12
Anxiety and depression	GADS	Diseased	Q2A: worrying a lot	2	6	2	1	11
Mediterranean nutrition	PREDIMED	All	Categorical score	2	4	3	1	10
Mediterranean nutrition	PREDIMED	All	Numeric score	1	3	4	1	9
Mediterranean nutrition	PREDIMED	All	Q12: nuts use	2	2	1	2	7
Mediterranean nutrition	PREDIMED	All	Q14: sofrito use		2		5	7
Mediterranean nutrition	PREDIMED	Healthy	Q4: fruit use	1	3	2	1	7
Mediterranean nutrition	PREDIMED	Healthy	Categorical score		2	2	2	6
Nutrition	SelfMNA	All	Categorical score		2	2	2	6
Nutrition	SelfMNA	Healthy	Categorical score		1	2	2	5
Nutrition	SelfMNA	Diseased	Q2: weight lost	1	3	1	2	7
Nutrition	SelfMNA	Diseased	Q1: food intake declined	1	2	1	2	6
Memory	MFE	All	Q12: having difficulty picking up a new skill		6	1	4	11
Memory	MFE	All	Q14: forgetting to do planned things		5	2	3	10
Memory	MFE	All	Q6: forgetting time of events		4	3	2	9
Memory	MFE	Healthy	Q6: forgetting time of events	1	7	3	3	14
Memory	MFE	Healthy	Q15: forgetting details of done things		7	2	4	13
Memory	MFE	Healthy	Q12: having difficulty picking up a new skill		6	3	3	12
Memory	MFE	Healthy	Q14: forgetting to do planned things	1	6	2	3	12
Memory	MFE	Diseased	Q13: having a word on the tip of the tongue	1	7	3	2	13
Memory	MFE	Diseased	Q25: getting lost in often visited place		7	3	2	12
Sleep	PSQI	All	Q7: trouble staying awake driving, eating, socializing	2	5	4	3	14
Sleep	PSQI	All	Q4: duration of actual sleep	1	5	3	2	11
Sleep	PSQI	All	Daily dysfunction numeric sub-score	1	4	3	2	10
Sleep	PSQI	Healthy	Q4: duration of actual sleep	1	5	3	2	11
Sleep	PSQI	Healthy	Q5C: trouble sleeping due to using the bathroom		4	4	2	10
Sleep	PSQI	Healthy	Q7: trouble staying awake driving, eating, socializing	2	5	3		10
Sleep	PSQI	Healthy	Daily dysfunction numeric sub-score	2	3	3	1	9
Sleep	PSQI	Diseased	Daily dysfunction numeric sub-score	2	4	1		7
Sleep	PSQI	Diseased	Q6: duration of actual sleep		4	2		6
Quality of Life	EQ-5D-3L	All	Q6: health state today		4	1	3	8
Quality of Life	EQ-5D-3L	All	Q4: pain/discomfort		2	1	3	6
Quality of Life	EQ-5D-3L	Healthy	Q4: pain/discomfort		4	2	1	7
Quality of Life	EQ-5D-3L	Diseased	Q5: anxiety/depression	2	3			5


Color coding: from orange (less correlations) to green (more correlations).

**Table 7:** Summary of strong and significant Spearman rank correlations ($r_{S}$ ≥ 0.8) between PROs of physical activity (IPAQ scale) and TechROs (Fitbit wearable).

	PRO		TechRO			Correlation/Contour				
Health	Domain	Variable	Amount	Family	Variable	Lower		$r_{S}$	Higher	
All	Work	Vigorous activity	Absolute	Processed	Active			+0.8		
All	Domestic	Moderate activity	Absolute	Processed	Light+fair		+0.7	+0.8	×	
Healthy	Work	Moderate activity	Absolute	Processed	Fair		×	−0.8	×	
Healthy	Work	Total activity	Absolute	Processed	Fair		×	−0.8	×	
Diseased	Work	Walking activity	Relative	CLR PA	Light		−0.7	−0.8	×	
Diseased	Work	Moderate activity	Absolute	Processed	Fair		×	+0.8	+0.7	+0.7
Diseased	Work	Moderate activity	Relative	CLR PA	Sedentary			−0.8	×	
Diseased	Work	Vigorous activity	Relative	CLR PA	Light		−0.7	−0.8	−0.6	
Diseased	Garden	Vigorous activity	Relative	CLR PA	Light		−0.7	−0.8	−0.5	
Diseased	Garden	Vigorous activity	Relative	CLR PA+S	Sedentary			−0.8	−0.7	
Diseased	Leisure	Walking activity	Absolute	Raw	Energy			+0.8		
Diseased	Leisure	Walking activity	Absolute	Raw	Steps			+0.8		
Diseased	Leisure	Vigorous activity	Absolute	Processed	Fair+Vigorous		×	+0.8	+0.6	
Diseased	Leisure	Vigorous activity	Relative	CLR PA	Sedentary			−0.8	×	
Diseased	Leisure	Vigorous activity	Relative	CLR PA	Vigorous		×	+0.8		
Diseased	Leisure	Vigorous activity	Relative	CLR PA+S	Light		−0.7	−0.8	×	
Diseased	Leisure	Total activity	Absolute	Processed	Sedentary+light		−0.6	−0.8	×	

Color coding: from orange (weak correlation) to green (strong correlation). × depicts an absent significant correlation of the same sign next to the strong correlation.

**Table 8:** Summary of found strong and significant Spearman rank correlations ($r_{S}$ ≥ 0.8) between PROs of social support (MSPSS scale) and TechROs (Fitbit wearable).

	PRO		TechRO			Correlation/Contour				
Health	Source	Variable	Amount	Family	Variable	Lower		$r_{S}$	Higher	
All	Significant other	Q2: shares joys and sorrows	Relative	CLR PA+S	Vigorous	+0.3	+0.7	+0.8		
All	Significant other	Q5: a real source of comfort	Relative	CLR PA+S	Vigorous	+0.4	+0.7	+0.8		
All	Significant other	Q10: cares about feelings	Relative	CLR PA+S	Vigorous	+0.5	+0.7	+0.8		
All	Family	Q3: tries to help	Relative	CLR PA+S	Fair		+0.3	+0.8	+0.7	
All	Family	Q8: talks about problems	Relative	CLR PA+S	Fair		+0.6	+0.8	+0.8	
All	Family	Q8: talks about problems	Relative	CLR PA+S	Vigorous	+0.6	+0.8	+0.8		
All	Family	Numeric sub-score	Relative	CLR PA+S	Fair		+0.3	+0.8	×	
Healthy	Significant other	Q1: around when in need	Absolute	Processed	Fair		×	−0.9	−0.6	
Healthy	Significant other	Q2: shares joys and sorrows	Absolute	Processed	Fair		×	−0.9	−0.7	−0.4
Healthy	Significant other	Q5: a real source of comfort	Absolute	Processed	Fair		×	−0.9	−0.6	
Healthy	Significant other	Q5: a real source of comfort	Relative	CLR PA+S	Fair		+0.4	+0.8	+0.6	
Healthy	Significant other	Q10: cares about feelings	Absolute	Processed	Fair		×	−0.8	−0.7	−0.7
Healthy	Significant other	Numeric sub-score	Absolute	Processed	Fair		×	−0.9	−0.6	−0.5
Healthy	Family	Q3: tries to help	Absolute	Processed	Fair		×	−0.8	−0.6	
Healthy	Family	Q3: tries to help	Relative	CLR PA+S	Fair	+0.5	+0.5	+0.9	+0.6	
Healthy	Family	Q8: talks about problems	Absolute	Processed	Fair		×	−0.8	−0.5	−0.4
Healthy	Family	Q8: talks about problems	Relative	CLR PA+S	Fair	+0.6	+0.5	+0.8	+0.6	
Healthy	Family	Q11: willing to help make decisions	Relative	CLR PA	Fair		+0.4	+0.8	×	
Healthy	Family	Numeric sub-score	Relative	CLR PA+S	Fair	+0.5	+0.4	+0.8	+0.4	
Healthy	Friends	Q9: share joys and sorrows	Absolute	Processed	Light		×	+0.8	+0.7	+ 0.4
Healthy	Friends	Q12: talk about problems	Absolute	Processed	Light		×	+0.8	+0.7	
Healthy	All	Categorical score	Absolute	Processed	Active			+0.8		
Healthy	All	Categorical score	Relative	CLR PA	Light		×	+0.8	×	
Healthy	All	Numeric score	Absolute	Processed	Light+Fair		+0.7	+0.8	×	
Healthy	All	Numeric score	Relative	CLR PA+S	Fair	+0.6	+0.5	+0.8	+0.4	
Diseased	Family	Q4: gives emotional help and support	Absolute	Processed	Sedentary			−0.8	×	
Diseased	Friends	Q12: talk about problems	Absolute	Raw	Steps			+0.8		

Color coding: from orange (weak correlation) to green (strong correlation). × depicts an absent significant correlation of the same sign next to the strong correlation.

**Table 9:** Summary of found strong and significant Spearman rank correlations ($r_{S}$ ≥ 0.8) between PROs of anxiety and depression (GADS scale) and TechROs (Fitbit wearable).

	PRO		TechRO			Correlation/Contour			
Health	Outcome	Variable	Amount	Family	Variable	Lower	$r_{S}$	Higher	
All	Anxiety	Q3A: irritable	Relative	CLR PA	Vigorous	×	+0.8		
All	Anxiety	Q5A: sleeping poorly	Relative	CLR PA+S	Light	+0.5	+0.8	+0.5	+0.3
All	Anxiety	Q7A: trembling	Absolute	Processed	Active		−0.8		
All	Depression	Q1D: lacking energy	Relative	CLR PA+S	Vigorous	×	−0.8		
All	Depression	Q6D: lost weight due to poor appetite	Relative	CLR PA+S	Light	×	+0.8	×	
All	Both	Numeric score	Relative	CLR PA+S	Sleep		+0.8		
Healthy	Anxiety	Q3A: irritable	Absolute	Processed	Active		−0.8		
Healthy	Anxiety	Q7A: trembling	Absolute	Processed	Light+fair	−0.5	−0.8	−0.5	
Healthy	Anxiety	Q7A: trembling	Absolute	Processed	Vigorous	×	+0.8		
Healthy	Anxiety	Q7A: trembling	Absolute	Processed	Active		−0.8		
Healthy	Anxiety	Q7A: trembling	Relative	CLR PA	Light	×	−0.8	×	
Healthy	Anxiety	Q7A: trembling	Relative	CLR PA+S	Vigorous	×	+0.8		
Healthy	Depression	Q2D: lost interest in things	Relative	CLR PA	Light	×	−0.8	×	
Healthy	Depression	Q6D: lost weight due to poor appetite	Relative	CLR PA+S	Sleep		+0.8		
Healthy	Depression	Q9D: worse in the morning	Relative	CLR PA+S	Sedentary		+0.8	×	

Color coding: from orange (weak correlation) to green (strong correlation). × depicts an absent significant correlation of the same sign next to the strong correlation.

**Table 10:** Summary of found strong and significant Spearman rank correlations ($r_{S}$ ≥ 0.8) between PROs of Mediterranean nutrition (PREDIMED scale) and TechROs (Fitbit wearable).

	PRO	TechRO			Correlation/Contour		
Health	Variable	Amount	Family	Variable	Lower	$r_{S}$	Higher
All	Q12: nuts use	Absolute	Processed	Fair	×	−0.9	×
All	Q12: nuts use	Relative	CLR PA+S	Light	+0.6	+0.8	×
All	Numeric score	Absolute	Processed	Vigorous	−0.7	−0.8	
All	Numeric score	Relative	CLR PA+S	Light	+0.6	+0.8	+0.6
Healthy	Q3: vegetables use	Relative	CLR PA	Fair	×	−0.8	×
Healthy	Q3: vegetables use	Relative	CLR PA+S	Fair	×	−0.8	−0.4
Diseased	Q5: red meat, hamburger, or meat use	Absolute	Raw	Energy		+0.8	
Diseased	Q11: commercial sweets or pastries use	Absolute	Raw	Heart rate		+0.8	

Color coding: from orange (weak correlation) to green (strong correlation). × depicts an absent significant correlation of the same sign next to the strong correlation.

**Table 11:** Summary of found strong and significant Spearman rank correlations ($r_{S}$ ≥ 0.8) between PROs of nutrition (SelfMNA scale) and TechROs (Fitbit wearable).

	PRO	TechRO			Correlation/Contour		
Health	Variable	Amount	Family	Variable	Lower	$r_{S}$	Higher
Diseased	Q1: food intake declined	Relative	CLR PA+S	Sleep		−0.8
Diseased	Q2: weight lost	Relative	CLR PA+S	Sleep		−0.8
Diseased	Q4: stressed or severely ill	Absolute	Processed	Sedentary		−0.8	×

Green (strong correlation). × depicts an absent significant correlation of the same sign next to the strong correlation.

**Table 12:** Summary of found strong and significant Spearman rank correlations ($r_{S}$ ≥ 0.8) between PROs of memory (MFE scale) and TechROs (Fitbit wearable).

	PRO	TechRO			Correlation/Contour				
Health	Variable	Amount	Family	Variable	Lower		$r_{S}$	Higher	
All	Q7: completely forgetting to take things	Relative	CLR PA+S	Sleep			+0.8		
All	Q12: having difficulty picking up a new skill	Relative	CLR PA+S	Light		×	−0.8	×	
All	Q13: finding a word on the tip of the tongue	Relative	CLR PA+S	Sleep			+0.8		
All	Q24: forgetting where things are normally kept	Relative	CLR PA	Fair		×	+0.8	×	
All	Q24: forgetting where things are normally kept	Relative	CLR PA+S	Fair		×	−0.8	−0.3	
All	Numeric score	Absolute	Processed	Active			−0.8		
Healthy	Q7: completely forgetting to take things	Relative	CLR PA+S	Sleep			+0.8		
Healthy	Q10: letting ramble about unimportant things	Absolute	Processed	Light+fair		×	−0.8	×	
Healthy	Q14: forgetting to do planned things	Absolute	Processed	Fair+vigorous		×	+0.8	+0.8	
Healthy	Q14: forgetting to do planned things	Absolute	Processed	Vigorous		+0.8	+0.8		
Healthy	Q16: forgetting the topic of an ongoing conversation	Absolute	Processed	Fair		×	−0.8	−0.4	
Healthy	Q24: forgetting where things are normally kept	Relative	CLR PA+S	Fair		×	−0.8	×	
Healthy	Numeric score	Relative	CLR PA	Fair		×	−0.8	×	
Diseased	Q1: forgetting objects put	Relative	CLR PA+S	Vigorous		−0.7	−0.8		
Diseased	Q6: forgetting the time of events	Absolute	Raw	Heart rate			+0.8		
Diseased	Q6: forgetting the time of events	Absolute	Processed	Light		+0.7	+0.8	×	
Diseased	Q6: forgetting the time of events	Absolute	Processed	Sleep			−0.8		
Diseased	Q8: being reminded about things	Absolute	Processed	Light+fair		+0.6	+0.8	×	
Diseased	Q9: reading anew something already read	Absolute	Processed	Sleep			−0.8		
Diseased	Q13: finding a word on the tip of the tongue	Absolute	Processed	Active			−0.8		
Diseased	Q13: finding a word on the tip of the tongue	Relative	CLR PA+S	Sedentary			+0.8	+0.7	
Diseased	Q18: forgetting to tell somebody something important	Absolute	Processed	Fair		×	−0.8	−0.8	−0.8
Diseased	Q18: forgetting to tell somebody something important	Absolute	Processed	Fair+vigorous		−0.8	−0.8	−0.8	
Diseased	Q18: forgetting to tell somebody something important	Absolute	Processed	Vigorous	−0.8	−0.8	−0.8		
Diseased	Numeric score	Absolute	Processed	Active			−0.8		

Color coding: from orange (weak correlation) to green (strong correlation). × depicts an absent significant correlation of the same sign next to the strong correlation.

**Table 13:** Summary of found strong and significant Spearman rank correlations ($r_{S}$ ≥ 0.8) between PROs of sleep (PSQI scale) and TechROs (Fitbit wearable).

	PRO	TechRO			Correlation/Contour				
Health	Variable	Amount	Family	Variable	Lower		$r_{S}$	Higher	
All	Q5A: trouble sleeping due to not getting to sleep	Relative	CLR PA+S	Sleep			+0.8		
All	Q5E: trouble sleeping due to coughing or snoring loudly	Relative	CLR PA	Vigorous		−0.5	−0.8		
All	Q5F: trouble sleeping due to feeling too cold	Relative	CLR PA+S	Light		+0.6	+0.8	+0.6	
All	Q7: trouble staying awake while driving, eating, socializing	Relative	CLR PA	Light		−0.5	−0.8	×	
All	Q7: trouble staying awake while driving, eating, socializing	Relative	CLR PA+S	Sleep			−0.8		
All	Latency numeric sub-score	Relative	CLR PA+S	Sleep			+0.8		
All	Efficiency numeric sub-score	Relative	CLR PA	Fair		×	+0.8	×	
All	Daily dysfunction numeric sub-score	Absolute	Processed	Vigorous	+0.5	+0.5	+0.8		
All	Daily dysfunction numeric sub-score	Relative	CLR PA	Light		−0.6	−0.8	×	
All	Daily dysfunction numeric sub-score	Relative	CLR PA+S	Sleep			−0.8		
Healthy	Q2: duration taken to fall asleep	Relative	CLR PA+S	Sleep			+0.8		
Healthy	Q3: time gotten up in the morning	Absolute	Raw	Energy			−0.8		
Healthy	Q5A: trouble sleeping due to not getting to sleep	Relative	CLR PA+S	Sleep			+0.8		
Healthy	Q5B: trouble sleeping due to waking up in the middle of the night	Relative	CLR PA+S	Vigorous		×	+0.8		
Healthy	Q5C: trouble sleeping due to using the bathroom	Absolute	Processed	Light+Fair		−0.5	−0.8	×	
Healthy	Q5C: trouble sleeping due to using the bathroom	Relative	CLR PA	Light		×	−0.8	−0.5	−0.6
Healthy	Q5E: trouble sleeping due to coughing or snoring loudly	Relative	CLR PA+S	Light		×	−0.8	×	
Healthy	Q11: duration stayed in bed	Relative	CLR PA+S	Sleep			+0.8		
Healthy	Numeric score	Absolute	Processed	Fair+vigorous		×	+0.8	+0.6	
Healthy	Latency numeric sub-score	Relative	CLR PA+S	Sleep			+0.8		
Healthy	Efficiency numeric sub-score	Relative	CLR PA	Fair		×	+0.8	×	
Diseased	Q1: time gone to bed at night	Absolute	Processed	Sleep			−0.8		
Diseased	Q4: duration of actual sleep	Absolute	Processed	Fair		×	+0.8	+0.8	+0.9
Diseased	Q4: duration of actual sleep	Absolute	Processed	Fair+vigorous		+0.8	+0.8	+0.9	
Diseased	Q4: duration of actual sleep	Absolute	Processed	Vigorous	+0.8	+0.8	+0.9		
Diseased	Q5B: trouble sleeping due to waking up in the middle of the night	Absolute	Raw	Energy			−0.8		
Diseased	Q5C: trouble sleeping due to using the bathroom	Absolute	Raw	Energy			−0.8		

Color coding: from orange (weak correlation) to green (strong correlation). × depicts an absent significant correlation of the same sign next to the strong correlation.

**Table 14:** Summary of found strong and significant Spearman rank correlations ($r_{S}$ ≥ 0.8) between PROs of health-related Quality of Life (EQ-5D-3L scale) and TechROs (Fitbit wearable).

	PRO		TechRO			Correlation/Contour		
Health	Domain	Variable	Amount	Family	Variable	Lower	$r_{S}$	Higher
Diseased	Anxiety/depression	Q5: anxiety/depression	Absolute	Processed	Sedentary		+0.8	×

Color coding: green (strong correlation). × depicts an absent significant correlation of the same sign next to the strong correlation.

**Table 15:** Gradient of correlations by interval durations (columns) and leeways (rows) in days.

	7	14	21	28	60	90	120
0	$-0.09^{11}$	$0.19^{9}$	$0.13^{9}$	$0.88^{9}$	$0.44^{8}$	$-0.25^{8}$	$0.14^{8}$
7	$-0.27^{16}$	$0.19^{9}$	$0.13^{9}$	$0.88^{9}$	$0.44^{8}$	$-0.36^{9}$	$0.14^{8}$
14	$-0.07^{23}$	$0.16^{16}$	$-0.03^{10}$	$0.88^{9}$	$0.44^{8}$	$-0.36^{9}$	$0.14^{8}$
21	$-0.07^{23}$	$-0.08^{20}$	$-0.14^{16}$	$0.92^{11}$	$0.44^{8}$	$-0.36^{9}$	$0.27^{9}$
28	$-0.07^{23}$	$-0.08^{20}$	$0.01^{17}$	$0.61^{13}$	$0.19^{9}$	$-0.36^{9}$	$0.27^{9}$
60	$-0.07^{23}$	$-0.09^{23}$	$-0.13^{21}$	$0.57^{20}$	$0.17^{10}$	$-0.36^{9}$	$0.27^{9}$
90	$-0.09^{24}$	$-0.06^{24}$	$-0.16^{22}$	$0.48^{21}$	$-0.08^{14}$	$-0.13^{10}$	$0.27^{9}$
120	$-0.06^{25}$	$-0.06^{24}$	$-0.16^{22}$	$0.48^{21}$	$-0.14^{15}$	$0.09^{16}$	$-0.10^{12}$

Color coding: from yellow (weaker correlations) to green (stronger correlations). Superscript depicts sample size. Subscript depicts sign. All correlations are shown. Only significant correlations are highlighted.

**Table 16:** Raw data for a 28-day interval and a 21-day leeway that yielded the highest correlation (0.92).

Participant ID	Wave	Q3 (PRO)	Fair (TechRO)
617	2	4	−1.49
419	1	5	−1.54
419	2	5	−1.48
643	2	6	−1.24
793	3	6	+1.05
170	3	6	+1.49
569	1	7	+2.10
133	2	7	+1.73
569	2	7	+2.09
133	3	7	+1.69
569	3	7	+1.88

Color coding: from orange (lower values) to yellow to green (higher values).

**Table 17:** Summary of Characteristics of PRO (IPAQ, MSPSS, GADS, PREDIMED, SelfMNA, MFE, PSQI, EQ-5D-3L) and median TechRO (Fitbit) over the measurement period corresponding to each wave for Participant 169.

ID	Health	Wave	Country	Gender	Age	Physical Activity (IPAQ): Numeric Score	Social Support (MSPSS): Numeric Score	Depression and Anxiety (GADS): Numeric Score	Nutrition Mediterranean (PREDIMED): Numeric Score	Nutrition (SelfMNA): Numeric Score	Memory (MFE): Numeric Score	Sleep (PSQI): Numeric Score	Quality of Life (EQ-5D-3L): Health Score	TechRO: Energy (Kcal.)	TechRO: Steps (count)	TechRO: Heart Rate (bpm.)	TechRO: Sedentary (min.)	TechRO: Sedentary+Light (min.)	TechRO: Light (min.)	TechRO: Light+Fair (min.)	Fair (min.)	TechRO: Fair+Vigorous (min.)	TechRO: Vigorous (min.)	TechRO: Active (min.)	TechRO: Sleep (h:min.)
169	Diseased	1	Hungary	Female	69		5	68	7	12	13	15	80	2044.0	8035.0	52.5	842.0	999.0	192.5	253.0	23.0	51.0	19.0	300.0	7:06
169	Diseased	2	Hungary	Female	69	7338	5	51	5		15	15	75	1889.0	6076.0	56.0	843.0	994.0	207.0	245.0	21.0	51.5	22.5	273.5	7:08
169	Diseased	3	Hungary	Female	69	21,702	5	47	7	12	8	14	80	1979.0	8172.0	55.0	798.0	975.0	204.0	248.0	40.0	70.0	33.0	294.0	7:03
Median						14,520.0	5.0	51.0	7.0	12.0	13.0	15.0	80.0	1979.0	8035.0	55.0	842.0	994.0	204.0	248.0	23.0	51.5	22.5	294.0	7:06
Mean						14,520.0	5.0	55.3	6.3	12.0	12.0	14.7	78.3	1970.7	7427.7	54.5	827.7	989.3	201.2	248.7	28.0	57.5	24.8	289.2	7:05
SD						10,156.9	0	11.2	1.2	0.0	3.6	0.6	2.9	77.8	1172.6	1.8	25.7	12.7	7.7	4.0	10.4	10.8	7.3	13.9	0:03

Color coding: from orange (worse outcome) to yellow to green (better outcome).

**Figure A1:**
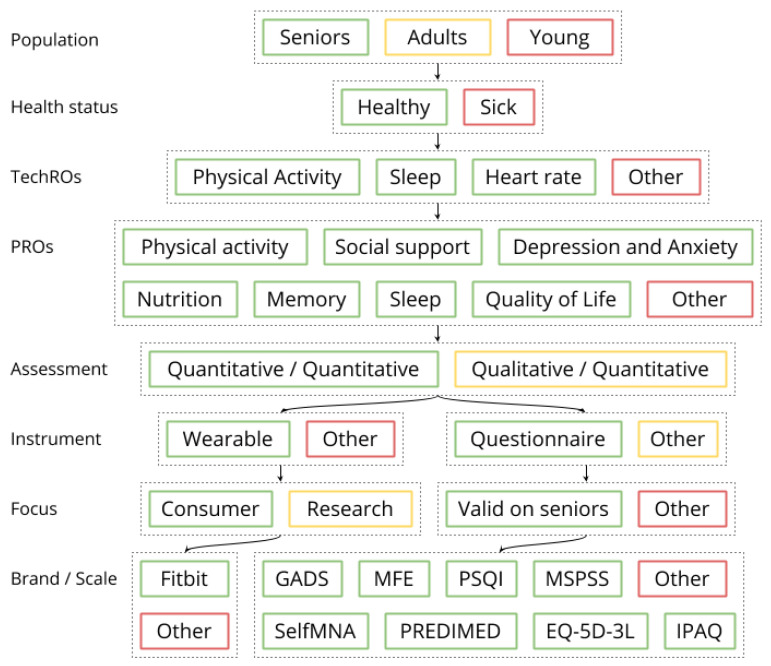
Related Work selection procedure (example on social support). Colors: green (positive towards inclusion), yellow (neutral), red (negative towards exclusion).

**Figure A2:**
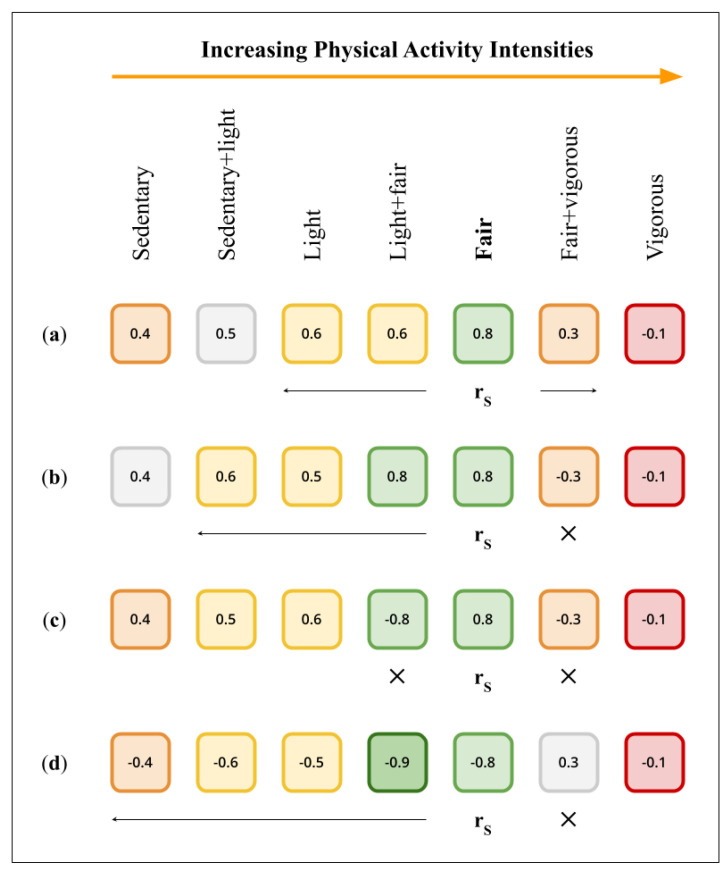
Examples of contours of correlations interrupted by non-significant or opposite-sign correlations. $r_{S}$ marks the target correlation. × marks an interruption. Arrows mark the width of the contour. Only significant correlations are colored from red (weak) to green (strong). In example (**a**), the contour is interrupted by a non-significant correlation (at a lower intensity) and an opposite-sign correlation (at a higher intensity). Example (**b**) interrupts the entire right side of the contour by an opposite-sign correlation, represented with ×. Example (**c**) depicts a singleton contour, marked with × on both sides. Example (**d**) illustrates the rare case of a higher correlation than the target correlation, both in the same contour.

**Figure A3:**
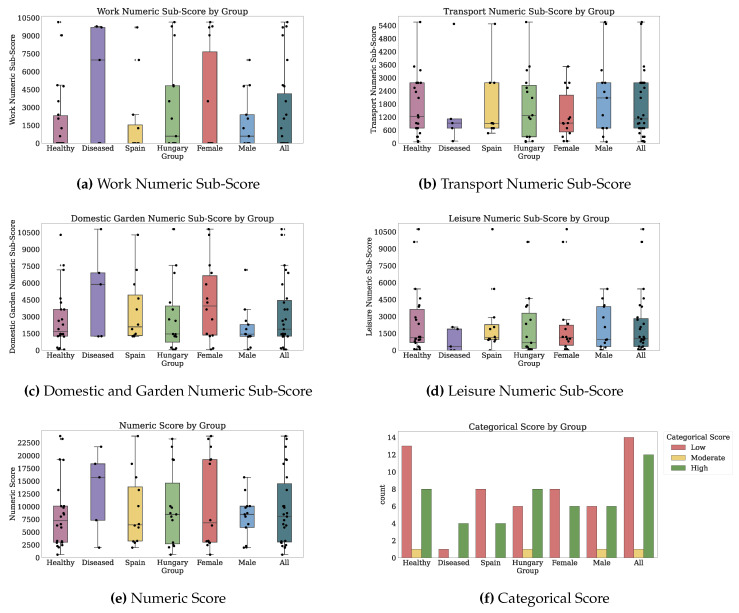
Sub-scores and Scores for Physical Activity (IPAQ).

**Figure A4:**
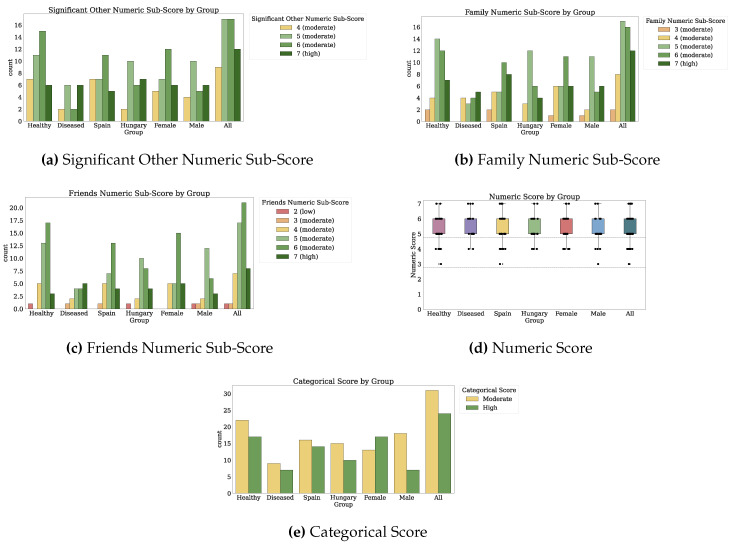
Sub-scores and Scores for Social Support (MSPSS). Dotted markings delimit levels of the categorical score.

**Figure A5:**
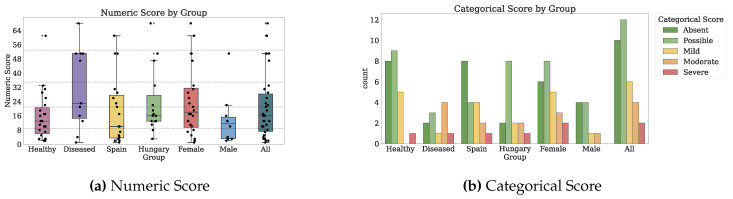
Scores for Anxiety and Depression (GADS). Dotted markings delimit levels of the categorical score.

**Figure A6:**
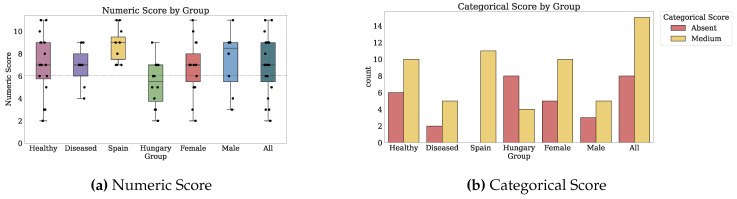
Scores for Mediterranean Nutrition (PREDIMED). Dotted markings delimit levels of the categorical score.

**Figure A7:**
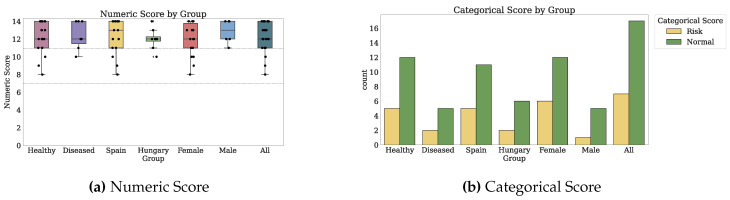
Scores for Nutrition (SelfMNA). Dotted markings delimit levels of the categorical score.

**Figure A8:**
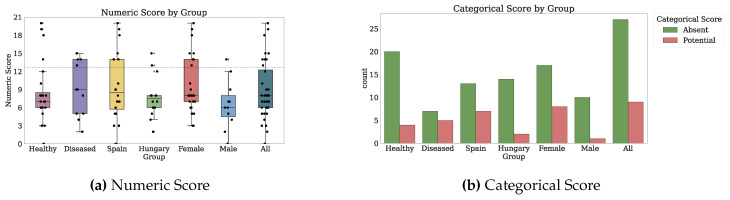
Scores for Memory (MFE). Dotted markings delimit levels of the categorical score.

**Figure A9:**
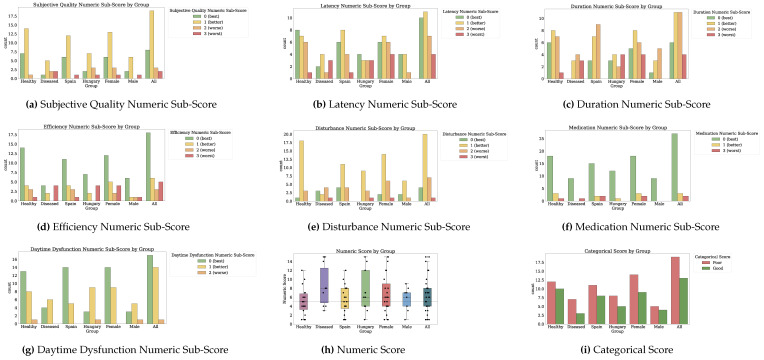
Sub-scores and Scores for Sleep (PSQI). Dotted markings delimit levels of the categorical score.

**Figure A10:**
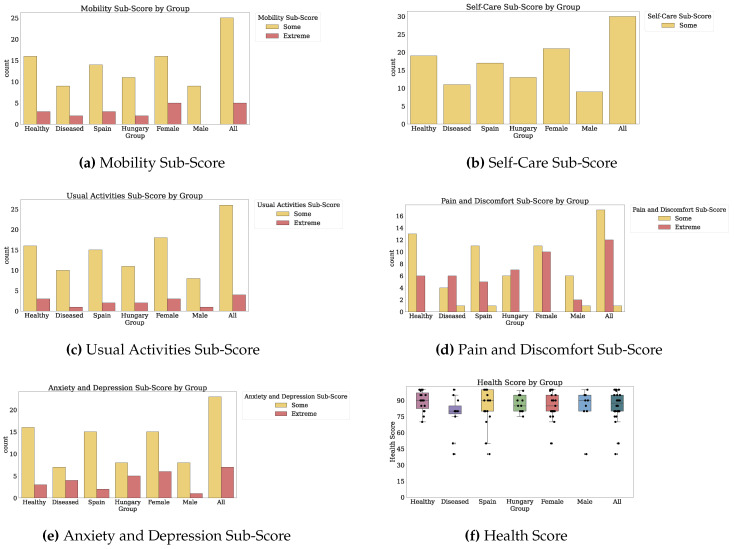
Sub-scores and Scores for Health-Related Quality of Life (EQ-5D-3L).

**Figure A11:**
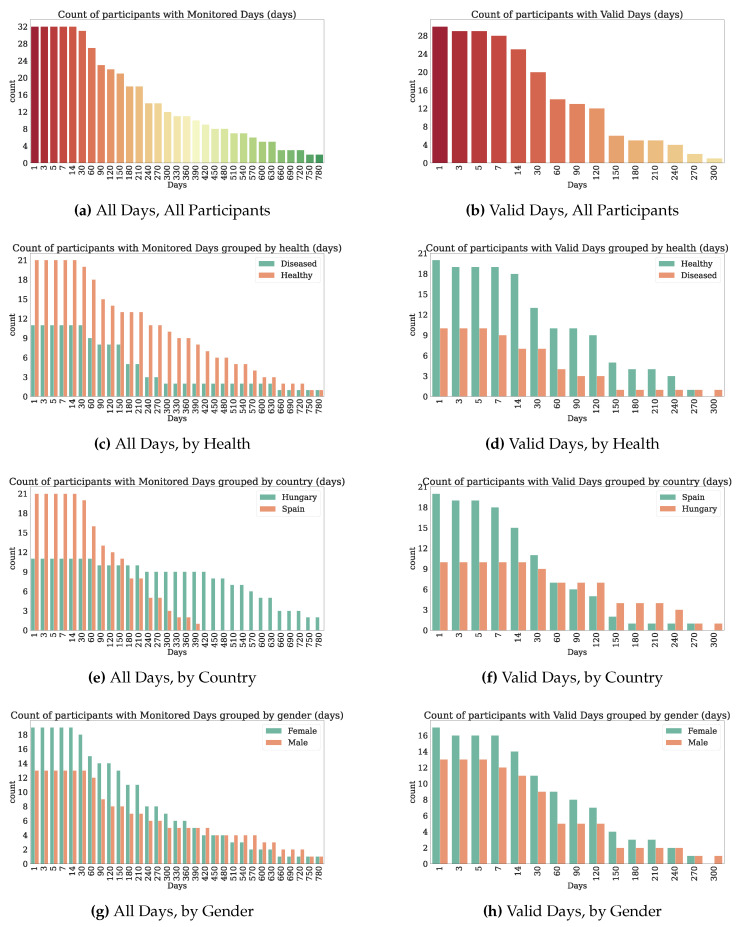
Count of seniors with at least the given valid days of Fitbit (TechRO) by group.

**Figure A12:**
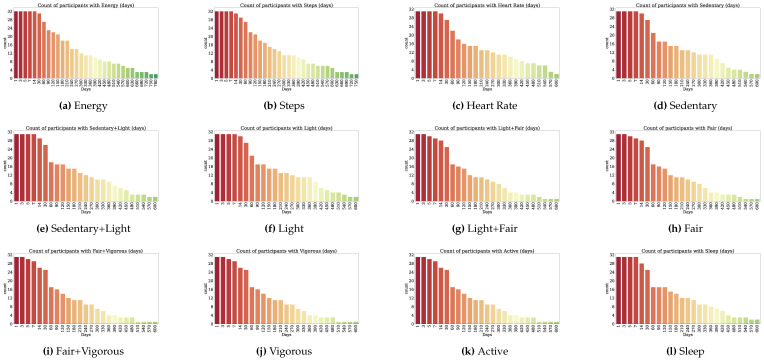
Count of seniors with at least the given valid days of Fitbit (TechRO) by outcome.

**Figure A13:**
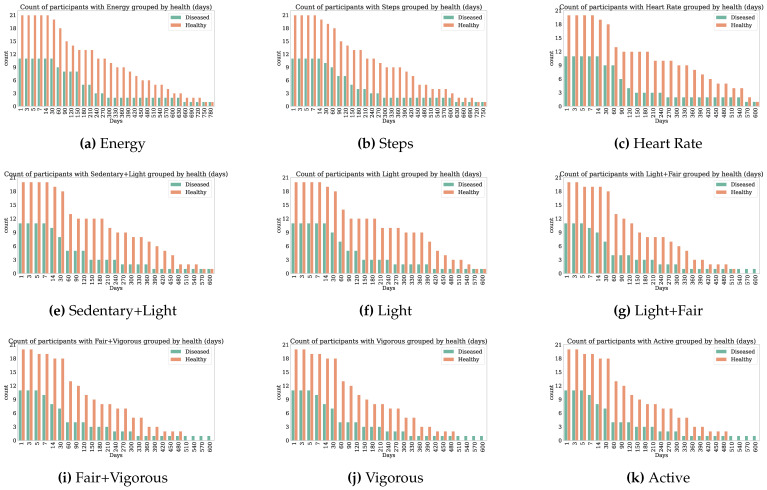
Count of seniors with at least the given valid days of Fitbit (TechRO) by outcome and health group.

**Figure A14:**
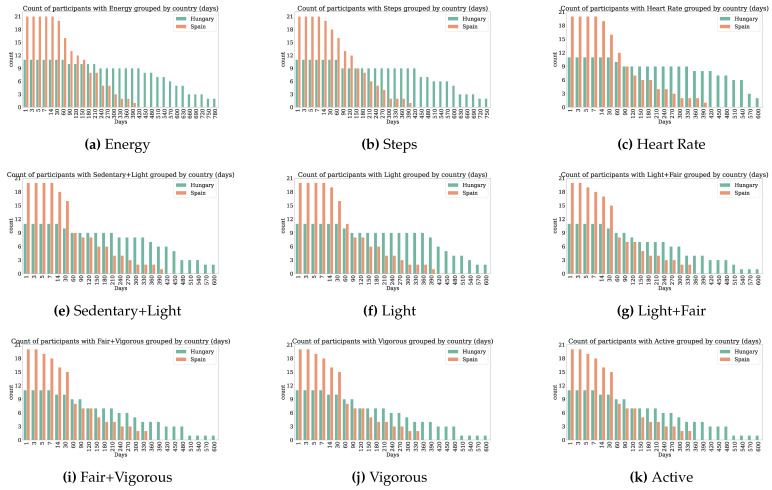
Count of seniors with at least the given valid days of Fitbit (TechRO) by outcome and country group.

**Figure A15:**
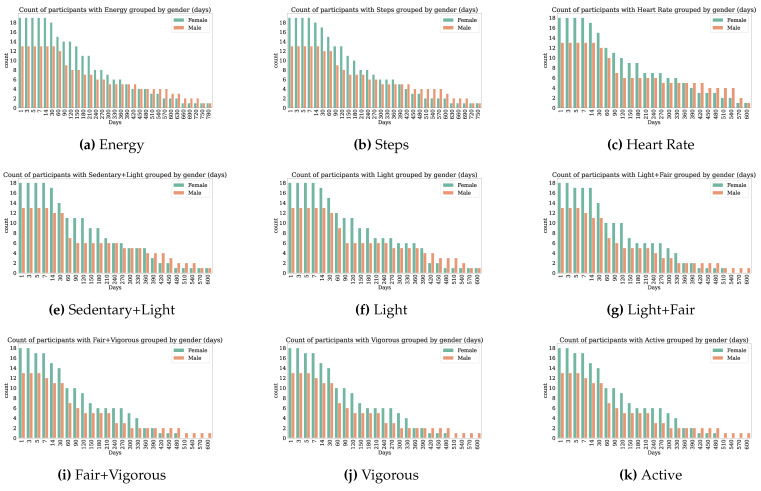
Count of seniors with at least the given valid days of Fitbit (TechRO) by outcome and gender group.

**Figure A16:**
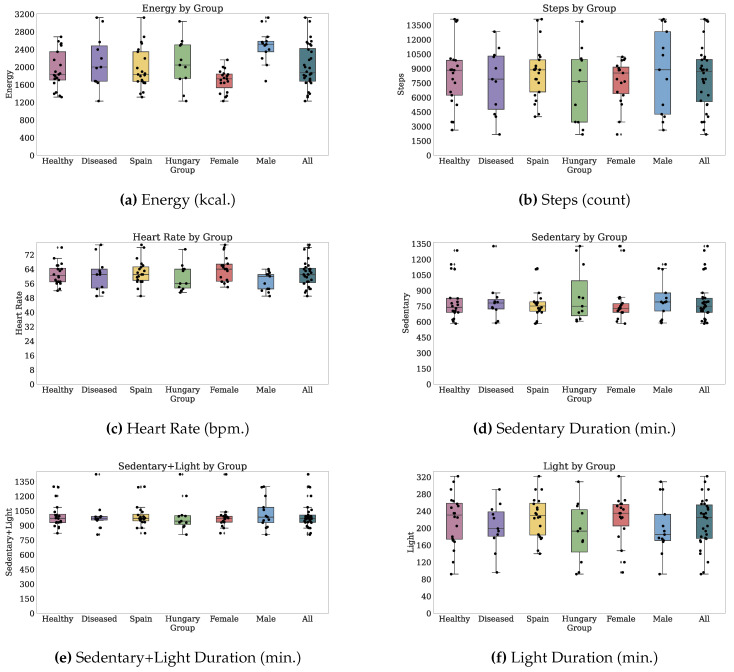
Median values of TechROs (Fitbit) across the entire monitoring period: energy, steps, heart rate, sedentary duration, sedentary+light duration, light duration (1 of 2).

**Figure A17:**
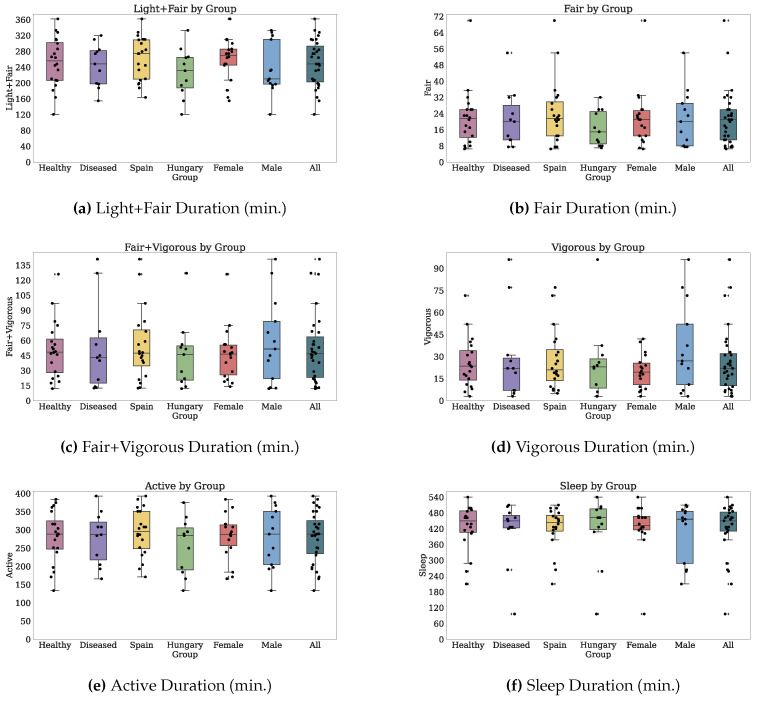
Median values of TechROs (Fitbit) across the entire monitoring period: light+fair duration, fair duration, fair+vigorous duration, vigorous duration, active duration, sleep duration (2 of 2).

## Abbreviations

The following abbreviations are used in this manuscript:

API	Application Programmable Interface
CLR	Centered Log Ratio
CLR PA	Centered Log Ratios of Physical Activity
CLR PA+S	Centered Log Ratios of Physical Activity and Sleep
CoME	Caregiver and Me
EQ-5D-3L	EuroQoL with 5 Domains and 3 Levels
GADS	Goldberg Anxiety and Depression Scale
IPAQ	International Physical Activity Questionnaire
MFE	Memory Failures of Everyday
MSPSS	Multidimensional Scale of Perceived Social Support
PREDIMED	Prevention with Mediterranean Diet
PRO	Patient-Reported Outcome
PSQI	Pittsburgh Sleep Quality Index
QoL	Quality of Life
SD	Standard Deviation
SelfMNA	Mini Nutritional Assessment
TechRO	Technology-Reported Outcome

## Appendix A. Literature Review

This section describes our procedure for literature review (Appendix A.1).

### Appendix A.1. Literature Review Procedure

We searched for previous work by following a semi-structured approach, to prune papers distant from our research area from a vast body of literature. We agreed upon a hierarchy with properties divided into positive, neutral, and negative by their relative relevance to our research area (Figure A1).

We began by including papers related to the PRO and using TechROs to the first level. We then followed a depth-first procedure of paper inclusion and exclusion. At each level, we included papers from the parent level and excluded all papers without positive properties for that level.

We then prioritized the papers by their deepest level of inclusion. We set the exclusion threshold at studies where the two outcomes, one PRO, and one TechRO, are used for co-calibration. We allowed only the PROs assessed in this paper (with a preference for the same questionnaires) and for TechROs provided by consumer wearables or accelerometers (with a preference for consumer wearables).

Numerous research directions and studies were excluded from our literature review reporting. We exclude papers that do not use PROs (or compare PROs) [72], do not use TechROs (or compare TechROs) [73], use other TechROs than wearables (e.g., smart phones [74], smart home [75], internet of things [76], medical imaging such as computer tomography or magnetic resonance [77]), focus on recognizing activities of daily life [78], or report only results following interventions [79].

## Appendix B. Materials and Methods

In this section, we append notes on our materials and methods regarding patient-reported outcomes (Appendix B.1), technology-reported outcomes (Appendix B.2), and the co-calibration using coQoL (Appendix B.3).

### Appendix B.1. Patient-Reported Outcomes (Questionnaires)

This part elaborates on our materials and methods for assessing the patient-reported outcomes: the used questionnaires (Appendix B.1.1), the administration of the questionnaires (Appendix B.1.2), the scoring of the answers (Appendix B.1.3), and the derivation of PRO variables (Appendix B.1.4).

#### Appendix B.1.1. Questionnaires

The participants provided PRO answers on questionnaires for physical activity (IPAQ [26]), social support (MSPSS [27]), anxiety and depression (GADS [28]), Mediterranean nutrition (PREDIMED [29,30]), nutrition (SelfMNA [31]), memory (MFE [32]), sleep (PSQI [33]), and health-related quality of life (EQ-5D-3L [34]). Table A1 illustrates the PRO questionnaires.

**Table A1:** Questionnaires with validated scales for PROs.

Outcome	Scale	Administration	Scoring
Profile	-	27 items assessing: age, gender, ethnicity, profession, education, cohabitants, height, weight, blood pressure, cholesterol, smoking, alcohol, medication (hypertension), personal health history (diabetes, apnea, insomnia, hyperglycemia, stroke, infarct, depression), and family health history (hypertension, diabetes, stroke, infarct, dementia)	-
Physical Activity	International Physical Activity Questionnaire (IPAQ) [26]	27 items of mixed types: yes/no, counts of days of physical activity per week, durations of physical activity per day. Recall: 2 weeks	Numeric score (estimated effort in metabolic equivalent of task). Categorical score with 3 levels: 0 low, 1 moderate, and 2 high. Numeric sub-scores for domains (work, leisure, transport, domestic and garden) and intensities of physical activity (sedentary, low, moderate, and vigorous).
Social Support	Multi-Dimensional Scale Perceived Social Support (MSPSS) [27]	12 items on a 7-level Likert scale (Q1–Q12). Recall: indefinite	Numeric score increasing with social support (1–2.9: low, 3–5: moderate, 5.1–7: high). Categorical score with 3 levels: 0 low, 1 moderate, and 2 high. Numeric sub-scores (1–7) for three sources of social support: significant other, family, and friends.
Anxiety and Depression	Goldberg depression and anxiety scale (GADS) [28]	18 items: 9 for Anxiety (denoted Q1A–Q9A), 9 for Depression (Q1D-Q9D), all on a 6-level Likert scale. The original answers were on a 2-level Likert scale. The collected answers are on a 6-level Likert scale. Recall: 1 month	Numeric score increasing with depression and anxiety: 0–9 no depression, 10–21 possible depression, 22–35 mild depression, 36–53 moderate depression, and 54–90 severe depression. Categorical score with 5 levels: 0 absent, 1 possible, 2 mild, 3 moderate, 4 severe.
Nutrition Mediterranean	Prevention with Mediterranean Diet (PREDIMED) [29,30]	14 binary items: 2 items yes/no, 12 items with thresholds for ingested food quantity (Q1–Q14). Recall: indefinite	Numeric score from 0–6 for no adherence to 7–12 for medium adherence to 13–14 for high adherence. Categorical score with 3 levels: 0 absent, 1 medium, 2 high.
Nutrition	Self-Reported Mini Nutritional Assessment (SelfMNA) [31]	6 items: 5 on various levels Likert scales, 1 binary (Q1–Q6). Recall: 3 months, same day	Numeric score from 0–7 for malnourished to 8–11 for risk of malnutrition to 12–14 for normal nutrition. Categorical score with 3 levels: 0 for malnutrition, 1 for risk, and 2 for normal nutrition.
Memory	Memory Failures of Everyday (MFE) [32]	28 items on a 3-level Likert scale (Q1–Q28). Recall: indefinite	Numeric score from 0 for no memory failures to 56 for potential memory failures. Categorical score separating 0 for no memory failures and 1 for potential memory failures, by comparing with deviations from the mean.
Sleep	Pittsburgh Sleep Quality Index (PSQI) [33]	25 items of mixed types: durations, yes/no, Likert scales (Q1, ..., Q4, Q5A, ..., Q5J, Q6, ..., Q9). Recall: 1 month	Numeric score increasing as sleep quality decreases on a 0-21 scale. Categorical score of 1 for good sleep quality (0–4) and 0 for poor sleep quality (5–21). Numeric sub-scores (0–7) for: quality, latency, duration, efficiency, disturbance, medication, and daytime dysfunction.
Health-Related Quality of Life	EuroQoL health questionnaire (EQ-5D-3L) [34]	6 items: 5 on a 3-level Likert scale (denoted by their measured outcomes), 1 on a visual analog scale (Q1–Q6). Recall: same day	Numeric scores for five domains: mobility, self-care, usual activities, pain/discomfort, and anxiety/depression, for the Likert items, increasing from 1 to 3 as life quality decreases. Visual analog scale of health state on the day of administration (giving a health score of 0–100), where higher numbers indicate better health.

#### Appendix B.1.2. Questionnaire Administration

For the participants in Spain, the partners used already available versions of the questionnaires in Spanish [80,81,82,83,84,85,86,87]. For the participants in Hungary, only some questionnaires had variants in Hungarian [88]. The local partners in the project translated the missing questionnaires from English to Hungarian (and assured the translation accuracy) to allow all participants to fill the PROs in their respective languages.

#### Appendix B.1.3. Answers Scoring

For the PRO questionnaires, we followed the scoring procedures set forth by the authors of the validated scales associated with each questionnaire. Only one questionnaire necessitated an additional assumption. For the physical activity questionnaire (IPAQ), we processed the individuals’ physical activity answers by adhering to the data cleaning, maximum values for excluding outliers as described in the guide [89]. However, the guide does not provide a threshold for converting the *duration reported as weekly (not daily) to daily into an average daily time*. For example, if a senior reported seven hours of vigorous physical activity per day, the duration would likely reflect one hour per day. In this case, we allowed at most 7 h of physical activity per day at any intensity by dividing all excessive durations by 7 days.

#### Appendix B.1.4. Variables Derivation

We derived variables from both individual items, sub-scores, and scores of PRO scales. While the analysis of the scores exclusively would have been motivated by existing Rasch models providing calibrated positions of individual items and their sub-scores and scores [90], to our knowledge, there are no Rasch models for the PRO scales. Table 2 presents the derived PRO variables.

### Appendix B.2. Technology-Reported Outcomes (Fitbit)

This part elaborates on our materials and methods for assessing the technology-reported outcomes: motivation and considerations for the Fitbit Charge 2 wearable (Appendix B.2.1), the processing of the wearable data (Appendix B.2.2), and the derivation of TechRO variables (Appendix B.2.3).

#### Appendix B.2.1. Fitbit Consumer Wearable

The space of consumer wearable manufacturers and devices is diverse, recording over 200 models [91], and the trend of adoption is increasing [13]. From all devices that provide physical activity and sleep TechROs, we chose Fitbit. Fitbit (1) monitors daily life behaviours accurately and continuously, (2) operationalizes the critical human factors for prolonged wear by senior end-users, and (3) facilitates reliable behavioural data collection.

First, Fitbit aims at motivating consumers to *“reach health and fitness goals by tracking activity, exercise, sleep, weight, and more”* [35]. It was selected for Digital Health software pre-certification by the US FDA [92]. Previous studies measured the accuracy of Fitbit consumer-friendly devices in reporting daily life behaviours of physical activity and sleep. For physical activity, Fitbit One and Zip had strong validity for step count and sleep duration, moderate for energy expenditure, and were weaker for fair and vigorous activity [12]. Fitbit Flex and Zip had adequate reliability and validity in measuring step count [93]. Fitbit Charge HR, Charge, Flex, Surge, Zip, and Alta agree with the ActiWatch GT3X+ research-grade accelerometer in assessing active minutes [37]. For sleep, Fitbit Charge HR can measure total sleep time [94] and time spent in bed [95] reliably, as compared with a sleep diary in a free-living setting or a research-grade accelerometer. For senior populations, Fitbit Charge 2 had better results in step count, energy expenditure, and sleep duration than the Garmin Vivosmart HR+ accelerometer in free-living environments [96]. Also, Fitbit One and Flex measure steps accurately in seniors [97].

Second, the positive senior user experience with the wearable is an essential factor that prolongs monitoring durations. For Fitbit, human factors studies found that over 90% of seniors agree that Fitbit was *“easy to use, useful, and acceptable”* over 8 months of wear [15] and seniors also place Fitbit the highest in usability (using the System Usability Scale [98]) among numerous other wearables [99]. Furthermore, the presence of a data display on the wristband leads to higher operation ratings [99].

Third, Fitbit provides a well-documented and developer-friendly application programming interface (API) which exposes a rich set of behavioural markers along [22] addressing goals of the project.

For our study, we selected the Fitbit Charge 2 wearable, a small wrist-worn watch which can monitor physical activity and sleep by using the same sensors such as those used in the validations, and displays steps, heart rate, and time, previously used in studies involving seniors (e.g., [96]).

#### Appendix B.2.2. Wearable Data Processing

To maintain high data quality, we considered *valid* days for the analysis only those days where the total duration of Fitbit monitoring was at least 21 h. We allowed at most three hours of missing data for device battery charging and handling (15–20 min to 2 h). Our choice reduced the impact of missing measurements and improved not only the measurement accuracy of TechRO behavioural markers in absolute daily durations but also enabled the assessment of TechRO behavioural markers relative to each other in the 24-h model of a day [64].

We constructed aggregate intervals with fixed durations of 7, 14, 21, 28, 60, 90, and 120 valid days to balance the number of included days in the analysis with the available intraday monitoring quality. The choice of 7 days for the lower bound was motivated by the need to acquire enough representative data for daily life, the 7 days as a common denominator of the PRO recall periods (where present), and the significant improvements in Fitbit accuracy for active minutes from 7 days onwards [37]. The choice of increasing intervals to the upper bound of 120 days reflected the duration of a wave, a large number of valid days per person (e.g., median 153 days for Spanish participants, Table A11), but also the high variance (a standard deviation of 113 days in Spain, Table A11).

We only included in the analysis intervals with at least 70% of their days valid, such that both weekdays and weekends were expected present in a week; the limit is compatible with previously reported consumer wearable use in seniors [100].

#### Appendix B.2.3. Variables Derivation

We split the TechROs into two amounts, *absolute* (behaviours in isolation, expressed in absolute amounts) and *relative* (behaviours relative to each other reflects the interdependences between behaviours during the 24 h of the day [64], expressed in relative amounts by the centred log ratios (CLR) of their compositions [65]).

In the absolute amount, we derived the variables into two families: *raw* and *processed*. We derived the raw daily energy expenditure (*energy*), step count (*steps*), and resting heart rate (*heart rate*) towards a total of 3 raw TechROs. We then derived the processed sedentary duration (*sedentary*), and the duration at three intensities (*light*, *moderate*, and *vigorous*) as processed by the Fitbit internal activity recognition algorithms. Since Fitbit had not published intensity thresholds, we also derived the cumulative durations in processed sedentary and light (*sedentary+light*), light and fair (*light+fair*), and fair and vigorous (*fair+vigorous*) intensities. We also calculated the total daily active duration (*active*) cumulating the light, fair, and vigorous processed durations. For sleep, we included the entire sleep duration of the day as a processed TechRO towards a total of 9 processed TechROs. We derived a total of 12 TechROs in the absolute amount.

For each aggregate interval duration and absolute TechRO, we used in the analysis as the aggregate the *median* from the absolute daily amounts as a variable. The 84 resulting variables are visible in the upper half of Table 3.

In the relative amount, we derived variables denoting compositional components of physical activity intensities and sleep throughout the day. We derived TechROs for each component of the centred log-ratio (*CLR*, [65]) transformation. The CLR is a symmetric transformation that does not require a reference component behaviour. We computed the CLRs of two families denoting distinct compositions: (1) from all physical activity durations (*CLR PA*) and (2) from all physical activity durations and the sleep duration (*CLR PA+S*), having 4 and 5 TechROs, respectively. We derived two relative families, as the CLRs of a composition do not translate to sub-compositions [65], but some studies may not be able to monitor sleep. We obtained a total of 9 TechROs in the relative amount.

For each aggregateinterval duration and relative TechRO, we used in the analysis as the aggregate the *geometric mean* from the relative daily amounts. The 63 resulting variables are visible in the lower half of Table 3.

The 147 derived TechRO variables can be seen in Table 3 (TechRO).

### Appendix B.3. Co-Calibration Using coQoL

This part elaborates on our method coQoL to co-calibrate PROs and TechROs. The part covers the three types of analysis: descriptive (Appendix B.3.1), inferential (Appendix B.3.2), and pattern (Appendix B.3.3).

#### Appendix B.3.1. Descriptive Analysis (PROs and TechROs)

We describe the PROs and TechROs from two perspectives. The first perspective refers to the *values in the data*. The second perspective refers to the *amount of data*.

Within the first perspective, we describe the PROs by observing three summary statistics (median, mean, and standard deviation) of the participants-waves when grouped by health status (healthy vs. (mildly) diseased), country (Spain vs. Hungary), and gender (male vs. female) (Table A3, Table A4, Table A5, Table A6, Table A7, Table A8, Table A9, Table A10).

Within the same perspective, we describe the TechROs by observing medians across the entire monitoring period (Table A12) in the first perspective.

Within the second perspective, we observe the counts of total and valid days (Table A11) within the same groups as for the first perspective.

#### Appendix B.3.2. Inferential Analysis (PROs vs. TechROs)

We set the leeway between PRO administration date and TechRO aggregate interval end date at (successively) 0, 7, 14, 21, 28, 60, 90, 120 days due to scarce exact matches. Pairs of variables with nearer such dates took precedence. We then analyzed lists of these pairs by using Spearman rank correlations. We chose this test as the best statistic to represent co-calibration motivated by the following assumptions. First, the PRO and TechRO variables were not independent (as they referred to the same participant). Second, the Spearman test is a nonparametric test that does not require an underlying distribution for the variables (some variables did not distribute normally, Shapiro Wilk normality test yielded p<0.05-and some variables measured different metrics). Third, our aim was holistic in observing groups of significant correlations (and not individual correlations).

We only report the strongest correlation per TechRO interval duration. We consider correlations between distinct constructs (e.g., PRO social support and TechRO sleep duration) to be strong at $r_{S}$ ≥ 0.5 and associations between similar constructs (e.g., PRO and TechRO physical activity) to be strong at $r_{S}$ ≥ 0.8.

We consider a correlation coefficient significant when the extremities of its 95% confidence interval have the same sign. We avoided effect omissions at the expense of potential effects due to chance by not using adjustments for multiple tests [101] as our focus is on observing groups of correlations rather than individual correlations.

#### Appendix B.3.3. Pattern Analysis (PROs vs. TechROs)

For the pattern analysis, the *contour* metric separately counts for a significant and strong target correlation for a physical activity intensity ($r_{S}$ 0.8 or above) the other significant correlations of the same sign at the lower and higher intensities. In case the intensity of the target correlation is at the extremity, the metric is undefined. In case the target correlation is adjacent to a correlation that has the opposite sign or is non-significant, the count on that side is 0. In case the correlation is unrelated to a physical activity intensity, this metric is undefined.

For example, the fair physical activity correlation 0.8 and the sequence of correlations [sedentary: 0.4*, sedentary+light: 0.5, light: 0.6*, light+fair: 0.6*, fair: 0.8*, fair+vigorous: 0.3*, and vigorous: −0.1*], where * denote significant correlations, has two correlations of lower intensities (0.6*, 0.6*) and one of higher intensity (0.3*). Figure A2 illustrates this case as Example (a). The figure contains three more examples.

## Appendix C. Results

This section includes results from our descriptive (Appendix C.1) and inferential analysis (Appendix C.2) analyses.

### Appendix C.1. Descriptive Analysis (PROs and TechROs)

This part includes results from our descriptive analysis from patient-reported outcomes (Appendix C.1.1) and technology-reported outcomes (Appendix C.1.2).

#### Appendix C.1.1. Patient-Reported Outcomes (Questionnaires)

The 39 participants provided 289 answers (7.4 ± 4.4) on the 8 scales along the 3 waves. Table A2 depicts the numeric scores across waves.

**Table A2:** PRO numeric scores from answers by questionnaire (N=39 participants).

						Physical Activity (IPAQ)				Social Support (MSPSS)				Anxiety and Depression (GADS)				Mediterranean Nutrition (PREDIMED)				Nutrition (SelfMNA)				Memory (MFE)				Sleep (PSQI)				Quality of Life (IPAQ)		
PID	Health	Country	Gender	Age		Wave 1	Wave 2	Wave 3		Wave 1	Wave 2	Wave 3		Wave 1	Wave 2	Wave 3		Wave 1	Wave 2	Wave 3		Wave 1	Wave 2	Wave 3		Wave 1	Wave 2	Wave 3		Wave 1	Wave 2	Wave 3		Wave 1	Wave 2	Wave 3
575	Healthy	Hungary	Female	65							5.0				8.0				5.0								7.0				3.0				85.0	
569	Healthy	Hungary	Female	67			23,238.0			7.0	6.0	6.0		19.0		11.0				7.0				11.0		8.0		6.0				4.0		80.0	80.0	95.0
133	Healthy	Hungary	Female	71			19,164.0	19,262.9		6.0	6.0	6.0		17.0		13.0				6.0		14.0				8.0		7.0		6.0		4.0		95.0	95.0	99.0
420	Healthy	Hungary	Female	71		576.0	2958.0			5.0	4.0				16.0				2.0				13.0				8.0				7.0			80.0	80.0	
215	Healthy	Hungary	Female	87		2446.0				5.0				33.0				3.0				10.0				8.0				12.0				80.0		
576	Healthy	Hungary	Male	60			2268.0				5.0																								95.0	
535	Healthy	Hungary	Male	69				8712.0				6.0				3.0				9.0				12.0				6.0				1.0				95.0
170	Healthy	Hungary	Male	70			8038.5	10,088.0			5.0	5.0				22.0				3.0								6.0				6.0			90.0	85.0
212	Healthy	Hungary	Male	72		8478.0	9793.5			5.0	4.0	5.0																						90.0	90.0	90.0
419	Healthy	Hungary	Male	95			2016.0			5.0	4.0				13.0				6.0				12.0				12.0				7.0			90.0	95.0	
643	Healthy	Spain	Female	67			23,793.0			7.0	6.0			2.0	7.0				9.0			14.0				8.0	3.0			1.0	4.0			100.0	90.0	
798	Healthy	Spain	Female	67								6.0																								90.0
803	Healthy	Spain	Female	67																												5.0				80.0
617	Healthy	Spain	Female	69			3186.0			4.0	4.0				61.0				7.0				13.0				19.0				10.0			100.0	90.0	
620	Healthy	Spain	Female	69			3264.4				6.0				29.0				10.0				9.0				20.0				2.0				90.0	
640	Healthy	Spain	Female	69						5.0				26.0								14.0				10.0				6.0				70.0		
628	Healthy	Spain	Female	70						7.0				3.0								11.0				5.0				1.0				100.0		
638	Healthy	Spain	Female	71			6303.0			6.0	6.0			10.0								12.0				7.0				5.0				100.0	100.0	
648	Healthy	Spain	Female	72						6.0				5.0				11.0				13.0				3.0				1.0				80.0		
649	Healthy	Spain	Female	72																						14.0								80.0		
795	Healthy	Spain	Female	72				2910.0				6.0				17.0				7.0				8.0				6.0				6.0				90.0
630	Healthy	Spain	Female	74						5.0				31.0								12.0				18.0				12.0				75.0		
411	Healthy	Spain	Male	45						5.0																								80.0		
790	Healthy	Spain	Male	66				10,101.0				6.0				3.0				11.0				14.0				7.0				4.0				100.0
700	Healthy	Spain	Male	67							3.0				10.0				8.0								7.0				9.0				40.0	
636	Healthy	Spain	Male	68			13,258.0			5.0	5.0																							40.0	40.0	
793	Healthy	Spain	Male	68				6560.0				5.0																								100.0
796	Healthy	Spain	Male	74				5907.0				4.0				2.0				9.0				14.0				0.0								80.0
502	Diseased	Hungary	Female	63																																80.0
169	Diseased	Hungary	Female	69			7338.0	21,702.0		5.0	5.0	5.0		68.0	51.0	47.0		7.0	5.0	7.0		12.0		12.0		13.0	15.0	8.0		15.0	15.0	14.0		80.0	75.0	80.0
132	Diseased	Hungary	Male	71						6.0	6.0	7.0		16.0		13.0				4.0						2.0	5.0	4.0				4.0		80.0	80.0	90.0
800	Diseased	Spain	Female	65								4.0				1.0				7.0				14.0				9.0				4.0				100.0
641	Diseased	Spain	Female	71			18,390.0			5.0	6.0			51.0	23.0				9.0			10.0				15.0	14.0				8.0			50.0	80.0	
624	Diseased	Spain	Female	72						6.0				21.0								14.0				5.0				8.0				80.0		
644	Diseased	Spain	Male	70						7.0																								40.0		
625	Diseased	Spain	Male	72						5.0				51.0								11.0				14.0				8.0				40.0		
634	Diseased	Spain	Male	72			15,748.5			5.0	5.0																			3.0				40.0	40.0	
791	Diseased	Spain	Male	72				1953.0				7.0																								100.0
799	Diseased	Spain	Male	79								4.0				4.0				9.0				14.0				9.0				7.0				95.0

Color coding: from orange (worse score) to yellow to green (better).

##### Physical Activity (IPAQ)

We recorded 27 answers about physical activity on the IPAQ scale [26] that partitions physical activity into *low*, *moderate*, and *high* levels. The scale is described in depth in Appendix B.1.1. All participants recorded a median (mean ± SD) numeric score of 8038 (9535 ± 7106). There were 14 answers with a low categorical level of physical activity, one answer with a moderate level, and 12 answers with a high level. Table A3 enumerates the answers and Figure A3 depicts the sub-scores and scores by participant group.

Participant physical activity separated into two groups at the extremes of low and high physical activity. The levels only approximated the numeric scores, as the low categorical scores concentrated in the lower third of numeric scores and the high categorical scores concentrated in the upper third of numeric scores; the middle third included low and high levels of physical activity alike.

The participants from Hungary self-reported increased physical activity as compared to those from Spain, registering a median (mean ± SD) numeric score of 8478 (9738 ± 7370) compared to 6431 (9281 ± 6752) and a median categorical level of high physical activity compared to low physical activity.

Male participants reported increased levels of physical activity, registering a higher median numeric score of 8478 compared to 6820; however, the most active 5 participants contributed to a lower mean (SD) numeric score of 7916 (4038) compared to 11037 (8806) for the females. Woman participants registered higher variability in their self-reported physical activity than men.

Less than half (12/27) of the answers reported physical activity related to the work domain. Only a few (7/27) answers reported cycling as a means of transportation, and they associated with the upper half of numeric scores. The participants from Hungary reported increased physical activity as compared to those from Spain. Male participants reported increased median physical activity, and female participants reported increased mean physical activity.

**Table A3:** Characteristics of PRO Physical Activity (IPAQ).

ID	Health	Wave	Country	Gender	Age	Work Domain Walking Minutes	Work Domain Moderate Minutes	Work Domain Vigorous Minutes	Work Domain Total	Active Transport Domain Walking Minutes	Active Transport Domain Cycling Minutes	Active Transport Domain Total	Domestic Home Domain Moderate Minutes	Domestic Garden Domain Moderate Minutes	Domestic Garden Domain Vigorous Minutes	Domestic Garden Domain Total	Leisure Domain Walking Minutes	Leisure Domain Moderate Minutes	Leisure Domain Vigorous Minutes	Leisure Domain Total	Work Domain Numeric Sub-Score	Leisure Domain Numeric Sub-Score	Active Transport Domain Numeric Sub-Score	Domestic Garden Domain Numeric Sub-Score	Numeric Score	Categorical Score
420	Healthy	1	Hungary	Female	71	0	0	0	0	90	0	90	60	0	0	60	30	0	0	30	0	99	297	180	576	0
791	Diseased	3	Spain	Male	72	0	0	0	0	210	0	210	420	0	0	420	0	0	0	0	0	0	693	1260	1953	0
419	Healthy	2	Hungary	Male	95	0	0	0	0	90	0	90	70	105	150	325	80	0	0	80	0	264	297	1455	2016	0
576	Healthy	2	Hungary	Male	60	20	360	70	450	20	0	20	10	10	0	20	20	0	0	20	2066	66	66	70	2268	1
215	Healthy	1	Hungary	Female	87	0	0	0	0	360	0	360	10	10	0	20	360	0	0	360	0	1188	1188	70	2446	0
795	Healthy	3	Spain	Female	72	0	0	0	0	140	0	140	420	0	0	420	360	0	0	360	0	1188	462	1260	2910	0
420	Healthy	2	Hungary	Female	71	0	0	0	0	30	0	30	920	0	0	920	30	0	0	30	0	99	99	2760	2958	0
617	Healthy	2	Spain	Female	69	0	0	0	0	280	0	280	210	210	0	420	240	0	0	240	0	792	924	1470	3186	0
620	Healthy	2	Spain	Female	69	0	0	0	0	273	0	273	360	61	0	421	315	0	0	315	0	1039	900	1324	3264	0
796	Healthy	3	Spain	Male	74	0	0	0	0	210	0	210	210	210	150	570	630	210	0	840	0	2919	693	2295	5907	0
638	Healthy	2	Spain	Female	71	0	0	0	0	210	0	210	420	840	0	1260	300	0	0	300	0	990	693	4620	6303	0
793	Healthy	3	Spain	Male	68	140	70	65	275	210	0	210	105	420	300	825	0	0	120	120	1262	960	693	3645	6560	2
169	Diseased	2	Hungary	Female	69	0	0	0	0	30	0	30	60	1680	0	1740	30	0	30	60	0	339	99	6900	7338	2
170	Healthy	2	Hungary	Male	70	540	350	210	1100	280	60	340	80	40	150	270	75	105	0	180	4862	667	1284	1225	8038	2
212	Healthy	1	Hungary	Male	72	0	0	0	0	360	360	720	180	180	0	360	300	240	240	780	0	3870	3348	1260	8478	0
535	Healthy	3	Hungary	Male	69	180	0	0	180	630	0	630	0	360	0	360	630	630	0	1260	594	4599	2079	1440	8712	2
212	Healthy	2	Hungary	Male	72	0	0	0	0	375	720	1095	0	60	0	60	120	0	450	570	0	3996	5557	240	9793	0
170	Healthy	3	Hungary	Male	70	210	420	300	930	350	200	550	140	140	300	580	100	0	0	100	4773	330	2355	2630	10,088	2
790	Healthy	3	Spain	Male	66	0	0	0	0	840	0	840	630	0	0	630	630	840	0	1470	0	5439	2772	1890	10,101	0
636	Healthy	2	Spain	Male	68	240	40	180	460	840	0	840	360	1440	60	1860	280	0	0	280	2392	924	2772	7170	13,258	2
634	Diseased	2	Spain	Male	72	840	840	105	1785	840	450	1290	120	180	30	330	315	225	15	555	6972	2059	5472	1245	15,748	2
641	Diseased	2	Spain	Female	71	2940	0	0	2940	280	0	280	840	840	0	1680	280	240	0	520	9702	1884	924	5880	18,390	2
133	Healthy	2	Hungary	Female	71	630	840	450	1920	630	240	870	420	420	240	1080	420	240	0	660	9039	2346	3519	4260	19,164	2
133	Healthy	3	Hungary	Female	71	540	1050	520	2110	840	0	840	420	360	171	951	420	150	90	660	10,142	2706	2772	3642	19,262	2
169	Diseased	3	Hungary	Female	69	420	420	840	1680	120	120	240	1260	1260	360	2880	0	0	0	0	9786	0	1116	10,800	21,702	2
569	Healthy	2	Hungary	Female	67	630	360	0	990	770	0	770	490	770	550	1810	910	550	550	2010	3519	9603	2541	7575	23,238	2
643	Healthy	2	Spain	Female	67	0	0	0	0	840	0	840	1470	1470	0	2940	1470	1470	0	2940	0	10,731	2772	10,290	23,793	0
Median: Healthy						0.0	0.0	0.0	0.0	315.0	0.0	350.0	210.0	160.0	0.0	495.5	300.0	0.0	0.0	337.5	0.0	1113.5	1236.0	1680.0	7299.0	0.0
Median: Diseased						420.0	0.0	0.0	1680.0	210.0	0.0	240.0	420.0	840.0	0.0	1680.0	30.0	0.0	0.0	60.0	6972.0	339.0	924.0	5880.0	15,748.0	2.0
Median: Spain						0.0	0.0	0.0	0.0	276.5	0.0	276.5	390.0	210.0	0.0	600.0	307.5	0.0	0.0	337.5	0.0	1113.5	912.0	2092.5	6431.5	0.0
Median: Hungary						20.0	0.0	0.0	180.0	350.0	0.0	360.0	80.0	140.0	0.0	360.0	100.0	0.0	0.0	180.0	594.0	667.0	1284.0	1455.0	8478.0	2.0
Median: Female						0.0	0.0	0.0	0.0	276.5	0.0	276.5	420.0	390.0	0.0	1015.5	307.5	0.0	0.0	337.5	0.0	1113.5	924.0	3951.0	6820.5	0.0
Median: Male						20.0	0.0	0.0	180.0	350.0	0.0	550.0	120.0	140.0	30.0	360.0	120.0	0.0	0.0	280.0	594.0	960.0	2079.0	1440.0	8478.0	1.0
Median: All						0.0	0.0	0.0	0.0	280.0	0.0	280.0	210.0	180.0	0.0	570.0	280.0	0.0	0.0	315.0	0.0	1039.0	1116.0	1890.0	8038.0	0.0
Mean: Healthy						142.2	158.6	81.5	382.5	394.0	71.8	465.8	317.5	323.0	94.1	734.6	350.9	201.5	65.9	618.4	1756.7	2491.5	1731.0	2762.3	8741.7	0.7
Mean: Diseased						840.0	252.0	189.0	1281.0	296.0	114.0	410.0	540.0	792.0	78.0	1410.0	125.0	93.0	9.0	227.0	5292.0	856.4	1660.8	5217.0	13,026.2	1.6
Mean: Spain						346.6	79.1	29.1	455.0	431.0	37.5	468.5	463.7	472.5	45.0	981.3	401.6	248.7	11.2	661.6	1694.0	2410.4	1647.5	3529.0	9281.0	0.6
Mean: Hungary						211.3	253.3	159.3	624.0	331.6	113.3	445.0	274.6	359.6	128.0	762.4	235.0	127.6	90.6	453.3	2985.4	2011.4	1774.4	2967.1	9738.4	1.1
Mean: Female						368.5	190.7	129.2	688.5	349.5	25.7	375.2	525.7	565.7	94.3	1185.8	368.9	189.2	47.8	606.0	3013.4	2357.4	1307.5	4359.3	11,037.8	0.8
Mean: Male						166.9	160.0	71.5	398.4	404.2	137.6	541.9	178.8	241.9	87.6	508.4	244.6	173.0	63.4	481.1	1763.1	2007.1	2160.0	1986.5	7916.9	1.0
Mean: All						271.4	175.9	101.4	548.8	375.8	79.6	455.4	358.7	409.8	91.1	859.7	309.0	181.4	55.3	545.9	2411.4	2188.7	1718.0	3216.8	9535.1	0.9
SD: Healthy						222.1	287.5	151.0	621.4	279.3	169.9	329.1	340.9	426.9	142.3	698.2	339.5	360.8	149.1	712.8	2918.6	2874.2	1384.6	2603.3	6816.5	0.9
SD: Diseased						1095.2	336.0	328.0	1135.5	284.7	174.3	448.2	453.7	634.2	141.4	947.5	141.7	114.0	12.0	254.7	4437.9	920.5	1936.0	3629.7	7299.5	0.8
SD: Spain						815.6	230.3	55.8	895.9	291.5	124.3	362.0	363.3	521.7	87.8	773.2	370.9	435.5	33.0	780.1	3103.9	2846.0	1445.5	2806.5	6752.6	0.9
SD: Hungary						253.0	324.5	249.8	748.7	267.8	195.0	348.6	362.0	487.6	164.9	799.1	259.3	200.6	172.8	549.7	3742.2	2564.1	1543.9	3086.5	7370.5	0.9
SD: Female						756.0	338.5	259.7	998.0	285.3	66.9	302.6	427.4	563.6	167.8	902.9	380.1	387.2	141.2	810.3	4307.0	3290.5	1077.2	3401.1	8806.6	0.9
SD: Male						246.3	249.2	96.1	534.8	277.5	223.1	385.3	179.7	368.3	108.1	444.1	234.8	259.6	130.7	466.3	2262.5	1849.5	1749.5	1745.9	4038.2	0.9
SD: All						579.4	299.3	200.6	821.7	282.9	171.5	354.8	374.6	506.1	142.2	795.1	324.5	332.0	136.5	670.1	3531.8	2700.3	1502.3	2978.4	7106.2	0.9

Color coding: from orange (worse outcome relative to others) to yellow to green (better outcome).

##### Social Support (MSPSS)

Participants provided 55 answers on the MSPSS scale [27]. Their levels of social support were on a numeric scale from 1.0 to 7.0 corresponding to the categorical *low*, *moderate*, or *high* levels of social support. We describe this scale in Appendix B.1.1. All participants had a median (mean ± SD) numeric score of 5.0 (5.4 ± 0.9). Most answers corresponded to high social support. The levels of social support from separate sources (significant other, family, and friends) were also generally high. No answers reported low social support. Health status, country, and gender did not appear to change the level of social support fundamentally, neither by source nor in general. Table A4 enumerates the answers and Figure A4 depicts the sub-scores and scores by participant group.

Both healthy and diseased participants reported only slightly different levels of social support, as observed from the median (mean ± SD) of 5.0 (5.3 ± 0.9) healthy and 5.0 (5.5 ± 0.9) diseased. Participants with disease reported slightly higher significant other social support, registering mean numeric sub-scores of 5.8 compared to 5.5 for the significant other social support, 5.6 compared to 5.5 for the family social support, and 5.6 compared to 5.4 for the friends social support. Also, the answers had similar variations when comparing groups by health status. We observed no specific questions where the levels of social support differed by health.

Participants from Spain and Hungary self-reported similar levels of social support, registering similar medians (means) of 5.0 (5.4). Participants from Hungary self-reported more stable answers with SD 0.8 vs. 1.0.

**Table A4:** Characteristics of PRO Social Support (MSPSS).

ID	Health	Wave	Country	Gender	Age	Q1: Special Person: Around When in Need	Q2: Special Person: Share Joys and Sorrows	Q3: Family: Tries to Help	Q4: Family: Gives Emotional Help and Support	Q5: Special Person: Real Source of Comfort	Q6: Friends: Try to Help	Q7: Friends: counted on when things go wrong	Q8: Family: Talk About Problems	Q9: Friends: Share My Joys and Sorrows	Q10: Special Person: Cares about Feelings	Q11: Family: Willing to Help Make Decisions	Q12: Friends: talk about problems	Significant Other Numeric Sub-Score	Family Numeric Sub-Score	Friends Numeric Sub-Score	Numeric Score	Categorical Score
700	Healthy	2	Spain	Male	67	5	5	4	2	2	3	4	2	6	4	4	5	4	3	4	3	1
420	Healthy	2	Hungary	Female	71	6	6	5	5	5	4	4	5	4	5	4	5	5	4	4	4	1
212	Healthy	2	Hungary	Male	72	3	5	5	6	5	5	5	5	5	5	5	5	4	5	5	4	1
419	Healthy	2	Hungary	Male	95	5	6	5	6	6	4	4	5	1	6	6	1	5	5	2	4	1
800	Diseased	3	Spain	Female	65	5	6	5	4	5	4	5	4	5	6	4	5	5	4	4	4	1
617	Healthy	1	Spain	Female	69	5	2	5	4	4	5	5	3	4	5	3	5	4	3	4	4	1
617	Healthy	2	Spain	Female	69	4	4	4	4	4	4	4	4	5	4	5	5	4	4	4	4	1
796	Healthy	3	Spain	Male	74	4	4	4	4	4	6	6	4	6	4	4	6	4	4	6	4	1
799	Diseased	3	Spain	Male	79	4	4	4	4	4	4	4	4	4	4	4	4	4	4	4	4	1
575	Healthy	2	Hungary	Female	65	5	6	7	7	3	5	5	6	6	5	3	5	4	5	5	5	1
169	Diseased	1	Hungary	Female	69	6	7	5	3	6	7	7	3	7	6	5	7	6	4	7	5	1
169	Diseased	2	Hungary	Female	69	5	5	6	5	5	6	7	5	7	6	6	7	5	5	6	5	1
169	Diseased	3	Hungary	Female	69	6	7	5	5	4	7	7	3	7	6	4	7	5	4	7	5	1
420	Healthy	1	Hungary	Female	71	6	6	6	5	6	1	5	5	5	6	4	5	6	5	4	5	1
215	Healthy	1	Hungary	Female	87	6	6	6	6	5	5	5	5	5	6	6	5	5	5	5	5	1
576	Healthy	2	Hungary	Male	60	5	5	5	5	5	5	5	5	5	5	5	5	5	5	5	5	1
170	Healthy	2	Hungary	Male	70	5	5	6	5	5	6	6	5	7	5	7	7	5	5	6	5	1
170	Healthy	3	Hungary	Male	70	5	5	6	4	6	6	6	5	6	5	5	6	5	5	6	5	1
212	Healthy	1	Hungary	Male	72	6	6	5	6	5	5	5	6	5	6	5	5	5	5	5	5	1
212	Healthy	3	Hungary	Male	72	5	5	5	5	5	5	5	5	5	5	5	5	5	5	5	5	1
419	Healthy	1	Hungary	Male	95	6	6	5	5	6	5	5	5	5	6	5	5	6	5	5	5	1
640	Healthy	1	Spain	Female	69	1	6	5	5	6	5	6	6	6	6	6	6	4	5	5	5	1
641	Diseased	1	Spain	Female	71	3	6	6	5	6	6	6	5	6	3	6	6	4	5	6	5	1
630	Healthy	1	Spain	Female	74	4	6	5	3	5	6	6	6	6	6	4	6	5	4	6	5	1
411	Healthy	1	Spain	Male	45	1	7	6	6	6	5	6	6	6	7	6	7	5	6	6	5	1
636	Healthy	1	Spain	Male	68	7	5	6	6	7	5	5	6	5	7	5	5	6	5	5	5	1
636	Healthy	2	Spain	Male	68	5	7	5	6	5	5	5	5	5	6	5	5	5	5	5	5	1
793	Healthy	3	Spain	Male	68	6	6	6	6	6	5	5	6	6	6	6	6	6	6	5	5	1
625	Diseased	1	Spain	Male	72	7	7	7	7	7	5	2	7	3	7	7	5	7	7	3	5	1
634	Diseased	1	Spain	Male	72	3	6	6	6	5	5	5	6	5	6	7	5	5	6	5	5	1
634	Diseased	2	Spain	Male	72	6	5	6	5	5	5	5	5	5	5	5	5	5	5	5	5	1
569	Healthy	2	Hungary	Female	67	6	7	7	7	7	5	5	7	5	7	7	5	6	7	5	6	2
569	Healthy	3	Hungary	Female	67	7	7	7	7	7	6	6	7	6	7	6	6	7	6	6	6	2
133	Healthy	1	Hungary	Female	71	7	7	7	7	7	6	6	7	6	7	6	7	7	6	6	6	2
133	Healthy	2	Hungary	Female	71	7	7	7	6	7	6	7	7	6	7	7	7	7	6	6	6	2
133	Healthy	3	Hungary	Female	71	6	7	7	7	7	6	6	7	6	7	6	6	6	6	6	6	2
535	Healthy	3	Hungary	Male	69	7	7	7	7	7	6	6	7	6	6	7	6	6	7	6	6	2
132	Diseased	1	Hungary	Male	71	7	7	7	7	7	6	6	6	5	7	6	5	7	6	5	6	2
132	Diseased	2	Hungary	Male	71	7	7	7	7	7	6	6	6	5	7	7	5	7	6	5	6	2
643	Healthy	2	Spain	Female	67	6	6	6	6	6	6	6	6	6	6	6	6	6	6	6	6	2
798	Healthy	3	Spain	Female	67	6	6	6	6	6	6	6	6	6	6	6	6	6	6	6	6	2
620	Healthy	2	Spain	Female	69	6	6	6	6	6	6	6	6	6	6	6	6	6	6	6	6	2
638	Healthy	1	Spain	Female	71	7	7	7	7	7	6	6	7	6	6	6	6	6	6	6	6	2
638	Healthy	2	Spain	Female	71	6	6	7	6	6	5	5	7	6	7	6	6	6	6	5	6	2
641	Diseased	2	Spain	Female	71	5	6	7	7	6	6	6	6	6	4	7	6	5	6	6	6	2
624	Diseased	1	Spain	Female	72	7	6	7	7	7	6	7	7	7	7	7	7	6	7	6	6	2
648	Healthy	1	Spain	Female	72	6	6	6	6	6	6	6	6	6	6	6	6	6	6	6	6	2
795	Healthy	3	Spain	Female	72	6	6	7	7	6	7	7	7	7	7	7	6	6	7	6	6	2
790	Healthy	3	Spain	Male	66	7	6	7	7	7	6	6	7	6	5	7	6	6	7	6	6	2
569	Healthy	1	Hungary	Female	67	7	7	7	7	7	7	7	7	7	7	7	7	7	7	7	7	2
132	Diseased	3	Hungary	Male	71	7	7	7	7	7	7	7	7	7	7	7	7	7	7	7	7	2
643	Healthy	1	Spain	Female	67	7	7	7	7	7	7	7	7	7	7	7	7	7	7	7	7	2
628	Healthy	1	Spain	Female	70	7	7	7	7	7	7	7	7	7	7	7	7	7	7	7	7	2
644	Diseased	1	Spain	Male	70	7	7	7	7	7	7	7	7	7	7	7	7	7	7	7	7	2
791	Diseased	3	Spain	Male	72	7	7	7	7	7	7	7	7	7	7	7	7	7	7	7	7	2
Median: Healthy						6.0	6.0	6.0	6.0	6.0	5.0	6.0	6.0	6.0	6.0	6.0	6.0	6.0	5.0	6.0	5.0	1.0
Median: Diseased						6.0	6.5	6.5	6.5	6.0	6.0	6.0	6.0	6.0	6.0	6.5	6.0	5.5	6.0	6.0	5.0	1.0
Median: Spain						6.0	6.0	6.0	6.0	6.0	6.0	6.0	6.0	6.0	6.0	6.0	6.0	6.0	6.0	6.0	5.0	1.0
Median: Hungary						6.0	6.0	6.0	6.0	6.0	6.0	6.0	5.0	6.0	6.0	6.0	5.0	6.0	5.0	5.0	5.0	1.0
Median: Female						6.0	6.0	6.0	6.0	6.0	6.0	6.0	6.0	6.0	6.0	6.0	6.0	6.0	6.0	6.0	6.0	2.0
Median: Male						6.0	6.0	6.0	6.0	6.0	5.0	5.0	6.0	5.0	6.0	6.0	5.0	5.0	5.0	5.0	5.0	1.0
Median: All						6.0	6.0	6.0	6.0	6.0	6.0	6.0	6.0	6.0	6.0	6.0	6.0	6.0	6.0	6.0	5.0	1.0
Mean: Healthy						5.5	5.9	5.9	5.7	5.7	5.3	5.5	5.7	5.6	5.9	5.5	5.6	5.5	5.4	5.3	5.3	1.4
Mean: Diseased						5.7	6.2	6.1	5.8	5.9	5.8	5.8	5.5	5.8	5.9	6.0	5.9	5.7	5.6	5.6	5.5	1.4
Mean: Spain						5.3	5.8	5.9	5.6	5.7	5.5	5.6	5.7	5.7	5.8	5.7	5.8	5.4	5.5	5.4	5.3	1.4
Mean: Hungary						5.9	6.2	6.0	5.8	5.8	5.4	5.7	5.6	5.5	6.0	5.6	5.6	5.7	5.4	5.4	5.3	1.4
Mean: Female						5.7	6.1	6.1	5.8	5.8	5.6	5.9	5.8	5.9	6.0	5.6	6.0	5.6	5.5	5.6	5.5	1.5
Mean: Male						5.4	5.8	5.8	5.7	5.7	5.3	5.3	5.5	5.3	5.8	5.7	5.4	5.5	5.5	5.2	5.1	1.2
Mean: All						5.6	6.0	6.0	5.7	5.8	5.5	5.6	5.6	5.6	5.9	5.7	5.7	5.5	5.5	5.4	5.3	1.4
SD: Healthy						1.4	1.0	0.9	1.2	1.1	1.1	0.8	1.1	1.0	0.9	1.1	1.0	0.9	1.0	0.9	0.9	0.4
SD: Diseased						1.3	0.9	0.9	1.3	1.0	0.9	1.3	1.3	1.2	1.2	1.1	1.0	1.0	1.1	1.2	0.9	0.4
SD: Spain						1.6	1.1	1.0	1.3	1.2	0.9	1.1	1.3	0.9	1.1	1.1	0.7	1.0	1.2	1.0	1.0	0.4
SD: Hungary						0.9	0.8	0.8	1.1	1.1	1.2	0.9	1.1	1.2	0.7	1.1	1.2	0.9	0.8	1.0	0.7	0.4
SD: Female						1.3	1.0	0.8	1.2	1.0	1.2	0.8	1.3	0.8	1.0	1.2	0.7	0.9	1.1	0.9	0.8	0.4
SD: Male						1.5	0.9	1.0	1.2	1.2	0.9	1.0	1.1	1.2	1.0	1.0	1.2	1.0	1.0	1.1	0.9	0.4
SD: All						1.4	1.0	0.9	1.2	1.1	1.1	1.0	1.2	1.1	1.0	1.1	1.0	1.0	1.0	1.0	0.9	0.4

Color coding: from orange (worse outcome relative to others) to yellow to green (better outcome).

Men self-reported lower social support than women, as observed in the median (mean ± std) numeric scores of 5.0 (5.2 ± 1.0) vs. 6.0 (5.5 ± 0.8) as well as median categorical score drop from high to moderate. Males self-reported less social support from the friends at means 5.2 vs. 5.6, less social support from the significant other at means 5.5 vs. 5.6, and similar social support from the family at mean 5.5.

##### Anxiety and Depression (GADS)

We measured anxiety and depression through 34 answers on the GADS scale [28]. The scale assesses whether the anxiety and depression are categorized as *absent*, *possible*, *mild*, *moderate*, or *severe* through a numeric score from 0 to 90. It can be consulted in Appendix B.1.1. Participant mean ± SD numeric score was 20.8 ± 18.1. Participants self-reported absent anxiety and depression in 10 answers, possible anxiety and depression in 12 answers, mild in 6 answers, moderate in 4 answers, and severe in 2 answers. Table A5 enumerates the answers and Figure A5 illustrates the scores by participant group.

Most answers corresponding to moderate and severe anxiety and depression originated from participants who self-reported as diseased. Across the items and scores, the participants with disease reported more substantial anxiety and depression than the healthy participants, in particular for questions Q3A and Q7D. The median (mean ± SD) value for Q3A was 3.0 (2.0 ± 1.7) vs. 1.0 (0.9 ± 0.9). The median (mean ± SD) value for Q7D was 4.0 (2.8 ± 1.8) vs. 1.0 (1.3 ± 1.3), different by 2 and 3 levels, respectively. The median categorical scores were also different by one level, from possible to mild anxiety and depression. The answers from healthy participants had less variability than the answers from the participants with disease.

Across multiple items, women reported more anxiety and depression than male participants, yielding numeric scores higher by 8 units, as observed by the median (mean ± SD) scores of 18.0 (23.8 ± 18.8) compared to 11.5 (13.7 ± 13.9). They reported anxiety and depression with higher variability as well.

**Table A5:** Characteristics of PRO Anxiety and Depression (GADS).

ID	Health	Wave	Country	Gender	Age	Q1A: Keyed-up or on Edge	Q2A: Worrying a Lot	Q3A: Irritable	Q4A: Difficulty Relaxing	Q5A: Sleeping Poorly	Q6A: Headaches or Neck Aches	Q7A: Trembling [...] Urine	Q8A: Worried about Your Health	Q9A: Difficulty Falling Asleep	Q1D: Lacking Energy	Q2D: Lost Interest in Things	Q3D: Lost Confidence in Yourself	Q4D: Hopeless	Q5D: Difficulty Concentrating	Q6D: Lost Weight Due to Poor Appetite	Q7D: Waking Early	Q8D: Slowed Down	Q9D: Worse in the Mornings	Numeric Score	Categorical Score
800	Diseased	3	Spain	Female	65	0	1	0	0	0	0	0	0	0	0	0	0	0	0	0	0	0	0	1	0
643	Healthy	1	Spain	Female	67	0	0	0	0	0	1	0	0	0	0	0	0	0	0	0	1	0	0	2	0
796	Healthy	3	Spain	Male	74	0	0	0	0	0	0	0	0	0	0	2	0	0	0	0	0	0	0	2	0
535	Healthy	3	Hungary	Male	69	0	0	1	0	0	1	0	0	0	0	0	0	0	0	0	1	0	0	3	0
628	Healthy	1	Spain	Female	70	0	0	1	0	0	0	0	0	0	1	0	0	0	0	1	0	0	0	3	0
790	Healthy	3	Spain	Male	66	1	1	0	0	0	0	0	0	0	0	0	0	0	0	0	1	0	0	3	0
799	Diseased	3	Spain	Male	79	0	0	0	0	0	0	1	0	0	3	0	0	0	0	0	0	0	0	4	0
648	Healthy	1	Spain	Female	72	0	0	0	0	1	0	1	1	0	1	0	0	0	0	0	0	1	0	5	0
643	Healthy	2	Spain	Female	67	2	2	0	0	0	0	0	0	0	0	0	0	0	0	0	3	0	0	7	0
575	Healthy	2	Hungary	Female	65	1	0	1	0	1	1	0	1	0	0	1	0	0	0	0	1	1	0	8	0
638	Healthy	1	Spain	Female	71	0	1	2	2	0	1	0	0	1	0	1	0	0	0	0	1	1	0	10	1
700	Healthy	2	Spain	Male	67	2	1	1	1	2	0	0	0	2	0	0	0	0	0	0	1	0	0	10	1
569	Healthy	3	Hungary	Female	67	1	1	1	0	2	1	1	0	2	0	0	1	0	0	0	0	0	1	11	1
133	Healthy	3	Hungary	Female	71	1	1	1	0	2	2	0	0	1	1	0	0	0	1	1	0	1	1	13	1
132	Diseased	3	Hungary	Male	71	1	1	1	0	1	1	0	1	0	1	1	1	0	0	0	1	2	1	13	1
419	Healthy	2	Hungary	Male	95	0	0	1	0	0	4	4	0	0	1	1	0	0	0	0	0	2	0	13	1
420	Healthy	2	Hungary	Female	71	1	1	0	0	1	3	0	0	1	0	1	2	0	1	1	0	4	0	16	1
132	Diseased	1	Hungary	Male	71	1	0	0	1	1	1	1	1	1	1	1	1	0	1	1	1	2	1	16	1
133	Healthy	1	Hungary	Female	71	1	0	1	2	1	1	1	0	1	1	2	0	0	1	1	2	2	0	17	1
795	Healthy	3	Spain	Female	72	1	1	0	1	2	0	0	1	2	2	1	0	0	0	4	1	1	0	17	1
569	Healthy	1	Hungary	Female	67	1	1	0	2	4	2	1	0	4	1	1	0	0	0	0	1	0	1	19	1
624	Diseased	1	Spain	Female	72	1	4	3	0	2	0	0	0	2	2	0	0	0	0	0	4	3	0	21	1
170	Healthy	3	Hungary	Male	70	1	2	1	1	2	1	1	1	0	1	1	2	1	1	1	3	2	0	22	2
641	Diseased	2	Spain	Female	71	2	4	3	2	1	2	0	0	0	2	0	0	0	1	0	4	1	1	23	2
640	Healthy	1	Spain	Female	69	1	1	1	1	1	4	2	3	2	3	0	0	0	4	0	1	2	0	26	2
620	Healthy	2	Spain	Female	69	2	2	2	1	3	4	0	2	2	1	2	0	0	1	0	3	4	0	29	2
630	Healthy	1	Spain	Female	74	4	3	2	2	2	2	0	3	1	3	0	0	0	1	2	4	2	0	31	2
215	Healthy	1	Hungary	Female	87	1	2	1	2	4	2	2	1	2	2	2	2	1	1	1	2	3	2	33	2
169	Diseased	3	Hungary	Female	69	4	1	4	3	4	0	1	1	4	4	4	5	1	1	1	4	4	1	47	3
169	Diseased	2	Hungary	Female	69	4	3	3	3	5	0	1	4	5	2	4	3	1	1	1	4	4	3	51	3
641	Diseased	1	Spain	Female	71	4	4	4	4	4	2	4	4	1	3	3	1	1	1	2	3	2	4	51	3
625	Diseased	1	Spain	Male	72	4	4	0	0	4	1	3	4	4	2	4	1	2	2	4	5	4	3	51	3
617	Healthy	2	Spain	Female	69	3	3	4	4	4	4	4	3	4	5	4	4	5	2	0	4	3	1	61	4
169	Diseased	1	Hungary	Female	69	4	1	4	4	5	4	3	3	5	4	4	5	4	3	2	5	5	3	68	4
Median: Healthy						1.0	1.0	1.0	0.0	1.0	1.0	0.0	0.0	1.0	1.0	1.0	0.0	0.0	0.0	0.0	1.0	1.0	0.0	13.0	1.0
Median: Diseased						2.0	1.0	3.0	1.0	2.0	1.0	1.0	1.0	1.0	2.0	1.0	1.0	0.0	1.0	1.0	4.0	2.0	1.0	23.0	2.0
Median: Spain						1.0	1.0	1.0	0.0	1.0	0.0	0.0	0.0	1.0	1.0	0.0	0.0	0.0	0.0	0.0	1.0	1.0	0.0	10.0	1.0
Median: Hungary						1.0	1.0	1.0	1.0	2.0	1.0	1.0	1.0	1.0	1.0	1.0	1.0	0.0	1.0	1.0	1.0	2.0	1.0	16.0	1.0
Median: Female						1.0	1.0	1.0	1.0	2.0	1.0	0.0	0.5	1.0	1.0	1.0	0.0	0.0	1.0	0.0	1.5	1.5	0.0	18.0	1.0
Median: Male						1.0	0.5	0.5	0.0	0.5	1.0	0.5	0.0	0.0	1.0	1.0	0.0	0.0	0.0	0.0	1.0	1.0	0.0	11.5	1.0
Median: All						1.0	1.0	1.0	0.5	1.0	1.0	0.0	0.0	1.0	1.0	1.0	0.0	0.0	0.0	0.0	1.0	1.5	0.0	16.0	1.0
Mean: Healthy						1.0	1.0	0.9	0.8	1.3	1.4	0.7	0.6	1.0	1.0	0.8	0.4	0.3	0.5	0.5	1.3	1.2	0.2	15.6	1.0
Mean: Diseased						2.2	2.0	2.0	1.5	2.4	1.0	1.2	1.6	2.0	2.1	1.9	1.5	0.8	0.9	1.0	2.8	2.4	1.5	31.4	1.9
Mean: Spain						1.4	1.6	1.2	0.9	1.3	1.1	0.7	1.1	1.1	1.4	0.8	0.3	0.4	0.6	0.6	1.8	1.2	0.4	18.7	1.1
Mean: Hungary						1.4	0.9	1.3	1.2	2.2	1.6	1.0	0.8	1.7	1.2	1.5	1.4	0.5	0.7	0.6	1.6	2.1	0.9	23.3	1.4
Mean: Female						1.6	1.5	1.5	1.3	2.0	1.5	0.8	1.1	1.6	1.5	1.2	0.9	0.5	0.7	0.7	2.0	1.8	0.7	23.7	1.4
Mean: Male						1.0	0.9	0.5	0.3	1.0	0.9	1.0	0.7	0.7	0.9	1.0	0.5	0.3	0.4	0.6	1.3	1.2	0.5	13.7	0.9
Mean: All						1.4	1.3	1.2	1.0	1.7	1.3	0.9	1.0	1.3	1.3	1.1	0.8	0.4	0.6	0.6	1.7	1.6	0.6	20.7	1.2
SD: Healthy						0.9	0.9	0.9	1.0	1.3	1.4	1.1	1.0	1.2	1.2	1.0	1.0	1.0	0.9	0.9	1.2	1.2	0.5	13.4	0.9
SD: Diseased						1.6	1.6	1.7	1.6	1.8	1.2	1.3	1.6	2.0	1.1	1.7	1.8	1.1	0.8	1.2	1.8	1.6	1.3	21.6	1.3
SD: Spain						1.4	1.4	1.3	1.2	1.4	1.4	1.3	1.4	1.2	1.4	1.3	0.9	1.1	1.0	1.2	1.6	1.3	1.0	18.1	1.2
SD: Hungary						1.3	0.8	1.2	1.3	1.6	1.2	1.1	1.1	1.8	1.2	1.3	1.6	1.0	0.7	0.5	1.5	1.5	0.9	17.7	1.0
SD: Female						1.4	1.2	1.4	1.4	1.6	1.4	1.2	1.3	1.5	1.4	1.4	1.6	1.2	0.9	0.9	1.6	1.5	1.1	18.8	1.2
SD: Male						1.1	1.2	0.5	0.4	1.2	1.1	1.3	1.1	1.2	0.9	1.1	0.6	0.6	0.6	1.2	1.4	1.3	0.9	13.9	0.9
SD: All						1.3	1.3	1.3	1.3	1.6	1.3	1.2	1.3	1.5	1.3	1.4	1.4	1.1	0.9	1.0	1.6	1.5	1.0	18.1	1.1

Color coding: from orange (worse outcome relative to others) to yellow to green (better outcome).

##### Mediterranean Nutrition (PREDIMED)

Participants self-reported their adherence to the Mediterranean diet by answering the PREDIMED scale [29,30] 23 times. The scale provides categorical scores for *absent*, *medium*, and *high* adherence using a numeric scale from 0 to 14 points, as described in Appendix B.1.1. Participants registered a mean ± SD numeric score of 7.0 ± 2.4. One-third of the answers corresponded to absent adherence to the Mediterranean diet, and two-thirds correspond to a medium adherence. Table A6 enumerates the answers. Figure A6 illustrates the scores by participant group.

A remarkable result is that among the nutrition diets none had high adherence to a Mediterranean diet. The scoring of the PREDIMED scale may explain this fact. It requires at least 13/14 items to be indicative of a Mediterranean diet to categorize the diet as highly adherent, while only 6/14 are necessary for medium adherence. The most adherent two participants only scored 11/14 and were thus categorized with medium adherence.

One question that associated with the numeric and categorical scores is Q1 referring to olive oil as the primary culinary fat. Conversely, questions Q7 on sweet beverage use and Q13 on the preference for small animal meat had only 1/23 and 2/23 answers in the affirmative.

Participants from the healthy and diseased groups reported similar adherence, but higher variability, with means (SD) of 7.1 (2.7) and 6.9 (1.7), respectively.

The participant country of residence much coincided to the numeric score on the Mediterranean nutrition scale. All participants from Spain reported numeric scores of 7 or higher, corresponding to a medium adherence. Only one outlier person from Hungary had a numeric score of 9, and all other participants from Hungary had numeric scores of 7 or less. All participants categorized as having no adherence to the Mediterranean diet were from Hungary. Participants from Spain reported a median (mean ± SD) numeric score of 9.0 (8.8 ± 1.4) compared to 5.5 (5.3 ± 2.0) for Hungary. In general, the answers from the participants from Hungary had higher variance.

The answers from male participants indicated a higher adherence as depicted by the medians (means ± STD) of 8.5 (7.4 ± 2.6) and 7.0 (6.8 ± 2.3) on the numeric score, but also higher variability. However, there were fewer answers from men than women for this scale.

**Table A6:** Characteristics of PRO Mediterranean Nutrition (PREDIMED).

ID	Health	Wave	Country	Gender	Age	Q1: Olive Oil Main Culinary Fat	Q2: Olive Oil Use	Q3: Vegetables Use	Q4: Fruits Use	Q5: Red Meat, Hamburger, or Meat Use	Q6: Butter, Margarine, or Cream Use	Q7: Sweet/Carbonated Beverage Use	Q8: Wine Use	Q9: Legumes Use	Q10: Fish or Shellfish Use	Q11: Commercial Sweets or Pastries	Q12: Nuts Use	Q13: Preference to Small Animal Meat	Q14: Sofrito Use	Numeric Score	Categorical Score
420	Healthy	2	Hungary	Female	71	0	1.0	1.0	2.0	1.0	2.0	0.0	0.0	1.0	0.0	2.0	0.0	1.0	1.0	2	0
215	Healthy	1	Hungary	Female	87	0	0.0	1.0	2.0	1.0	2.0	0.0	0.0	1.0	0.0	2.0	3.0	0.0	2.0	3	0
170	Healthy	3	Hungary	Male	70	0	1.0	1.0	1.0	1.0	2.0	1.0	5.0	1.0	1.0	5.0	3.0	1.0	3.0	3	0
132	Diseased	3	Hungary	Male	71	0	0.0	2.1	1.1	1.0	2.0	0.0	1.0	2.1	1.0	2.0	1.2	1.0	2.0	4	0
575	Healthy	2	Hungary	Female	65	0	0.0	2.0	1.0	2.0	1.0	0.0	3.0	1.0	1.0	4.0	3.0	1.0	4.0	5	0
169	Diseased	2	Hungary	Female	69	0	0.0	1.0	2.0	0.3	1.0	0.0	2.0	0.5	0.5	1.0	0.3	1.0	4.0	5	0
133	Healthy	3	Hungary	Female	71	1	4.0	2.0	2.0	1.0	4.0	0.0	1.0	1.0	0.0	2.0	2.0	1.0	2.0	6	0
419	Healthy	2	Hungary	Male	95	1	1.0	2.0	2.0	1.0	2.0	0.0	1.0	1.0	0.0	0.0	0.0	1.0	2.0	6	0
569	Healthy	3	Hungary	Female	67	1	2.0	3.0	2.0	2.0	2.0	0.0	1.0	2.0	3.0	2.0	6.0	1.0	6.0	7	1
169	Diseased	1	Hungary	Female	69	1	3.0	2.0	1.0	0.2	1.0	0.0	5.0	2.0	0.2	0.0	0.0	1.0	5.0	7	1
169	Diseased	3	Hungary	Female	69	1	1.0	2.0	1.0	0.3	1.0	0.0	7.0	0.5	0.2	0.0	0.5	1.0	1.0	7	1
800	Diseased	3	Spain	Female	65	1	1.0	1.0	3.0	0.0	0.0	0.0	3.0	1.0	2.0	4.0	7.0	1.0	0.0	7	1
617	Healthy	2	Spain	Female	69	1	2.0	2.0	2.0	0.8	0.0	0.0	0.0	1.0	2.4	2.0	5.0	0.0	2.0	7	1
795	Healthy	3	Spain	Female	72	1	4.0	1.0	2.0	0.5	0.0	0.0	1.0	1.0	2.0	0.0	0.7	1.0	0.0	7	1
700	Healthy	2	Spain	Male	67	1	2.0	1.0	2.0	0.0	0.0	0.0	6.0	4.0	2.0	0.0	2.0	1.0	3.0	8	1
535	Healthy	3	Hungary	Male	69	1	2.0	2.0	5.3	0.1	0.0	0.0	3.0	2.0	1.0	3.0	3.0	1.0	3.0	9	1
643	Healthy	2	Spain	Female	67	1	2.0	2.0	4.0	0.5	0.0	0.0	7.0	2.0	2.0	0.0	1.0	1.0	1.0	9	1
641	Diseased	2	Spain	Female	71	1	3.0	2.0	2.0	0.0	0.0	0.0	2.0	1.0	7.0	7.0	4.0	1.0	7.0	9	1
796	Healthy	3	Spain	Male	74	1	3.0	2.0	3.0	0.0	0.0	0.0	1.0	2.0	2.0	0.0	2.0	1.0	3.0	9	1
799	Diseased	3	Spain	Male	79	1	1.0	1.0	3.0	0.0	0.0	0.0	3.0	2.0	2.0	0.0	4.0	1.0	2.0	9	1
620	Healthy	2	Spain	Female	69	1	2.0	2.0	4.0	1.0	0.0	0.0	1.0	2.0	3.0	0.0	4.0	1.0	2.0	10	1
648	Healthy	1	Spain	Female	72	1	2.0	2.0	3.0	0.8	0.0	0.0	2.0	6.0	3.0	7.0	5.0	1.0	5.0	11	1
790	Healthy	3	Spain	Male	66	1	10.0	3.0	4.0	1.0	0.0	0.0	0.0	5.0	1.0	0.0	7.0	1.0	4.0	11	1
Median: Healthy						1.0	2.0	2.0	2.0	1.0	0.0	0.0	1.0	1.5	1.5	2.0	3.0	1.0	2.5	7.0	1.0
Median: Diseased						1.0	1.0	2.0	2.0	0.2	1.0	0.0	3.0	1.0	1.0	1.0	1.2	1.0	2.0	7.0	1.0
Median: Spain						1.0	2.0	2.0	3.0	0.5	0.0	0.0	2.0	2.0	2.0	0.0	4.0	1.0	2.0	9.0	1.0
Median: Hungary						0.5	1.0	2.0	2.0	1.0	2.0	0.0	1.5	1.0	0.3	2.0	1.6	1.0	2.5	5.5	0.0
Median: Female						1.0	2.0	2.0	2.0	0.8	1.0	0.0	2.0	1.0	2.0	2.0	3.0	1.0	2.0	7.0	1.0
Median: Male						1.0	1.5	2.0	2.5	0.5	0.0	0.0	2.0	2.0	1.0	0.0	2.5	1.0	3.0	8.5	1.0
Median: All						1.0	2.0	2.0	2.0	0.8	0.0	0.0	2.0	1.0	1.0	2.0	3.0	1.0	2.0	7.0	1.0
Mean: Healthy						0.7	2.3	1.8	2.5	0.8	0.9	0.0	2.0	2.0	1.4	1.8	2.9	0.8	2.6	7.0	0.6
Mean: Diseased						0.7	1.2	1.5	1.8	0.2	0.7	0.0	3.2	1.3	1.8	2.0	2.4	1.0	3.0	6.8	0.7
Mean: Spain						1.0	2.9	1.7	2.9	0.4	0.0	0.0	2.3	2.4	2.5	1.8	3.7	0.9	2.6	8.8	1.0
Mean: Hungary						0.5	1.2	1.7	1.8	0.9	1.6	0.0	2.4	1.2	0.6	1.9	1.8	0.9	2.9	5.3	0.3
Mean: Female						0.7	1.8	1.7	2.2	0.7	0.9	0.0	2.3	1.5	1.7	2.2	2.7	0.8	2.8	6.8	0.6
Mean: Male						0.7	2.5	1.7	2.6	0.5	0.7	0.1	2.5	2.3	1.2	1.2	2.7	1.0	2.7	7.3	0.6
Mean: All						0.7	2.0	1.7	2.3	0.6	0.8	0.0	2.3	1.8	1.5	1.8	2.7	0.9	2.7	7.0	0.6
SD: Healthy						0.4	2.2	0.6	1.1	0.5	1.1	0.2	2.1	1.5	1.0	2.0	2.0	0.3	1.4	2.7	0.4
SD: Diseased						0.4	1.1	0.5	0.8	0.3	0.6	0.0	1.9	0.6	2.2	2.4	2.4	0.0	2.2	1.7	0.4
SD: Spain						0.0	2.3	0.6	0.7	0.4	0.0	0.0	2.1	1.6	1.4	2.7	2.0	0.2	2.0	1.4	0.0
SD: Hungary						0.5	1.2	0.5	1.1	0.5	0.9	0.2	2.1	0.5	0.8	1.4	1.7	0.2	1.4	1.9	0.4
SD: Female						0.4	1.2	0.5	0.9	0.5	1.1	0.0	2.2	1.2	1.8	2.2	2.2	0.3	2.1	2.3	0.4
SD: Male						0.4	2.9	0.6	1.3	0.4	0.9	0.3	2.0	1.3	0.6	1.7	1.9	0.0	0.6	2.5	0.4
SD: All						0.4	2.0	0.6	1.1	0.5	1.0	0.2	2.1	1.3	1.5	2.1	2.1	0.2	1.7	2.4	0.4

Color coding: from orange (worse outcome relative to others) to yellow to green (better outcome).

##### Nutrition (SelfMNA)

We quantified participant nutrition through 24 self-reported answers on the SelfMNA scale [31]. The scale assesses a categorical nutrition status as *normal*, *at risk of malnutrition*, or *having malnutrition* and a numeric score between 0 and 14, as detailed in depth in Appendix B.1.1. Participants are well-nourished. Participants recorded a mean ± SD numeric score of 12.2 ± 1.7. More than two-thirds of the participants self-reported a healthy amount of nutrition, and the remaining answers reflected a risk of malnutrition. One third obtained the maximum possible numeric score. None of the answers categorized the participant as malnourished. Table A7 depicts the answers and Figure A7 illustrates the scores by participant group.

**Table A7:** Characteristics of PRO Nutrition (SelfMNA).

ID	Health	Wave	Country	Gender	Age	Q1: Food Intake Declined	Q2: Weight Lost	Q3: Described Current Mobility	Q4: Stressed or Severely Ill	Q5: Dementia and/or Severe Sadness	Numeric Score	Categorical Score
795	Healthy	3	Spain	Female	72	1	0	2	2	2	8	1
620	Healthy	2	Spain	Female	69	1	0	2	2	2	9	1
215	Healthy	1	Hungary	Female	87	2	3	1	0	1	10	1
641	Diseased	1	Spain	Female	71	2	3	2	0	2	10	1
569	Healthy	3	Hungary	Female	67	2	0	2	2	2	11	1
628	Healthy	1	Spain	Female	70	1	3	2	2	2	11	1
625	Diseased	1	Spain	Male	72	1	2	2	2	2	11	1
169	Diseased	1	Hungary	Female	69	2	3	2	0	2	12	2
169	Diseased	3	Hungary	Female	69	2	3	2	0	2	12	2
535	Healthy	3	Hungary	Male	69	1	2	2	2	2	12	2
419	Healthy	2	Hungary	Male	95	2	1	2	2	2	12	2
638	Healthy	1	Spain	Female	71	2	3	2	2	2	12	2
630	Healthy	1	Spain	Female	74	1	2	2	2	2	12	2
420	Healthy	2	Hungary	Female	71	1	3	2	2	2	13	2
617	Healthy	2	Spain	Female	69	1	3	2	2	2	13	2
648	Healthy	1	Spain	Female	72	1	3	2	2	2	13	2
133	Healthy	1	Hungary	Female	71	2	3	2	2	2	14	2
800	Diseased	3	Spain	Female	65	2	3	2	2	2	14	2
643	Healthy	1	Spain	Female	67	2	3	2	2	2	14	2
640	Healthy	1	Spain	Female	69	2	3	2	2	2	14	2
624	Diseased	1	Spain	Female	72	2	3	2	2	2	14	2
790	Healthy	3	Spain	Male	66	2	3	2	2	2	14	2
796	Healthy	3	Spain	Male	74	2	3	2	2	2	14	2
799	Diseased	3	Spain	Male	79	2	3	2	2	2	14	2
Median: Healthy						2.0	3.0	2.0	2.0	2.0	12.0	2.0
Median: Diseased						2.0	3.0	2.0	2.0	2.0	12.0	2.0
Median: Spain						2.0	3.0	2.0	2.0	2.0	13.0	2.0
Median: Hungary						2.0	3.0	2.0	2.0	2.0	12.0	2.0
Median: Female						2.0	3.0	2.0	2.0	2.0	12.0	2.0
Median: Male						2.0	2.5	2.0	2.0	2.0	13.0	2.0
Median: All						2.0	3.0	2.0	2.0	2.0	12.0	2.0
Mean: Healthy						1.5	2.2	1.9	1.8	1.9	12.1	1.7
Mean: Diseased						1.8	2.8	2.0	1.1	2.0	12.4	1.7
Mean: Spain						1.5	2.5	2.0	1.8	2.0	12.3	1.6
Mean: Hungary						1.7	2.2	1.8	1.2	1.8	12.0	1.7
Mean: Female						1.6	2.4	1.9	1.5	1.9	12.0	1.6
Mean: Male						1.6	2.3	2.0	2.0	2.0	12.8	1.8
Mean: All						1.6	2.4	1.9	1.6	1.9	12.2	1.7
SD: Healthy						0.4	1.1	0.2	0.4	0.2	1.7	0.4
SD: Diseased						0.3	0.3	0.0	0.9	0.0	1.4	0.4
SD: Spain						0.4	1.0	0.0	0.4	0.0	1.9	0.4
SD: Hungary						0.4	1.0	0.3	0.9	0.3	1.1	0.4
SD: Female						0.4	1.1	0.2	0.8	0.2	1.7	0.4
SD: Male						0.4	0.7	0.0	0.0	0.0	1.2	0.3
SD: All						0.4	1.0	0.1	0.7	0.1	1.7	0.4

Color coding: from orange (worse outcome relative to others) to yellow, to green (better outcome).

The groups of healthy and diseased participants were characterized by similar medians (12.0) and means (12.1 and 12.4), and only slight differences in the standard deviations (1.8 vs. 1.5). Healthy participants self-reported a decline in food intake for question Q1 while participants with disease reported being more stressed and severely ill in question Q4. Participants with disease reported less weight loss in Q2 as well as fewer variable answers across all items and scores except for Q4.

The participants from Spain reported similar levels of nutrition; however alternating ranks between questions: participants from Spain reported more decline in food intake in Q1, less weight loss in Q2, more mobility in Q3, and less stress, illness, dementia, or sadness in Q4 and Q5. Participants from Hungary reported had a more stable numeric score with a standard deviation of 1.11 for Hungary compared to 1.92 for Spain.

Women and men reported similar levels of nutrition, but provided more stable answers within their group, e.g., male standard deviation of 1.21 compared to female standard deviation of 1.79 for the numeric score.

##### Memory (MFE)

Participants reported 36 answers on the MFE scale for memory [32]. The scale classifies memory failures as *absent* or *potential* through a numeric score from 0 to 56. See the description of MFE in Appendix B.1.1. Participants had mean ± SD numeric score of 8.7 ± 4.7. The median and mean numeric scores indicate absent memory failures. One-third of the answers indicate the possibility of memory failures, originating predominantly from female participants from Spain. Table A8 enumerates the answers. Figure A8 illustrates the scores by participant group.

One item whose answers may associate with the numeric score is Q15: Forgetting important details of done things.

The participants self-reported as diseased reported a higher probability of memory failures, as seen in the median (mean ± SD) numeric score of 9 (9.41 ± 4.5) compared to 7 (8.45 ± 4.8) for healthy participants. The ranking for the medians and means for individual items between the healthy and diseased alternate. Examples of questions where the diseased fared worse include Q5 (checking whether something was done), Q6 (forgetting time of events), Q14 (forgetting to do planned things), and Q18 (forgetting to tell somebody something important) as seen from the medians different by 1 out of the maximum two levels as well as the slightly different means. Healthy and diseased participants had similar variability in the numeric scores and alternating ranks of variability within individual questions.

The participants from Hungary may have slightly fewer chances of memory failure, as observed from the medians (means) of 7.5 (7.7) and 8.5 (9.7) different by 1 (2) points. Furthermore, the numeric scores from the participants from Hungary are more stable. Questions Q5 (checking whether something was done) and Q6 (forgetting time of events) indicate the potential memory decline within the subjects from Spain. Question Q8 (being reminded about things) indicates the opposite. Other questions that weigh towards an expected increase in memory failures for the participants from Spain are Q7 (being reminded about things), Q21 (telling someone a story or joke repeatedly), and Q24 (forgetting where things are normally kept).

Men self-reported improved memory numeric scores as compared to women, as seen from the medians (means) of 6 (6.54) and 8 (9.76), respectively. Questions that contribute to this difference are Q6, Q8, and Q24 and against this difference Q5. Males self-reported more stable memory failures, as seen from the SD 3.86 and SD 4.76, respectively.

**Table A8:** Characteristics of PRO Memory (MFE).

ID	Health	Wave	Country	Gender	Age	Q1: Forgetting Objects put	Q2: Failing to Recognise Places	Q3: Finding a Television Story Difficult	Q4: Not Remembering a Change in Daily Routine	Q5: Checking Whether Something Was Done	Q6: Forgetting Time of Events	Q7: Completely Forgetting to Take Things	Q8: Being Reminded about Things	Q9: Reading Anew Already Read Something	Q10: Letting Ramble about Unimportant Things	Q11: Failing to Recognise Close Relatives or Friends	Q12: Having Difficulty Picking up a New Skill	Q13: Finding Word Is “on the tip of the Tongue”	Q14: Forgetting Forgetting to do Planned Things	Q15: Forgetting important details of done things	Q16: Forgetting the Topic of an Ongoing Conversation	Q17: Failing to follow a Story in a Newspaper	Q18: Forgetting to tEll Somebody Something Important	Q19: Forgetting Important Details about Oneself	Q20: Getting Told Details Mixed up and Confused	Q21: Telling Someone a Story or Joke Repeatedly	Q22: Forgetting Details of Things You Do Regularly	Q23: Finding Famous Faces Unfamiliar	Q24: Forgetting Where Things Are Normally Kept	Q25: Getting Lost Where You Have OFTEN been before	Q26: Getting Lost Where You Have Been RARELY before	Q27: Doing Some Routine Thing Twice by Mistake	Q28: Repeating to sOmeone What You Have just Told Them	Numeric Score	Categorical Score
796	Healthy	3	Spain	Male	74	0	0	0	0	0	0	0	0	0	0	0	0	0	0	0	0	0	0	0	0	0	0	0	0	0	0	0	0	0	0
132	Diseased	1	Hungary	Male	71	0	0	0	0	0	0	0	0	0	0	0	0	0	0	0	0	0	0	0	0	0	0	0	0	1	1	0	0	2	0
643	Healthy	2	Spain	Female	67	1	0	0	0	0	0	0	0	0	0	0	0	1	0	0	0	0	0	0	0	0	0	0	1	0	0	0	0	3	0
648	Healthy	1	Spain	Female	72	0	0	0	0	0	0	0	1	0	0	0	0	1	0	0	0	0	0	0	0	1	0	0	0	0	0	0	0	3	0
132	Diseased	3	Hungary	Male	71	0	0	0	0	1	0	0	0	0	0	0	0	0	0	0	0	0	0	0	1	0	0	0	0	1	1	0	0	4	0
132	Diseased	2	Hungary	Male	71	0	0	0	0	0	0	0	0	0	0	0	0	0	1	0	1	0	0	0	0	0	0	0	1	1	1	0	0	5	0
628	Healthy	1	Spain	Female	70	1	0	0	0	0	0	1	0	0	0	0	0	1	0	0	0	0	0	0	0	0	0	0	1	0	1	0	0	5	0
624	Diseased	1	Spain	Female	72	1	0	0	0	0	1	0	0	0	0	0	0	2	0	0	0	0	0	0	0	0	0	0	0	0	1	0	0	5	0
569	Healthy	3	Hungary	Female	67	0	0	0	0	0	0	0	1	0	1	0	1	1	0	0	1	0	0	0	0	0	1	0	0	0	0	0	0	6	0
535	Healthy	3	Hungary	Male	69	1	0	0	0	0	1	0	1	0	0	0	1	1	0	0	0	0	0	0	0	0	0	1	0	0	0	0	0	6	0
170	Healthy	3	Hungary	Male	70	1	1	0	1	0	0	0	1	0	0	0	1	1	0	0	0	0	0	0	0	0	0	0	0	0	0	0	0	6	0
795	Healthy	3	Spain	Female	72	1	0	0	0	1	1	1	0	0	0	0	0	1	0	0	0	0	0	0	0	0	0	0	1	0	0	0	0	6	0
575	Healthy	2	Hungary	Female	65	2	0	0	0	0	0	0	1	0	0	0	1	0	0	0	0	0	1	0	0	1	0	0	1	0	0	0	0	7	0
133	Healthy	3	Hungary	Female	71	0	0	1	0	1	0	1	1	0	0	0	0	1	0	0	0	0	1	0	0	0	0	0	1	0	0	0	0	7	0
638	Healthy	1	Spain	Female	71	1	0	0	0	1	0	0	0	1	0	0	1	1	0	0	0	0	0	0	0	1	0	0	0	0	0	1	0	7	0
790	Healthy	3	Spain	Male	66	0	0	0	0	1	0	1	0	0	0	0	0	1	0	0	0	0	0	0	0	1	0	1	0	0	0	1	1	7	0
700	Healthy	2	Spain	Male	67	1	0	0	0	1	1	0	0	0	0	0	0	1	0	0	0	0	1	0	0	0	0	1	0	0	1	0	0	7	0
569	Healthy	1	Hungary	Female	67	0	0	0	0	0	0	1	0	0	1	0	1	0	1	0	1	0	1	0	0	0	1	0	0	0	0	0	1	8	0
169	Diseased	3	Hungary	Female	69	1	0	0	0	0	1	0	1	1	0	0	0	1	0	0	1	0	0	0	0	0	0	1	1	0	0	0	0	8	0
133	Healthy	1	Hungary	Female	71	0	0	1	0	0	0	1	1	0	0	0	0	1	1	0	0	0	0	0	1	1	0	0	1	0	0	0	0	8	0
420	Healthy	2	Hungary	Female	71	1	0	0	0	1	0	0	1	0	0	0	1	1	0	0	0	0	0	0	1	0	0	0	1	0	1	0	0	8	0
215	Healthy	1	Hungary	Female	87	0	0	0	0	0	1	0	1	0	0	0	0	1	1	1	0	0	1	0	1	0	0	0	0	0	0	0	1	8	0
643	Healthy	1	Spain	Female	67	1	0	0	0	1	1	0	0	0	0	0	0	1	0	0	0	0	0	0	1	1	0	1	0	0	0	0	1	8	0
800	Diseased	3	Spain	Female	65	1	0	0	0	1	1	1	1	0	0	0	0	1	0	0	0	0	1	0	0	0	0	0	1	0	0	1	0	9	0
799	Diseased	3	Spain	Male	79	0	0	0	0	1	1	1	1	0	0	0	0	1	1	0	0	0	1	0	0	1	0	0	0	0	0	1	0	9	0
640	Healthy	1	Spain	Female	69	1	0	1	0	0	0	0	1	0	0	0	1	1	0	1	0	0	0	0	1	1	1	0	1	0	0	0	0	10	0
419	Healthy	2	Hungary	Male	95	2	0	0	1	1	1	1	1	0	1	0	0	0	1	1	0	0	0	0	0	1	0	0	1	0	0	0	0	12	0
169	Diseased	1	Hungary	Female	69	2	0	0	0	0	0	1	1	0	0	1	0	2	1	1	1	0	1	0	0	1	1	0	0	0	0	0	0	13	1
641	Diseased	2	Spain	Female	71	0	0	2	1	1	1	1	1	1	0	0	1	1	1	1	0	0	1	0	0	0	0	0	1	0	0	0	0	14	1
649	Healthy	1	Spain	Female	72	1	0	1	0	0	1	0	0	1	1	0	1	1	1	0	0	0	1	0	0	1	0	2	1	0	1	0	0	14	1
625	Diseased	1	Spain	Male	72	1	0	0	0	1	1	1	1	0	0	0	0	1	1	1	0	0	1	0	0	1	1	1	1	0	0	0	1	14	1
169	Diseased	2	Hungary	Female	69	2	0	1	0	1	1	0	1	1	1	0	0	1	1	1	1	0	1	0	0	1	0	0	0	0	1	0	0	15	1
641	Diseased	1	Spain	Female	71	0	0	0	1	1	1	1	1	1	0	0	1	1	1	1	0	1	1	0	0	0	0	0	1	0	1	1	0	15	1
630	Healthy	1	Spain	Female	74	1	1	0	0	1	1	2	1	1	0	0	1	1	0	0	0	1	0	0	0	1	1	2	1	0	0	1	1	18	1
617	Healthy	2	Spain	Female	69	1	0	0	0	1	1	1	1	0	1	0	1	2	1	1	0	0	1	0	1	1	0	1	2	0	0	1	1	19	1
620	Healthy	2	Spain	Female	69	1	0	0	1	1	1	1	1	1	1	0	1	1	1	0	1	1	1	0	0	0	1	1	1	0	1	1	1	20	1
Median: Healthy						1.0	0.0	0.0	0.0	0.0	0.0	0.0	1.0	0.0	0.0	0.0	0.5	1.0	0.0	0.0	0.0	0.0	0.0	0.0	0.0	0.0	0.0	0.0	1.0	0.0	0.0	0.0	0.0	7.0	0.0
Median: Diseased						0.5	0.0	0.0	0.0	1.0	1.0	0.5	1.0	0.0	0.0	0.0	0.0	1.0	1.0	0.0	0.0	0.0	1.0	0.0	0.0	0.0	0.0	0.0	0.5	0.0	0.5	0.0	0.0	9.0	0.0
Median: Spain						1.0	0.0	0.0	0.0	1.0	1.0	1.0	0.5	0.0	0.0	0.0	0.0	1.0	0.0	0.0	0.0	0.0	0.0	0.0	0.0	0.5	0.0	0.0	1.0	0.0	0.0	0.0	0.0	8.5	0.0
Median: Hungary						0.5	0.0	0.0	0.0	0.0	0.0	0.0	1.0	0.0	0.0	0.0	0.0	1.0	0.0	0.0	0.0	0.0	0.0	0.0	0.0	0.0	0.0	0.0	0.0	0.0	0.0	0.0	0.0	7.5	0.0
Median: Female						1.0	0.0	0.0	0.0	0.0	1.0	0.0	1.0	0.0	0.0	0.0	0.0	1.0	0.0	0.0	0.0	0.0	0.0	0.0	0.0	0.0	0.0	0.0	1.0	0.0	0.0	0.0	0.0	8.0	0.0
Median: Male						0.0	0.0	0.0	0.0	1.0	0.0	0.0	0.0	0.0	0.0	0.0	0.0	1.0	0.0	0.0	0.0	0.0	0.0	0.0	0.0	0.0	0.0	0.0	0.0	0.0	0.0	0.0	0.0	6.0	0.0
Median: All						1.0	0.0	0.0	0.0	0.5	0.5	0.0	1.0	0.0	0.0	0.0	0.0	1.0	0.0	0.0	0.0	0.0	0.0	0.0	0.0	0.0	0.0	0.0	1.0	0.0	0.0	0.0	0.0	8.0	0.0
Mean: Healthy						0.7	0.0	0.1	0.1	0.4	0.4	0.4	0.5	0.1	0.2	0.0	0.5	0.8	0.2	0.1	0.1	0.0	0.3	0.0	0.2	0.4	0.2	0.4	0.5	0.0	0.2	0.2	0.2	8.4	0.1
Mean: Diseased						0.6	0.0	0.2	0.1	0.5	0.6	0.5	0.6	0.3	0.0	0.0	0.1	0.9	0.5	0.4	0.3	0.0	0.5	0.0	0.0	0.3	0.1	0.1	0.5	0.2	0.5	0.2	0.0	9.4	0.4
Mean: Spain						0.7	0.0	0.2	0.1	0.6	0.6	0.6	0.5	0.3	0.1	0.0	0.4	1.0	0.3	0.2	0.0	0.1	0.4	0.0	0.1	0.5	0.2	0.5	0.6	0.0	0.3	0.4	0.3	9.6	0.3
Mean: Hungary						0.7	0.0	0.1	0.1	0.3	0.3	0.3	0.7	0.1	0.2	0.0	0.3	0.6	0.4	0.2	0.3	0.0	0.3	0.0	0.2	0.3	0.1	0.1	0.4	0.1	0.3	0.0	0.1	7.6	0.1
Mean: Female						0.8	0.0	0.2	0.1	0.4	0.5	0.5	0.6	0.3	0.2	0.0	0.4	1.0	0.4	0.2	0.2	0.1	0.4	0.0	0.2	0.4	0.2	0.3	0.6	0.0	0.2	0.2	0.2	9.7	0.3
Mean: Male						0.5	0.0	0.0	0.1	0.5	0.4	0.3	0.4	0.0	0.0	0.0	0.1	0.5	0.3	0.1	0.0	0.0	0.2	0.0	0.0	0.3	0.0	0.3	0.2	0.2	0.3	0.1	0.1	6.5	0.0
Mean: All						0.7	0.0	0.1	0.1	0.5	0.5	0.4	0.6	0.2	0.1	0.0	0.3	0.8	0.3	0.2	0.1	0.0	0.4	0.0	0.1	0.4	0.1	0.3	0.5	0.0	0.3	0.2	0.2	8.7	0.2
SD: Healthy						0.5	0.2	0.3	0.3	0.4	0.4	0.5	0.4	0.3	0.4	0.0	0.5	0.4	0.4	0.3	0.3	0.2	0.4	0.0	0.4	0.4	0.4	0.6	0.5	0.0	0.4	0.4	0.4	4.8	0.3
SD: Diseased						0.7	0.0	0.5	0.3	0.4	0.4	0.5	0.4	0.4	0.2	0.2	0.3	0.6	0.4	0.4	0.4	0.2	0.4	0.0	0.2	0.4	0.3	0.3	0.5	0.4	0.5	0.4	0.2	4.4	0.4
SD: Spain						0.4	0.2	0.5	0.3	0.4	0.4	0.5	0.5	0.4	0.3	0.0	0.4	0.3	0.4	0.4	0.2	0.3	0.4	0.0	0.3	0.5	0.4	0.6	0.5	0.0	0.4	0.4	0.4	5.5	0.4
SD: Hungary						0.8	0.2	0.3	0.3	0.4	0.4	0.4	0.4	0.3	0.4	0.2	0.4	0.5	0.4	0.4	0.4	0.0	0.4	0.0	0.4	0.4	0.3	0.3	0.4	0.3	0.4	0.0	0.3	3.1	0.3
SD: Female						0.6	0.1	0.5	0.3	0.4	0.4	0.5	0.4	0.4	0.4	0.1	0.4	0.4	0.4	0.4	0.4	0.3	0.4	0.0	0.4	0.4	0.4	0.6	0.5	0.0	0.4	0.4	0.4	4.7	0.4
SD: Male						0.6	0.2	0.0	0.3	0.4	0.4	0.4	0.4	0.0	0.2	0.0	0.3	0.4	0.4	0.3	0.2	0.0	0.4	0.0	0.2	0.4	0.2	0.4	0.4	0.4	0.4	0.3	0.3	3.8	0.2
SD: All						0.6	0.2	0.4	0.3	0.5	0.5	0.5	0.4	0.4	0.3	0.1	0.4	0.5	0.4	0.4	0.3	0.2	0.4	0.0	0.3	0.4	0.3	0.5	0.5	0.2	0.4	0.4	0.4	4.7	0.4

Color coding: from orange (worse outcome relative to others) to yellow to green (better outcome).

##### Sleep (PSQI)

The seniors self-reported their sleep quality through 32 answers on the PSQI scale [33]. PSQI assesses sleep quality as *good* or *poor* based on a numeric score from 0 to 21, as described in Appendix B.1.1. Participants recorded a median (mean ± SD) numeric score of 6.0 (6.3 ± 3.9). The median and mean sleep quality situated at the better extremity of poor sleep quality. Two-fifths of the answers corresponded to poor sleep quality. Table A9 enumerates the answers. Figure A9 illustrates the sub-scores and scores by participant group.

The participants with disease self-reported less adequate sleep, as depicted by the median (mean ± SD) of 8.0 (8.6 ± 3.2) compared to 5.0 (5.3 ± 4.3). Participants with disease self-reported less adequate sleep through questions Q5B (trouble sleeping due to waking up in the middle of the night) with a difference between median (mean) answers of 1.5 (0.53) out of 3. Conversely, healthy participants self-reported decreased sleep quality due to using the bathroom in Q5C with a median (mean) difference of 1.0 (0.55) out of 3. The healthy participants provided more stable PROs with a standard deviation for the numeric score of 3.23 as compared to 4.34.

The participants from Hungary reported worse sleep quality with a median (mean ± SD) of 6.0 (7.5 ± 0.2) in Hungary compared to 5.0 (5.5 ± 0.1) in Spain. The difference between the sleep quality for participants in Hungary and Spain is visible in the numeric sub-scores, e.g., subjective sleep quality, latency, duration, efficiency, and disturbance, but not medication. However, the Spanish participants reported more stable PROs.

Women and men reported similar levels of sleep quality with equal medians and means (0.9 and 0.8). Question Q5A: Trouble sleeping: cannot get to sleep influenced the quality of sleep in women, as observed by a difference of over one unit from a maximum of 3 between means. Males provided more stable results with a standard deviation of 2.45 compared to 4.32 for the numeric score. At the extremity of inadequate sleep, the worst six levels of sleep quality correspond to women from both Spain and Hungary.

**Table A9:** Characteristics of PRO Sleep (PSQI).

ID	Health	Wave	Country	Gender	Age	Q1: Time gone to bed at night	Q2: Duration taken to fall asleep	Q3: Time gotten up in the morning	Q4: Duration of actual sleep	Q5A: Trouble sleeping: cannot get to sleep	Q5B: Trouble sleeping: wake up in the middle of the night	Q5C: Trouble sleeping: use the bathroom	Q5D: Trouble sleeping: cannot breathe comfortably	Q5E: Trouble sleeping: cough or snore loudly	Q5F: Trouble sleeping: too cold	Q5G: Trouble sleeping: too hot	Q5H: Trouble sleeping: bad dreams	Q5I: Trouble sleeping: pain	Q5J: Trouble sleeping for other reason(s)	Q6: Frequency of medicine to help you sleep	Q7: Trouble staying awake while driving, eating, or socializing	Q8: Problem with keeping up enthusiasm to get things done	Q9: Sleep quality overall	Subjective Quality Numeric Sub-Score	Latency Numeric Sub-Score	Duration Numeric Sub-Score	Efficiency Numeric Sub-Score	Disturbance Numeric Sub-Score	Medication Numeric Sub-Score	Daytime Dysfunction Numeric Sub-Score	Efficiency Numeric Sub-Score	Numeric Score	Categorical Score
535	Healthy	3	Hungary	Male	69	1410.0	10.0	510.0	480.0	0	1	0	0	0	0	0	0	0	0	0	0	0	0	0	0	0	0	1	0	0	0.9	1	1
643	Healthy	1	Spain	Female	67	1410.0	5.0	420.0	420.0	0	0	0	0	0	0	0	0	0	0	0	0	0	0	0	0	1	0	0	0	0	0.9	1	1
628	Healthy	1	Spain	Female	70	1440.0	10.0	510.0	480.0	0	1	0	0	0	0	0	0	0	0	0	0	0	0	0	0	0	0	1	0	0	0.9	1	1
648	Healthy	1	Spain	Female	72	1500.0	10.0	540.0	480.0	0	1	2	0	0	0	2	0	1	0	0	0	0	0	0	0	0	0	1	0	0	1.0	1	1
620	Healthy	2	Spain	Female	69	1435.0	0.0	480.0	480.0	0	1	0	0	0	0	0	1	1	1	0	0	0	1	1	0	0	0	1	0	0	1.0	2	1
575	Healthy	2	Hungary	Female	65	1395.0	5.0	420.0	420.0	0	1	3	0	0	0	0	3	1	0	0	1	0	0	0	0	1	0	1	0	1	0.9	3	1
634	Diseased	1	Spain	Male	72	1439.0	15.0	420.0	420.0	0	0	0	0	0	0	0	0	0	0	0	0	0	1	1	1	1	0	0	0	0	1.0	3	1
569	Healthy	3	Hungary	Female	67	1385.0	10.0	405.0	420.0	2	2	3	0	2	1	0	1	0	0	0	0	0	1	1	1	1	0	1	0	0	0.9	4	1
133	Healthy	3	Hungary	Female	71	1315.0	30.0	375.0	480.0	2	0	1	0	2	0	0	1	2	0	0	0	0	1	1	2	0	0	1	0	0	1.0	4	1
132	Diseased	3	Hungary	Male	71	1330.0	10.0	370.0	420.0	0	2	0	0	0	0	1	1	2	0	0	1	1	1	1	0	1	0	1	0	1	0.9	4	1
800	Diseased	3	Spain	Female	65	1440.0	15.0	420.0	360.0	0	0	0	0	0	0	0	0	0	0	0	0	0	1	1	1	2	0	0	0	0	0.9	4	1
643	Healthy	2	Spain	Female	67	1380.0	1.0	435.0	420.0	1	0	0	0	0	0	0	0	2	0	0	0	0	0	0	1	1	1	1	0	0	0.8	4	1
790	Healthy	3	Spain	Male	66	1440.0	2.0	420.0	420.0	0	0	0	0	0	0	0	1	0	0	0	1	0	1	1	0	1	0	1	0	1	1.0	4	1
803	Healthy	3	Spain	Female	67	1500.0	15.0	390.0	360.0	0	1	1	0	0	0	1	0	0	0	0	1	1	0	0	1	2	0	1	0	1	1.0	5	0
638	Healthy	1	Spain	Female	71	1470.0	24.0	480.0	420.0	1	0	0	0	1	0	1	0	0	0	1	0	0	1	1	1	1	0	1	1	0	0.9	5	0
133	Healthy	1	Hungary	Female	71	1375.0	30.0	353.0	420.0	2	1	2	0	2	0	2	1	0	0	0	0	1	1	1	2	1	0	1	0	1	1.0	6	0
170	Healthy	3	Hungary	Male	70	1380.0	5.0	360.0	360.0	1	2	2	0	0	0	1	0	0	1	0	1	1	1	1	1	2	0	1	0	1	0.9	6	0
640	Healthy	1	Spain	Female	69	1440.0	20.0	480.0	420.0	2	2	2	0	2	0	3	0	1	0	0	0	0	1	1	2	1	0	2	0	0	0.9	6	0
795	Healthy	3	Spain	Female	72	1500.0	10.0	510.0	360.0	2	2	0	0	0	0	0	0	0	0	0	0	0	1	1	1	2	1	1	0	0	0.8	6	0
420	Healthy	2	Hungary	Female	71	1350.0	20.0	510.0	480.0	2	2	3	1	2	0	0	2	0	0	0	0	1	1	1	2	0	1	2	0	1	0.8	7	0
419	Healthy	2	Hungary	Male	95	1320.0	3.0	330.0	360.0	0	2	3	0	0	0	0	0	2	2	0	2	1	1	1	0	2	1	1	0	2	0.8	7	0
799	Diseased	3	Spain	Male	79	1440.0	30.0	600.0	360.0	0	0	0	0	0	0	0	0	0	0	0	2	0	0	0	1	2	3	0	0	1	0.6	7	0
641	Diseased	2	Spain	Female	71	1380.0	5.0	394.0	360.0	0	1	2	0	0	0	1	0	0	0	3	0	0	1	1	0	2	1	1	3	0	0.8	8	0
624	Diseased	1	Spain	Female	72	1410.0	30.0	465.0	420.0	2	3	3	0	2	0	0	0	2	0	0	1	0	1	1	2	1	1	2	0	1	0.8	8	0
625	Diseased	1	Spain	Male	72	1380.0	15.0	360.0	360.0	0	3	1	0	3	0	0	0	3	0	0	0	0	3	3	1	2	0	2	0	0	0.9	8	0
700	Healthy	2	Spain	Male	67	1410.0	15.0	480.0	360.0	2	3	3	0	0	0	0	0	0	1	0	1	0	1	1	2	2	2	1	0	1	0.7	9	0
617	Healthy	2	Spain	Female	69	1416.0	30.0	517.0	360.0	3	3	2	1	1	1	0	2	1	0	1	0	0	1	1	2	2	2	2	1	0	0.7	10	0
215	Healthy	1	Hungary	Female	87	1380.0	30.0	420.0	250.0	1	1	3	1	0	0	1	1	1	0	1	0	1	2	2	1	3	3	1	1	1	0.5	12	0
630	Healthy	1	Spain	Female	74	1380.0	60.0	390.0	300.0	3	2	2	0	0	0	3	0	0	2	3	0	0	1	1	3	2	2	1	3	0	0.7	12	0
169	Diseased	3	Hungary	Female	69	1380.0	100.0	210.0	150.0	3	3	1	0	3	1	2	2	1	0	0	0	1	2	2	3	3	3	2	0	1	0.6	14	0
169	Diseased	1	Hungary	Female	69	1404.0	60.0	245.0	150.0	3	3	1	0	3	0	2	2	3	3	0	0	1	3	3	3	3	3	2	0	1	0.5	15	0
169	Diseased	2	Hungary	Female	69	1320.0	120.0	300.0	161.0	3	3	1	1	3	3	1	2	3	3	0	0	1	2	2	3	3	3	3	0	1	0.4	15	0
Median: Healthy						1410.0	10.0	427.5	420.0	1.0	1.0	2.0	0.0	0.0	0.0	0.0	0.0	0.0	0.0	0.0	0.0	0.0	1.0	1.0	1.0	1.0	0.0	1.0	0.0	0.0	0.8	5.0	0.0
Median: Diseased						1392.0	22.5	382.0	360.0	0.0	2.5	1.0	0.0	1.0	0.0	0.5	0.0	1.5	0.0	0.0	0.0	0.0	1.0	1.0	1.0	2.0	1.0	1.5	0.0	1.0	0.8	8.0	0.0
Median: Spain						1439.0	15.0	465.0	420.0	0.0	1.0	0.0	0.0	0.0	0.0	0.0	0.0	0.0	0.0	0.0	0.0	0.0	1.0	1.0	1.0	1.0	0.0	1.0	0.0	0.0	0.8	5.0	0.0
Median: Hungary						1380.0	20.0	370.0	420.0	2.0	2.0	2.0	0.0	2.0	0.0	1.0	1.0	1.0	0.0	0.0	0.0	1.0	1.0	1.0	1.0	1.0	0.0	1.0	0.0	1.0	0.8	6.0	0.0
Median: Female						1404.0	20.0	420.0	420.0	2.0	1.0	1.0	0.0	0.0	0.0	0.0	0.0	1.0	0.0	0.0	0.0	0.0	1.0	1.0	1.0	1.0	0.0	1.0	0.0	0.0	0.8	6.0	0.0
Median: Male						1410.0	10.0	420.0	360.0	0.0	2.0	0.0	0.0	0.0	0.0	0.0	0.0	0.0	0.0	0.0	1.0	0.0	1.0	1.0	1.0	2.0	0.0	1.0	0.0	1.0	0.8	6.0	0.0
Median: All						1407.0	15.0	420.0	420.0	1.0	1.0	1.0	0.0	0.0	0.0	0.0	0.0	0.0	0.0	0.0	0.0	0.0	1.0	1.0	1.0	1.0	0.0	1.0	0.0	0.0	0.8	6.0	0.0
Mean: Healthy						1410.5	15.6	442.5	406.8	1.0	1.2	1.4	0.1	0.5	0.0	0.6	0.5	0.5	0.3	0.2	0.3	0.2	0.7	0.7	1.0	1.1	0.5	1.0	0.2	0.4	0.8	5.2	0.4
Mean: Diseased						1392.3	40.0	378.4	316.1	1.1	1.8	0.9	0.1	1.4	0.4	0.7	0.7	1.4	0.6	0.3	0.4	0.4	1.5	1.5	1.5	2.0	1.4	1.3	0.3	0.6	0.7	8.6	0.3
Mean: Spain						1432.1	16.4	458.4	397.8	0.8	1.2	0.9	0.0	0.4	0.0	0.5	0.2	0.5	0.2	0.4	0.3	0.0	0.7	0.7	1.0	1.3	0.6	1.0	0.4	0.2	0.8	5.4	0.4
Mean: Hungary						1364.9	33.3	369.8	350.0	1.4	1.7	1.7	0.2	1.3	0.3	0.7	1.2	1.1	0.6	0.0	0.3	0.6	1.2	1.2	1.3	1.5	1.0	1.3	0.0	0.8	0.7	7.5	0.3
Mean: Female						1408.9	27.8	420.3	372.6	1.3	1.4	1.3	0.1	1.0	0.2	0.8	0.7	0.8	0.3	0.3	0.1	0.3	0.9	0.9	1.3	1.3	0.9	1.2	0.3	0.3	0.8	6.6	0.3
Mean: Male						1394.3	11.6	427.7	393.3	0.3	1.4	1.0	0.0	0.3	0.0	0.2	0.2	0.7	0.4	0.0	0.8	0.3	1.0	1.0	0.6	1.4	0.6	0.8	0.0	0.7	0.8	5.4	0.4
Mean: All						1404.8	23.2	422.4	378.4	1.0	1.4	1.2	0.1	0.8	0.1	0.6	0.6	0.8	0.4	0.2	0.3	0.3	0.9	0.9	1.1	1.4	0.8	1.1	0.2	0.5	0.8	6.3	0.4
SD: Healthy						51.1	13.8	60.2	61.3	1.0	0.9	1.2	0.3	0.8	0.2	0.9	0.8	0.7	0.6	0.6	0.5	0.4	0.5	0.5	0.9	0.8	0.8	0.4	0.6	0.5	0.1	3.2	0.4
SD: Diseased						40.9	38.2	106.3	109.2	1.3	1.3	0.9	0.3	1.4	0.9	0.7	0.9	1.2	1.2	0.9	0.6	0.4	0.9	0.9	1.1	0.7	1.3	1.0	0.9	0.4	0.1	4.3	0.4
SD: Spain						38.5	13.8	59.3	48.5	1.0	1.1	1.0	0.2	0.8	0.2	0.9	0.5	0.8	0.5	0.9	0.5	0.2	0.6	0.6	0.8	0.7	0.9	0.6	0.9	0.4	0.1	3.1	0.4
SD: Hungary						32.3	36.2	84.8	122.8	1.1	0.8	1.1	0.4	1.2	0.8	0.7	0.8	1.0	1.1	0.2	0.6	0.4	0.7	0.7	1.1	1.1	1.3	0.6	0.2	0.5	0.1	4.6	0.4
SD: Female						50.4	29.9	83.2	102.2	1.1	1.0	1.1	0.3	1.1	0.6	1.0	0.9	0.9	0.9	0.8	0.3	0.4	0.7	0.7	1.0	1.0	1.1	0.6	0.8	0.4	0.1	4.3	0.4
SD: Male						43.1	8.0	82.5	41.0	0.6	1.1	1.2	0.0	0.9	0.0	0.4	0.4	1.1	0.6	0.0	0.7	0.4	0.8	0.8	0.6	0.6	1.0	0.5	0.0	0.6	0.1	2.4	0.4
SD: All						48.9	26.7	83.1	89.8	1.1	1.0	1.1	0.3	1.1	0.5	0.9	0.8	1.0	0.8	0.7	0.5	0.4	0.7	0.7	1.0	0.9	1.1	0.6	0.7	0.5	0.1	3.9	0.4

Color coding: from orange (worse outcome relative to others) to yellow to green (better outcome).

##### Health-Related Quality of Life (EQ-5D-3L)

Participants provided 30 answers about their quality of life on the EQ-5D-3L scale [34]. The scale provides 3 severity levels for five facets of life quality, *no problem*, *some problems*, and *extreme problems* as well as a 0–100 numeric score for the health status on the day of the administration, as detailed in Appendix B.1.1. Half of the answers report a health score of 90 or above. Five answers reported a health score of 75 or below, and five answers reported a health score of 100. Table A10 shows the answers and Figure A10 illustrates the sub-scores and scores by participant group.

The mean ± SD perceived health is at 84.96 ± 13.8 across all participants. The means ± SD for the five domains are as follows: 1.2 ± 0.4 for mobility, 1.0 ± 0.0 for self-care, 1.1 ± 0.3 for usual activities, 1.5 ± 0.6 for pain/discomfort, and 1.2 ± 0.4 for depression/anxiety. None of the participants self-reported quality of life issues due to self-care impediments.

The healthy and diseased participants report similar quality of life in the mobility, self-care, and usual activities. However, the participants with disease report worse pain/discomfort and depression/anxiety. Furthermore, the participants with disease report a mean health score of only 77.27 as compared to the 89.42 for the healthy. The participants with disease also self-report less stable answers, e.g., SD for the health score of 16.97 as compared to the SD of 8.95 of the healthy.

Participants from Spain self-reported a slightly improved health than those from Hungary. The participants from Spain reported a median health score of 90 compared to 85 for those from Hungary. However, the mean health scores are similar: 86.84 and 83.52, respectively. The participants from Hungary participants provided more stable health score, but more varied depression/anxiety responses than the participants from Spain.

Female participants report similar health as compared to male participants, with a median health score of 85 compared to 90, but a mean of 85.42 compared to 83.88. Women self-report experiencing slightly less mobility, usual activities, and depression/anxiety.

**Table A10:** Characteristics of PRO Health-Related Quality of Life (EQ-5D-3L).

ID	Health	Wave	Country	Gender	Age	Q1: Mobility	Q2: Self-Care	Q3: Usual Activities	Q4: Pain/Discomfort	Q5: Anxiety/Depression	Health Score
625	Diseased	1	Spain	Male	72	1	1	1	3	1	40
641	Diseased	1	Spain	Female	71	1	1	1	2	2	50
640	Healthy	1	Spain	Female	69	1	1	1	2	1	70
169	Diseased	2	Hungary	Female	69	1	1	2	2	2	75
630	Healthy	1	Spain	Female	74	2	1	2	1	2	75
169	Diseased	1	Hungary	Female	69	2	1	1	2	2	80
169	Diseased	3	Hungary	Female	69	1	1	1	1	2	80
420	Healthy	2	Hungary	Female	71	2	1	1	2	2	80
132	Diseased	1	Hungary	Male	71	1	1	1	1	1	80
641	Diseased	2	Spain	Female	71	1	1	1	2	1	80
624	Diseased	1	Spain	Female	72	2	1	1	2	1	80
648	Healthy	1	Spain	Female	72	2	1	2	2	1	80
796	Healthy	3	Spain	Male	74	1	1	1	1	1	80
575	Healthy	2	Hungary	Female	65	1	1	1	2	1	85
170	Healthy	3	Hungary	Male	70	1	1	1	2	2	85
132	Diseased	3	Hungary	Male	71	1	1	1	2	1	90
212	Healthy	1	Hungary	Male	72	1	1	2	1	1	90
643	Healthy	2	Spain	Female	67	1	1	1	1	1	90
617	Healthy	2	Spain	Female	69	1	1	1	1	1	90
795	Healthy	3	Spain	Female	72	1	1	1	1	1	90
569	Healthy	3	Hungary	Female	67	1	1	1	2	1	95
133	Healthy	1	Hungary	Female	71	1	1	1	1	1	95
419	Healthy	2	Hungary	Male	95	1	1	1	1	1	95
799	Diseased	3	Spain	Male	79	1	1	1	1	1	95
133	Healthy	3	Hungary	Female	71	1	1	1	1	1	99
800	Diseased	3	Spain	Female	65	1	1	1	1	1	100
643	Healthy	1	Spain	Female	67	1	1	1	1	1	100
628	Healthy	1	Spain	Female	70	1	1	1	1	1	100
638	Healthy	1	Spain	Female	71	1	1	1	1	1	100
790	Healthy	3	Spain	Male	66	1	1	1	1	1	100
Median: Healthy						1.0	1.0	1.0	1.0	1.0	90.0
Median: Diseased						1.0	1.0	1.0	2.0	1.0	80.0
Median: Spain						1.0	1.0	1.0	1.0	1.0	90.0
Median: Hungary						1.0	1.0	1.0	2.0	1.0	85.0
Median: Female						1.0	1.0	1.0	1.0	1.0	85.0
Median: Male						1.0	1.0	1.0	1.0	1.0	90.0
Median: All						1.0	1.0	1.0	1.0	1.0	87.5
Mean: Healthy						1.1	1.0	1.1	1.3	1.1	89.4
Mean: Diseased						1.1	1.0	1.0	1.7	1.3	77.2
Mean: Spain						1.1	1.0	1.1	1.4	1.1	83.5
Mean: Hungary						1.1	1.0	1.1	1.5	1.3	86.8
Mean: Female						1.2	1.0	1.1	1.4	1.2	85.4
Mean: Male						1.0	1.0	1.1	1.4	1.1	83.8
Mean: All						1.1	1.0	1.1	1.4	1.2	84.9
SD: Healthy						0.3	0.0	0.3	0.4	0.3	8.9
SD: Diseased						0.3	0.0	0.2	0.6	0.4	16.9
SD: Spain						0.3	0.0	0.3	0.5	0.3	17.0
SD: Hungary						0.3	0.0	0.3	0.4	0.4	7.3
SD: Female						0.4	0.0	0.3	0.4	0.4	12.2
SD: Male						0.0	0.0	0.3	0.6	0.3	16.7
SD: All						0.3	0.0	0.3	0.5	0.4	13.8

Color coding: from orange (worse outcome relative to others) to yellow to reen (better outcome).

#### Appendix C.1.2. Technology-Reported Outcomes (Fitbit)

We overview the TechROs by first assessing the data quality. Table A11 depicts the total compliance (as the number of days including TechROs) as well as the intraday compliance (as the number of valid days). Figure A11 depicts participant compliance in days (all monitored and valid) for each participant group. Figure A12 illustrates participant compliance by outcome. Figure A13, Figure A14, Figure A15 show participant compliance by health, country, and gender groups, respectively.

While participants wore the devices for a median (mean) of 224 (295) days, Fitbit reported TechROs for different durations. Energy expenditure, steps, and heart rate appeared in the majority of days, with their medians (means ± SD) at 224, 204, and 128 (295 ± 238, 276 ± 236, and 230 ± 214) days. The sedentary, light, fair, and vigorous physical activity durations appeared in decreasing durations, with medians (means ± SD) at 136, 136, 91, and 79 days (219 ± 203, 219 ± 202, 165 ± 171, and 160 ± 168 days). Sleep monitoring recorded a median (mean ± SD) of 130 (198 ± 194) days. Cumulative TechROs such as sedentary+light recorded durations corresponding to at most the minimum of their constituents.

**Table A11:** Days of Fitbit (TechRO) data for seniors with at least one PRO (N=32 participants).

PID	Health	Country	Gender	Age	Monitored Days	Energy	Steps	Heart Rate	Sedentary	Sedentary+Light	Light	Light+Fair	Fair	Fair+Very	Very	Active	Sleep	Valid Days
502	Diseased	Hungary	Female	63	231	231	80	73	50	22	49	15	15	9	9	9	8	0
649	Healthy	Spain	Female	72	135	135	125	0	0	0	0	0	0	0	0	0	0	0
630	Healthy	Spain	Female	74	23	23	13	7	7	7	7	4	4	4	4	4	7	1
799	Diseased	Spain	Male	79	34	34	20	15	16	13	15	6	6	6	6	6	13	5
648	Healthy	Spain	Female	72	35	35	24	21	22	19	22	14	14	12	12	12	18	11
791	Diseased	Spain	Male	72	74	74	67	64	56	54	56	12	12	12	12	12	54	11
800	Diseased	Spain	Female	65	43	43	33	22	22	20	22	17	17	17	17	17	19	12
798	Healthy	Spain	Female	67	47	47	39	35	36	35	36	31	31	31	31	31	34	20
796	Healthy	Spain	Male	74	77	77	63	46	49	48	49	48	48	48	48	48	25	20
575	Healthy	Hungary	Female	65	69	69	61	59	60	58	60	41	41	41	41	41	59	23
795	Healthy	Spain	Female	72	274	274	261	40	38	38	38	32	32	31	31	31	35	26
790	Healthy	Spain	Male	66	79	79	70	67	67	65	67	63	63	63	63	63	30	28
624	Diseased	Spain	Female	72	153	153	143	138	139	130	139	50	50	47	47	47	131	30
420	Healthy	Hungary	Female	71	552	552	446	344	420	233	417	138	138	113	114	113	173	34
644	Diseased	Spain	Male	70	169	169	142	90	61	58	61	53	53	53	53	53	54	37
576	Healthy	Hungary	Male	60	439	439	430	430	430	420	430	119	119	94	95	94	429	49
634	Diseased	Spain	Male	72	237	237	230	75	60	57	60	55	55	55	55	55	56	50
793	Healthy	Spain	Male	68	119	119	107	52	52	52	52	52	52	52	52	52	51	51
641	Diseased	Spain	Female	71	167	167	156	118	134	132	134	122	122	120	121	120	129	63
643	Healthy	Spain	Female	67	217	217	201	186	186	186	186	171	171	159	159	159	185	90
638	Healthy	Spain	Female	71	211	211	208	208	208	207	208	147	147	129	130	129	206	127
212	Healthy	Hungary	Male	72	733	733	698	580	538	465	538	244	244	230	233	230	439	136
170	Healthy	Hungary	Male	70	785	785	777	551	369	353	363	303	303	298	299	298	347	140
169	Diseased	Hungary	Female	69	794	794	778	561	398	360	398	312	312	302	303	302	293	141
625	Diseased	Spain	Male	72	288	288	276	254	254	250	254	221	221	217	217	217	250	141
628	Healthy	Spain	Female	70	303	303	290	286	289	288	289	278	278	273	273	273	276	146
617	Healthy	Spain	Female	69	402	402	395	391	392	392	392	355	355	342	344	342	392	170
569	Healthy	Hungary	Female	67	501	501	498	483	479	475	479	417	417	415	415	415	476	215
133	Healthy	Hungary	Female	71	632	632	623	622	623	622	623	521	521	486	487	486	621	242
419	Healthy	Hungary	Male	95	599	599	594	561	567	563	566	502	502	493	494	493	553	245
640	Healthy	Spain	Female	69	385	385	380	376	377	375	377	346	346	344	345	344	371	295
132	Diseased	Hungary	Male	71	639	639	623	618	622	619	622	613	613	613	613	613	617	301
Median: Healthy					274.0	274.0	261.0	208.0	208.0	207.0	208.0	138.0	138.0	113.0	114.0	113.0	185.0	51.0
Median: Diseased					169.0	169.0	143.0	90.0	61.0	58.0	61.0	53.0	53.0	53.0	53.0	53.0	56.0	37.0
Median: Spain					153.0	153.0	142.0	67.0	60.0	57.0	60.0	52.0	52.0	52.0	52.0	52.0	54.0	30.0
Median: Hungary					599.0	599.0	594.0	551.0	430.0	420.0	430.0	303.0	303.0	298.0	299.0	298.0	429.0	140.0
Median: Female					217.0	217.0	201.0	138.0	139.0	132.0	139.0	122.0	122.0	113.0	114.0	113.0	131.0	34.0
Median: Male					237.0	237.0	230.0	90.0	67.0	65.0	67.0	63.0	63.0	63.0	63.0	63.0	56.0	50.0
Median: All					224.0	224.0	204.5	128.0	136.5	131.0	136.5	91.0	91.0	78.5	79.0	78.5	130.0	49.5
Mean: Healthy					315.0	315.0	300.1	254.5	248.0	233.3	247.5	182.1	182.1	174.1	174.7	174.1	225.0	98.5
Mean: Diseased					257.1	257.1	231.6	184.3	164.7	155.9	164.5	134.1	134.1	131.9	132.0	131.9	147.6	71.9
Mean: Spain					165.3	165.3	154.4	118.6	117.3	115.5	117.3	98.9	98.9	95.9	96.1	95.9	111.2	63.5
Mean: Hungary					543.0	543.0	509.8	443.8	414.1	380.9	413.1	293.1	293.1	281.2	282.0	281.2	365.0	138.7
Mean: Female					272.3	272.3	250.2	208.9	204.2	189.4	204.0	158.4	158.4	151.3	151.7	151.3	180.6	86.6
Mean: Male					328.6	328.6	315.1	261.7	241.6	232.0	241.0	176.2	176.2	171.8	172.3	171.8	224.4	93.3
Mean: All					295.1	295.1	276.5	230.4	219.4	206.7	219.0	165.6	165.6	159.6	160.0	159.6	198.4	89.3
SD: Healthy					238.7	238.7	234.0	216.8	207.8	198.5	207.5	164.9	164.9	160.6	161.0	160.6	199.4	89.8
SD: Diseased					231.9	231.9	235.4	200.9	181.4	179.0	181.6	177.8	177.8	177.2	177.3	177.2	173.8	86.6
SD: Spain					113.4	113.4	114.1	116.9	118.2	118.2	118.2	109.5	109.5	106.7	107.1	106.7	118.9	72.5
SD: Hungary					215.8	215.8	235.1	194.6	188.3	195.7	188.6	193.3	193.3	193.8	193.8	193.8	202.2	98.0
SD: Female					215.0	215.0	212.8	195.0	186.9	179.3	186.8	158.9	158.9	153.8	154.2	153.8	176.5	89.2
SD: Male					264.5	264.5	263.1	235.7	222.7	214.5	222.4	186.7	186.7	185.5	185.7	185.5	215.6	90.0
SD: All					238.0	238.0	236.7	214.1	203.0	195.5	202.8	171.0	171.0	167.7	168.0	167.7	194.5	89.6

Color coding: from orange (fewer days relative to others) to yellow to green (more days).

Concerning total compliance, Fitbit devices were worn by the participants in 295 ± 238 days on average and 50% of participants wore the Fitbit devices in at least 224 days. Healthy participants wore the devices on average 58 days more than participants with disease. Hungarian participants were also significantly more compliant in wearing the devices, by achieving mean 543 (446 more) days with monitored data. From the top 10 compliant, six were Hungarian. Most days were recorded by three Hungarians, and most valid days were recorded by one Hungarian. Men wore the devices for only slightly more extended periods than women.

Regarding intraday compliance, participants wore the devices for more than 23 h for a mean ± SD of 89 ± 89 days while 50% of them wore the devices for at least 49 valid days of 21 h. One third had less than 30 valid days, half had less than 60 days, one person had 90 days, and one third had more than 120 days. The participants with disease were more compliant intraday than the healthy participants, keeping 37 valid days as compared to only 51 by the healthy participants, having a relative ratio to the total days of 4. Participants from Hungary were also more compliant intraday, achieving 140 valid days compared to 30 valid days and 13 ratio to total.

We overview the dataset by depicting in Table A12 the medians of the TechRO variables obtained from the participants’ days over the entire period of monitoring and summary statistics by participant group. The following paragraphs describe each TechRO in depth. Figure 8 and Figure 9 depict the median values for each group across the entire monitoring period.

**Table A12:** Median values of TechROs (Fitbit) across the entire monitoring period (N=32 participants).

PID	Health	Country	Gender	Age	Energy (kcal.)	Steps (count)	Heart Rate (bpm)	Sedentary (min.)	Sedentary+Light (min.)	Light (min.)	Light+Fair (min.)	Fair (min.)	Fair+Very (min.)	Very (min.)	Active (min.)	Sleep (h:min.)
575	Healthy	Hungary	Female	65	1733.0	8835.0	64.0	750.0	1005.5	252.0	266.0	7.0	29.0	23.0	289.0	6:48
569	Healthy	Hungary	Female	67	1753.0	10,038.5	56.0	689.0	931.0	235.0	264.0	17.0	46.0	26.0	295.0	7:42
420	Healthy	Hungary	Female	71	1349.0	3462.0	66.0	1286.0	945.0	120.0	181.5	10.0	19.0	6.0	184.0	9:00
133	Healthy	Hungary	Female	71	2163.0	9856.0	64.0	628.0	894.0	257.0	286.0	26.0	53.0	24.0	316.0	8:18
576	Healthy	Hungary	Male	60	2516.0	2624.5	63.0	829.0	996.5	171.0	194.0	8.0	12.0	3.0	197.0	7:18
170	Healthy	Hungary	Male	70	2585.0	13,882.0	53.0	620.0	929.0	309.0	333.0	15.0	51.5	31.0	375.0	7:42
212	Healthy	Hungary	Male	72	2046.0	3445.0	56.0	1152.5	1203.0	92.0	120.5	8.0	22.0	11.0	133.5	4:18
419	Healthy	Hungary	Male	95	2490.0	5239.0	52.0	704.0	885.0	168.0	206.0	26.0	68.0	37.5	250.0	8:12
643	Healthy	Spain	Female	67	1795.0	9281.0	57.0	603.0	935.5	322.0	362.0	32.0	49.0	15.0	384.0	7:42
798	Healthy	Spain	Female	67	1817.0	9911.0	76.0	691.0	971.0	263.5	309.0	23.0	75.0	42.0	351.0	6:42
640	Healthy	Spain	Female	69	1708.0	8892.5	59.5	705.0	934.0	225.0	248.0	18.0	38.0	20.0	273.0	7:48
617	Healthy	Spain	Female	69	1639.0	8545.0	70.0	691.0	873.5	180.0	207.0	22.0	56.0	33.0	239.0	8:18
628	Healthy	Spain	Female	70	1833.0	8876.0	57.0	583.0	821.0	235.0	310.5	70.0	126.0	40.0	362.0	8:18
638	Healthy	Spain	Female	71	1896.0	7907.5	67.0	728.5	976.0	248.0	274.0	21.0	47.0	18.0	284.0	7:06
648	Healthy	Spain	Female	72	1425.0	6235.0	66.0	778.0	992.0	226.5	244.5	13.0	24.5	8.0	251.5	7:18
649	Healthy	Spain	Female	72	1854.0	7520.0										
795	Healthy	Spain	Female	72	1396.0	5664.0	58.0	764.0	1039.0	265.5	300.0	26.0	48.0	17.0	316.0	6:18
630	Healthy	Spain	Female	74	1320.0	6577.0	57.0	825.0	1008.0	147.0	163.5	6.5	17.5	9.5	171.0	6:54
790	Healthy	Spain	Male	66	2686.0	14123.5	60.0	1106.0	1298.0	205.0	233.0	23.0	79.0	52.0	304.0	3:30
793	Healthy	Spain	Male	68	2536.0	8879.0	64.0	791.5	1086.5	291.0	328.0	35.5	59.0	25.0	367.0	4:48
796	Healthy	Spain	Male	74	2347.0	13989.0	61.0	1113.0	1292.5	175.0	210.5	29.0	97.0	71.5	288.5	8:06
502	Diseased	Hungary	Female	63	1230.0	2171.0	75.0	1327.5	1424.5	96.0	155.0	11.0	14.0	3.0	166.0	1:36
169	Diseased	Hungary	Female	69	2000.5	7659.0	54.0	836.5	994.0	199.0	248.0	24.0	56.0	22.0	284.5	7:06
132	Diseased	Hungary	Male	71	3036.0	11136.0	51.0	605.5	807.0	193.0	231.0	32.0	127.0	96.0	335.0	8:24
800	Diseased	Spain	Female	65	1643.0	9030.0	77.5	739.0	989.5	244.0	284.0	21.0	43.0	19.0	308.0	7:00
641	Diseased	Spain	Female	71	1676.0	10216.0	65.0	718.0	965.5	223.5	274.0	33.0	69.0	31.0	308.0	7:06
624	Diseased	Spain	Female	72	1979.0	5292.0	63.0	730.0	970.0	257.0	279.5	13.0	21.0	7.0	287.0	7:42
644	Diseased	Spain	Male	70	2566.0	7903.5	61.0	781.0	952.0	177.0	197.0	11.0	40.0	27.0	231.0	7:30
625	Diseased	Spain	Male	72	2197.0	10394.5	53.0	589.0	876.0	291.0	320.0	20.0	45.0	22.0	351.0	8:30
634	Diseased	Spain	Male	72	3121.0	12832.5	61.0	794.5	1060.0	232.5	310.0	54.0	141.0	77.0	393.0	4:24
791	Diseased	Spain	Male	72	2397.5	4012.0	62.0	789.0	986.0	185.0	199.0	7.5	12.5	5.0	204.5	7:36
799	Diseased	Spain	Male	79	1682.0	4268.0	49.0	878.0	960.0	140.0	187.5	7.5	13.0	7.0	193.0	8:00
Median: Healthy					1833.0	8835.0	60.5	739.2	973.5	230.7	256.0	21.5	48.5	23.5	288.7	7:30
Median: Diseased					2000.5	7903.5	61.0	781.0	970.0	199.0	248.0	20.0	43.0	22.0	287.0	7:24
Median: Spain					1833.0	8876.0	61.0	751.5	973.5	229.5	274.0	21.5	47.5	21.0	296.2	7:24
Median: Hungary					2046.0	7659.0	56.0	750.0	945.0	193.0	231.0	15.0	46.0	23.0	284.5	7:36
Median: Female					1733.0	8545.0	64.0	729.2	970.5	235.0	270.0	21.0	46.5	19.5	288.0	7:12
Median: Male					2516.0	8879.0	60.0	791.5	986.0	185.0	210.5	20.0	51.5	27.0	288.5	7:30
Median: All					1875.0	8690.0	61.0	750.0	971.0	225.0	248.0	21.0	47.0	22.0	288.5	7:24
Mean: Healthy					1947.0	8275.3	61.3	801.8	1000.8	219.3	252.0	21.8	50.8	25.6	281.5	7:06
Mean: Diseased					2138.9	7719.5	61.0	798.9	998.5	203.4	244.0	21.2	52.8	28.7	278.2	6:48
Mean: Spain					1976.8	8588.0	62.2	769.8	999.3	226.6	262.0	24.3	55.0	27.3	293.3	7:00
Mean: Hungary					2081.9	7122.5	59.4	857.0	1001.3	190.1	225.9	16.7	45.2	25.6	256.8	6:54
Mean: Female					1695.2	7682.5	64.0	781.8	981.6	222.0	258.6	21.8	46.1	20.1	281.6	7:06
Mean: Male					2477.3	8671.4	57.3	827.1	1025.5	202.2	236.1	21.2	59.0	35.7	278.6	6:42
Mean: All					2012.9	8084.2	61.2	800.8	1000.0	213.7	249.2	21.6	51.5	26.7	280.3	7:00
SD: Healthy					423.2	3171.9	5.8	195.5	126.4	59.8	61.2	13.8	27.7	16.5	69.1	1:24
SD: Diseased					569.0	3237.4	8.7	186.8	148.5	52.3	51.7	13.4	42.2	28.9	68.1	1:54
SD: Spain					462.0	2735.1	6.7	135.3	115.4	47.6	53.6	15.2	34.1	19.9	62.4	1:18
SD: Hungary					524.4	3768.4	7.1	257.4	164.0	66.6	58.9	8.4	31.6	24.8	73.3	2:00
SD: Female					246.3	2204.8	6.9	196.6	119.0	54.1	52.0	13.9	25.8	11.0	59.4	1:30
SD: Male					363.2	4196.1	4.9	183.5	150.1	60.7	63.4	13.4	40.8	28.7	79.8	1:42
SD: All					486.9	3205.5	7.0	192.5	134.7	57.8	58.2	13.7	33.6	21.8	68.8	1:36

Color coding: from orange (worse outcome relative to others) to yellow to green (better outcome). Participant 649 only provided energy and steps.

##### Energy Expenditure (Raw Family)

For the energy expenditure Fitbit behavioural marker, participants spent a mean ± SD energy of 2013 ± 487 kcal. 50% participants spent 1896 kcal. or more per day. Table A12 illustrates these results.

Participants with disease consumed 100–200 kcal. more than healthy participants per day, with medians (means) of 2000 and 1825 (2139 and 1951). We observed a similar difference between the participants from Hungary and Spain (difference of means 213 kcal). Men consumed more calories than women, with respective medians (means) of 2516 and 1720 (2477 and 1686), but also with higher variation, with male SD 363 kcal. vs. female 250 kcal.

##### Steps (Raw Family)

For the steps Fitbit behavioural marker, participants were active: they performed a median (mean ± SD) of 8690 (8084 ± 3205) measured steps per day. Table A12 illustrates these results.

Healthy participants performed on average 556 more steps than participants with disease, and with a median difference of 932 steps. Healthy and diseased participants had comparable variabilities in the step counts. Participants from Spain performed on average 1217 more steps than participants from Hungary and the devices measured more consistency. Men performed 1992 more steps on average than women. However, the 50% step counts are similar, partly due to four males who performed more than 12.000 median steps per day.

##### Heart Rate (Raw Family)

For the heart rate behavioural marker measured by Fitbit, the median and (mean ± SD) were 61 (61 ± 7) beats per minute. Table A12 illustrates these results.

Both healthy and diseased participants reported similar heart rate means and medians. Devices owned by participants with disease reported higher variability between daily measures than healthy participants with 8.77 bpm. and 5.81 bpm., respectively. Hungarian participant devices reported a lower median at 56 compared to 61 bpm. On average, men had 3 bpm. less than women.

##### Sedentary Duration (Processed Family)

For the behavioural marker of sedentary duration, the participants recorded 801 ± 192 mean minutes per day. Table A12 illustrates these results.

Participants with disease report more sedentary time than healthy participants, with means of 781 and 739 min, respectively. Participants from Hungary report 88 min more sedentary duration on average with 857 compared to 769; however, they report similar medians. Men also report 242 min. more sedentary time than women, with medians 971 and 729 min, respectively.

##### Light Intensity Physical Activity Duration (Processed Family)

For the duration of physical activity at a light intensity as reported by Fitbit, all participants spend on average 213 ± 57 min per day. Table A12 illustrates these results.

Healthy participants report approximately 20 min more per day with a median (mean) of 230 (219) compared to 199 (203). Participants from Spain also report 30 min more with 229 median min for Spain compared to 193 median min for Hungary. Females are more active in the light intensity spectrum by 20 min than males.

##### Fair Intensity Physical Activity Duration (Processed Family)

For the duration of physical activity at a fair intensity as reported by Fitbit, all participants spend on average 21 ± 13 min per day. Table A12 illustrates these results.

Regardless of their grouping criteria of health status, country, or gender, participants consistently report means and medians in the 16–22 min for the fair intensity physical activity.

##### Vigorous Intensity Physical Activity Duration (Processed Family)

For the duration of physical activity at a vigorous intensity as reported by Fitbit, all participants spend on average 26 ± 21 min. per day. Table A12 illustrates these results.

Regardless of their grouping criteria of health status or country, participants consistently report means and medians in the 19–28 min for the vigorous-intensity physical activity. Men may perform vigorous physical activity for 10–15 min more than women, as observed in their respective medians (means) of 27 (35) and 19 (20), but also with more variability as their standard deviation is 28 compared to 11.

##### Sleep Duration (Processed Family)

For the sleep duration, participants sleep on average 7 ± 1.6 h and 50% of the participants sleep 7 h and 30 min. Table A12 illustrates these results.

The healthy participants sleep on average 18 min more than those with mild disease.

### Appendix C.2. Inferential Analysis (PROs vs. TechROs)

We depict the significant correlations between PROs and TechROs for the questionnaires assessing physical activity (Table A13), social support (Table A14), depression and anxiety (Table A15), Mediterranean nutrition (Table A16), nutrition (Table A17), memory (Table A18), sleep (Table A19), and health-related Quality of Life (Table A20). In all tables of this part, we highlight the significant correlations at $r_{S}$ ≥ 0.5.

**Table A13:** Correlation coefficient (Spearman $r_{S}$) between TechROs from Fitbit (rows) and PROs of Physical Activity on the IPAQ scale (columns).

	PRO		Work walking activity			Work Moderate Activity			Work Vigorous Activity			Work Total Activity			Transport Walking Activity			Transport Bicycle Activity			Transport Total Activity			Domestic Moderate Activity			Garden Moderate Activity			Garden Vigorous Activity			Domestic+Garden Total Activity			Leisure Walking Activity			Leisure Moderate Activity			Leisure Vigorous Activity			Leisure Total Activity		
Family	TechRO	Health	Both	Healthy	Diseased	Both	Healthy	Diseased	Both	Healthy	Diseased	Both	Healthy	Diseased	Both	Healthy	Diseased	Both	Healthy	Diseased	Both	Healthy	Diseased	Both	Healthy	Diseased	Both	Healthy	Diseased	Both	Healthy	Diseased	Both	Healthy	Diseased	Both	Healthy	Diseased	Both	Healthy	Diseased	Both	Healthy	Diseased	Both	Healthy	Diseased
Raw	Energy		0.4	0.3	0.6	0.5	0.4		0.5	0.5		0.4	0.4					0.4		0.5			0.6	$0.4_{-}$	$0.5_{-}$				$0.7_{-}$	0.4	0.5		$0.3_{-}$		$0.7_{-}$			0.8						0.7			0.6
	Steps		0.5	0.5	0.7	0.4		0.5	0.3			0.4	0.5	0.6	0.4	0.5		0.4		0.5	0.4	0.4	0.5	0.4	0.5	$0.6_{-}$		0.4	$0.7_{-}$	0.3	0.5			0.5	$0.6_{-}$	0.4	0.3	0.8	0.6	0.5	0.7			0.6	0.5	0.4	0.5
	Heart rate					$0.4_{-}$		$0.7_{-}$	$0.5_{-}$		$0.7_{-}$	$0.3_{-}$		$0.6_{-}$				$0.3_{-}$	$0.4_{-}$							0.5				$0.4_{-}$		$0.7_{-}$							$0.3_{-}$	$0.5_{-}$		$0.3_{-}$				$0.4_{-}$	
Processed	Sedentary		$0.3_{-}$	$0.5_{-}$		$0.3_{-}$	$0.5_{-}$		$0.3_{-}$	$0.6_{-}$		$0.3_{-}$	$0.5_{-}$		$0.6_{-}$	$0.7_{-}$		$0.4_{-}$			$0.6_{-}$	$0.7_{-}$			$0.5_{-}$	0.6		$0.5_{-}$		$0.3_{-}$	$0.6_{-}$	$0.5_{-}$		$0.5_{-}$		$0.5_{-}$	$0.7_{-}$	$0.6_{-}$	$0.5_{-}$	$0.7_{-}$	$0.6_{-}$	$0.3_{-}$		$0.5_{-}$	$0.5_{-}$	$0.7_{-}$	$0.6_{-}$
	Sedentary+light					$0.3_{-}$		$0.7_{-}$	$0.3_{-}$		$0.5_{-}$	$0.5_{-}$						$0.3_{-}$	$0.4_{-}$							0.6				$0.4_{-}$	$0.4_{-}$	$0.5_{-}$						$0.7_{-}$				$0.3_{-}$		$0.6_{-}$	$0.3_{-}$		$0.8_{-}$
	Light			0.4						0.3			0.4		0.4	0.6						0.4		0.7	0.7		0.5	0.7	0.6		0.5		0.4	0.7		0.5	0.7		0.6	0.7	0.5			$0.5_{-}$	0.4	0.7	
	Light+fair									0.5	$0.5_{-}$					0.5						0.5		0.8	0.6		0.5	0.6				$0.5_{-}$	0.5	0.6			0.6				0.6					0.6	
	Fair			$0.7_{-}$		0.3	$0.8_{-}$	0.8	0.3	$0.6_{-}$	0.6	0.3	$0.8_{-}$		0.3			$0.4_{-}$	$0.6_{-}$	0.7				$0.6_{-}$			$0.6_{-}$					0.7													0.3		
	Fair+vigorous		0.3		0.6	0.4		0.7	0.5		0.7	0.3								0.6				$0.6_{-}$			$0.4_{-}$	$0.6_{-}$				0.7	$0.5_{-}$									0.3		0.8			
	Vigorous		0.3			0.3		0.7	0.4		0.7	0.3		0.6		$0.6_{-}$				0.6				$0.4_{-}$	$0.4_{-}$		$0.5_{-}$	$0.6_{-}$				0.7	$0.6_{-}$	$0.4_{-}$										0.6			
	Active		0.3		0.7	0.3		0.6	0.8			0.3		0.6	0.3	0.5		0.4		0.7	0.4	0.6			0.6			0.6						0.5		0.5	0.6		0.5	0.6	0.6	0.3			0.6	0.6	0.6
	Sleep								0.3									0.5	0.4		0.6	0.6								0.4					$0.6_{-}$	0.3		0.6					$0.5_{-}$	0.5			0.6
CLR PA	Sedentary		$0.5_{-}$		$0.7_{-}$	$0.5_{-}$		$0.8_{-}$	$0.6_{-}$	$0.7_{-}$	$0.7_{-}$	$0.5_{-}$		$0.7_{-}$	$0.4_{-}$			$0.5_{-}$		$0.6_{-}$	$0.3_{-}$	$0.7_{-}$					0.3	0.5		$0.4_{-}$		$0.7_{-}$							$0.5_{-}$	$0.5_{-}$		$0.3_{-}$		$0.8_{-}$	$0.4_{-}$		$0.5_{-}$
	Light			0.5	$0.8_{-}$		0.5			0.5	$0.8_{-}$		0.5		0.5	0.6			0.4		0.5	0.5		0.5	0.5		0.5	0.6				$0.8_{-}$	0.6	0.6			0.4		0.3								
	Fair		0.4		0.6						$0.6_{-}$	0.3			0.3			0.6	0.4						0.5		0.7	0.6		$0.6_{-}$		$0.5_{-}$	0.7												$0.3_{-}$		$0.6_{-}$
	Vigorous		0.3	0.5			0.6			0.6			0.6		0.4	0.4					$0.6_{-}$			0.5	0.6		$0.4_{-}$	$0.6_{-}$	$0.7_{-}$	0.3			$0.4_{-}$		$0.7_{-}$				0.6	$0.5_{-}$		0.3		0.8			
CLR PA+S	Sedentary		$0.5_{-}$			$0.5_{-}$		$0.6_{-}$	$0.5_{-}$		$0.7_{-}$	$0.5_{-}$			$0.4_{-}$	$0.5_{-}$					$0.3_{-}$			0.6			0.6			$0.5_{-}$		$0.8_{-}$	0.6									$0.3_{-}$		$0.7_{-}$	$0.4_{-}$		$0.5_{-}$
	Light							$0.7_{-}$		0.5	$0.7_{-}$	0.4	0.6					0.6	0.5											0.4	0.5	$0.7_{-}$												$0.8_{-}$			
	Fair		0.5	0.4	0.7	0.4	0.4		0.3			0.5	0.4	0.5	$0.6_{-}$	$0.6_{-}$		$0.6_{-}$	$0.7_{-}$					$0.4_{-}$	$0.5_{-}$		$0.3_{-}$			$0.7_{-}$			$0.5_{-}$	$0.5_{-}$		$0.6_{-}$			$0.3_{-}$			$0.6_{-}$			$0.6_{-}$		
	Vigorous		0.5	0.7		0.4	0.6		0.4	0.5		0.4	0.6	0.5	$0.7_{-}$						$0.7_{-}$			$0.5_{-}$	$0.6_{-}$	$0.6_{-}$				0.4	0.7		$0.5_{-}$	$0.4_{-}$	$0.6_{-}$	$0.4_{-}$	$0.4_{-}$		0.5						0.6		
	Sleep			0.5			0.6						0.6	$0.7_{-}$				$0.6_{-}$	$0.7_{-}$							$0.6_{-}$				0.4	0.6																
Raw	Total		1	1	2	1		2	2	1	1		1	2		1				2			2		2	2			2		2	1		1	2			2	1	2	1			2	1		2
Processed	Total			2	2		2	5	2	3	5	1	2	2	1	5		1	1	4	2	4		4	4	2	4	6	1		2	6	3	4	1	3	4	3	3	3	4		1	6	2	4	4
CLR PA	Total		1	2	3	1	2	1	1	3	3	1	2	1	1	1		2		1	2	2		2	3		2	4	1	1		3	2	1	1				2	2				2			2
CLR PA+S	Total		3	2	1	1	2	2	1	2	2	2	3	3	2	2		3	3		1			2	2	2	1			2	3	2	3	1	1	1			1			1		2	2		1
All Families	Total		5	7	8	3	6	10	6	9	11	4	8	8	4	9		6	4	7	5	6	2	8	11	6	7	10	4	3	7	12	8	7	5	4	4	5	7	7	5	1	1	12	5	4	9

Color coding: from orange (weaker correlation/fewer total correlations) to yellow to green (stronger correlation/more total correlations). Only significant correlations are shown. Only coefficients of 0.5 or above are highlighted.

**Table A14:** Correlation coefficient (Spearman $r_{S}$) between TechROs from Fitbit (rows) and PROs of Social Support on the MSPSS scale (columns).

	PRO		Q1: Special person: around when in need			Q2: Special person: share joys and sorrows			Q3: Family: tries to help			Q4: Family: gives emotional help and support			Q5: Special person: real source of comfort			Q6: Friends: try to help			Q7: Friends: counted on when things go wrong			Q8: Family: talk about problems			Q9: Friends: share my joys and sorrows			Q10: Special person: cares about feelings			Q11: Family: willing to help make decisions			Q12: Friends: talk about problems			Significant other numeric sub-score			Family numeric sub-score			Friends numeric sub-score			Categorical score			Numeric score		
Family	TechRO	Health	Both	Healthy	Diseased	Both	Healthy	Diseased	Both	Healthy	Diseased	Both	Healthy	Diseased	Both	Healthy	Diseased	Both	Healthy	Diseased	Both	Healthy	Diseased	Both	Healthy	Diseased	Both	Healthy	Diseased	Both	Healthy	Diseased	Both	Healthy	Diseased	Both	Healthy	Diseased	Both	Healthy	Diseased	Both	Healthy	Diseased	Both	Healthy	Diseased	Both	Healthy	Diseased	Both	Healthy	Diseased
Raw	Energy																																								0.5												
	Steps		0.4	0.4			0.4		0.6	0.6			0.3		0.4	0.5		0.5	0.6	$0.5_{-}$	0.5	0.6	$0.8_{-}$		0.4		0.4	0.6	$0.7_{-}$		0.4		0.5	0.5		0.4	0.7	$0.8_{-}$	0.3	0.4		0.4	0.4		0.5	0.6	$0.6_{-}$	0.4	0.5		0.5	0.5	
	Heart rate							$0.5_{-}$										$0.4_{-}$			$0.4_{-}$																											0.3					
Processed	Sedentary		$0.5_{-}$	$0.4_{-}$	$0.6_{-}$	$0.5_{-}$	$0.5_{-}$	$0.5_{-}$	$0.5_{-}$	$0.5_{-}$	$0.7_{-}$	$0.5_{-}$	$0.4_{-}$	$0.8_{-}$	$0.6_{-}$	$0.5_{-}$	$0.7_{-}$	$0.6_{-}$	$0.6_{-}$		$0.4_{-}$	$0.5_{-}$		$0.4_{-}$	$0.4_{-}$		$0.4_{-}$	$0.6_{-}$	0.6	$0.4_{-}$	$0.5_{-}$		$0.7_{-}$	$0.7_{-}$		$0.3_{-}$	$0.6_{-}$	0.6	$0.5_{-}$	$0.5_{-}$	$0.7_{-}$	$0.6_{-}$	$0.5_{-}$		$0.4_{-}$	$0.5_{-}$		$0.6_{-}$	$0.5_{-}$		$0.6_{-}$	$0.5_{-}$	
	Sedentary+light				$0.5_{-}$	$0.3_{-}$	$0.3_{-}$	$0.5_{-}$	$0.3_{-}$						$0.5_{-}$	$0.3_{-}$														$0.3_{-}$	$0.3_{-}$	$0.6_{-}$	$0.5_{-}$						$0.3_{-}$		$0.6_{-}$							$0.4_{-}$					
	Light		0.4	0.5		0.4	0.5	$0.5_{-}$	0.5	0.6		0.4	0.6		0.5	0.6		0.6	0.7	$0.7_{-}$	0.6	0.7	$0.6_{-}$	0.6	0.6		0.6	0.8		0.5	0.5		0.6	0.7		0.6	0.8		0.4	0.6		0.6	0.6		0.7	0.7	$0.5_{-}$	0.6	0.7		0.6	0.7	$0.6_{-}$
	Light+fair		0.3	0.5	$0.6_{-}$	0.4	0.5	$0.7_{-}$	0.5	0.5			0.6			0.5		0.6	0.7	$0.7_{-}$	0.6	0.7	$0.6_{-}$	0.6	0.6		0.7	0.7			0.6	$0.5_{-}$	0.5	0.6		0.7	0.7		0.4	0.6	$0.5_{-}$	0.6	0.7		0.7	0.7		0.7	0.7		0.6	0.8	$0.6_{-}$
	Fair			$0.9_{-}$			$0.9_{-}$		$0.3_{-}$	$0.8_{-}$			$0.7_{-}$			$0.9_{-}$			$0.7_{-}$			$0.7_{-}$		$0.6_{-}$	$0.8_{-}$			0.4		$0.4_{-}$	$0.8_{-}$			$0.6_{-}$			$0.7_{-}$			$0.9_{-}$			$0.6_{-}$			0.4		$0.4_{-}$	$0.7_{-}$			$0.7_{-}$	
	Fair+vigorous			$0.6_{-}$			$0.7_{-}$			$0.6_{-}$						$0.6_{-}$			$0.7_{-}$			$0.6_{-}$		$0.4_{-}$	$0.5_{-}$			$0.6_{-}$		$0.3_{-}$	$0.7_{-}$			$0.6_{-}$			$0.6_{-}$			$0.6_{-}$			$0.6_{-}$			$0.7_{-}$			$0.5_{-}$			$0.6_{-}$	
	Vigorous					$0.3_{-}$	$0.4_{-}$												$0.6_{-}$					$0.4_{-}$	$0.4_{-}$			$0.7_{-}$		$0.3_{-}$	$0.7_{-}$						$0.5_{-}$			$0.5_{-}$			$0.6_{-}$			$0.6_{-}$			$0.5_{-}$	0.6		$0.6_{-}$	0.5
	Active		0.6	0.6		0.5	0.6		0.4	0.6		0.5	0.6		0.5	0.6		0.6	0.7	$0.5_{-}$	0.6	0.5	$0.7_{-}$	0.5	0.6		0.4	0.6	$0.6_{-}$	0.5	0.6		0.6	0.7		0.3	0.6	$0.7_{-}$	0.6	0.6		0.6	0.7		0.6	0.7	$0.5_{-}$	0.7	0.8		0.6	0.7	
	Sleep				0.5			0.5			0.6			0.6	0.4		0.6															0.5	0.4		0.6				0.3		0.7												0.6
CLR PA	Sedentary								$0.3_{-}$																								$0.3_{-}$				$0.4_{-}$											$0.3_{-}$		$0.5_{-}$	$0.3_{-}$		
	Light		0.3	0.4		0.3	0.6		0.4	0.6		0.3	0.4		0.4	0.4			0.5			0.5	$0.6_{-}$	0.5	0.4		0.5	0.6	$0.6_{-}$	0.4	0.5		0.3	0.4		0.5	0.5		0.3	0.4		0.3	0.4		0.5	0.5		0.6	0.8		0.3	0.5	
	Fair		0.3	$0.5_{-}$		$0.3_{-}$	$0.4_{-}$		0.4	0.5			$0.5_{-}$					0.4			0.5			$0.7_{-}$			0.6	0.5		$0.4_{-}$	$0.4_{-}$		0.6	0.8		0.6	0.6		$0.3_{-}$	$0.4_{-}$		0.7	0.6		0.5						0.6	0.5	
	Vigorous		$0.6_{-}$			0.7	0.4		0.7	0.6			0.4		0.7	0.4		0.4	0.5		0.5	0.4		0.6	0.6	0.6	0.4			0.7	0.4		0.6		0.6	0.3	0.4		$0.6_{-}$			0.3		0.6	$0.6_{-}$	0.5		$0.7_{-}$	0.4		0.5	0.4	
CLR PA+S	Sedentary			0.7	$0.7_{-}$					0.5	$0.5_{-}$		0.4												0.6		0.4	0.5			0.5	$0.6_{-}$	$0.4_{-}$	0.5			$0.4_{-}$			0.7	$0.6_{-}$		0.5							$0.6_{-}$		0.6	$0.6_{-}$
	Light			0.4		0.3	0.5		0.3	0.5		0.3	0.5		0.4	0.4		0.4	0.5		0.5	0.5		0.6	0.5		0.4	0.6		0.5	0.5		0.4	0.4		0.4	0.5			0.4		0.3	0.4		0.4	0.5			0.4		0.4	0.5	
	Fair		0.5	0.5		0.7	0.7		0.8	0.9		0.7	0.4		0.7	0.8		0.3	0.6		$0.5_{-}$	0.6		0.8	0.8		$0.5_{-}$			0.7	0.7		0.6	0.7		0.6	0.6		$0.4_{-}$	0.5		0.8	0.8		0.6	0.6		0.5	0.5	$0.6_{-}$	0.7	0.8	
	Vigorous			0.6		0.8	0.6		0.7	0.6			0.4		0.8	0.6		0.3	$0.6_{-}$		0.3	$0.6_{-}$		0.8	0.6		0.3	$0.5_{-}$	$0.6_{-}$	0.8	0.6	$0.5_{-}$				0.5	$0.5_{-}$		$0.4_{-}$	0.5			0.4		0.3	$0.5_{-}$	0.5	0.4	$0.6_{-}$		0.4	0.4	
	Sleep																0.6	$0.3_{-}$	$0.5_{-}$		$0.5_{-}$	$0.4_{-}$					$0.3_{-}$	$0.5_{-}$					$0.6_{-}$	$0.4_{-}$		$0.3_{-}$	$0.4_{-}$						$0.5_{-}$		$0.3_{-}$	$0.4_{-}$							
Raw	Total							1	1	1						1		1	1	1	1	1	1					1	1				1	1			1	1			1				1	1	1		1		1	1	
Processed	Total		2	5	4	2	6	5	3	6	2	2	4	2	4	6	2	4	7	3	3	6	3	4	5		2	6	2	2	7	3	5	6	1	2	7	2	2	7	4	4	7		3	6	2	4	7	1	4	7	4
CLR PA	Total		1	1		1	1		1	3			1		1				2		2	1	1	3	1	1	2	2	1	1	1		2	1	1	2	2		1			1	1	1	3	2		2	1	1	2	2	
CLR PA+S	Total		1	3	1	2	3		2	4	1	1	1		2	2	1		4		3	3		3	4		1	4	1	3	4	2	2	2		2	3			3	1	1	3		1	3	1	1	2	2	1	3	1
All Families	Total		4	9	5	5	10	6	7	14	3	3	6	2	7	9	3	5	14	4	9	11	5	10	10	1	5	13	5	6	12	5	10	10	2	6	13	3	3	10	6	6	11	1	8	12	4	7	11	4	8	13	5

Color coding: from orange (weaker correlation/fewer total correlations) to yellow to green (stronger correlation/more total correlations). Only significant correlations are shown. Only coefficients of 0.5 or above are highlighted.

**Table A15:** Correlation coefficient (Spearman $r_{S}$) between TechROs from Fitbit (rows) and PROs of Depression and Anxiety on the GADS scale (columns).

	PRO		Q1A: Keyed-up or on edge			Q2A: Worrying a lot?			Q3A: Irritable?			Q4A: Difficulty relaxing?			Q5A: Sleeping poorly?			Q6A: Headaches or neck aches?			Q7A: Trembling [...] urine?			Q8A: Worried about your health?			Q9A: Difficulty falling asleep?			Q1D: Lacking energy?			Q2D: Lost interest in things?			Q3D: Lost confidence in yourself?			Q4D: Hopeless?			Q5D: Difficulty concentrating?			Q6D: Lost weight due to poor appetite?			Q7D: Waking early?			Q8D: Slowed down?			Q9D: Worse in the mornings?			Numeric score			Categorical score		
Family	TechRO	Health	Both	Healthy	Diseased	Both	Healthy	Diseased	Both	Healthy	Diseased	Both	Healthy	Diseased	Both	Healthy	Diseased	Both	Healthy	Diseased	Both	Healthy	Diseased	Both	Healthy	Diseased	Both	Healthy	Diseased	Both	Healthy	Diseased	Both	Healthy	Diseased	Both	Healthy	Diseased	Both	Healthy	Diseased	Both	Healthy	Diseased	Both	Healthy	Diseased	Both	Healthy	Diseased	Both	Healthy	Diseased	Both	Healthy	Diseased	Both	Healthy	Diseased	Both	Healthy	Diseased
Raw	Energy				$0.6_{-}$			$0.5_{-}$			$0.7_{-}$			$0.5_{-}$	$0.2_{-}$		$0.5_{-}$	$0.3_{-}$					$0.6_{-}$				$0.4_{-}$	$0.4_{-}$	$0.7_{-}$			$0.7_{-}$			$0.5_{-}$						$0.5_{-}$			$0.5_{-}$					0.4	$0.5_{-}$			$0.6_{-}$			$0.5_{-}$	$0.3_{-}$		$0.5_{-}$	$0.3_{-}$		$0.5_{-}$
	Steps					0.4	0.6			$0.4_{-}$	$0.6_{-}$						$0.7_{-}$		$0.4_{-}$		$0.2_{-}$								$0.5_{-}$			$0.6_{-}$	$0.4_{-}$	$0.3_{-}$														0.4	0.4		$0.2_{-}$	$0.3_{-}$	$0.5_{-}$									
	Heart rate							0.6					0.5			0.4			0.6		$0.3_{-}$	$0.3_{-}$	$0.6_{-}$			$0.7_{-}$	0.4	0.6					$0.4_{-}$		$0.5_{-}$			$0.5_{-}$															$0.5_{-}$									
Processed	Sedentary								0.4		0.7			0.6																		0.6	0.4	0.4		0.4	0.4								0.4									$0.3_{-}$		$0.5_{-}$				0.4		
	Sedentary+light					0.3		0.6		$0.3_{-}$		0.5						$0.3_{-}$	$0.5_{-}$										0.5		$0.4_{-}$											0.3			0.6					0.5		$0.4_{-}$			$0.5_{-}$							
	Light							0.7	$0.3_{-}$	$0.5_{-}$											$0.3_{-}$	$0.5_{-}$								$0.5_{-}$	$0.5_{-}$		$0.6_{-}$	$0.6_{-}$		$0.5_{-}$	$0.5_{-}$												0.4	0.6	$0.6_{-}$	$0.4_{-}$								$0.5_{-}$		
	Light+fair							0.7	$0.3_{-}$	$0.7_{-}$								$0.3_{-}$	$0.5_{-}$		$0.4_{-}$	$0.8_{-}$		$0.5_{-}$	$0.5_{-}$		$0.3_{-}$	$0.5_{-}$		$0.5_{-}$	$0.5_{-}$		$0.7_{-}$	$0.6_{-}$		$0.5_{-}$				$0.5_{-}$					0.5						$0.6_{-}$	$0.6_{-}$		$0.3_{-}$	$0.4_{-}$			$0.6_{-}$			$0.6_{-}$	
	Fair									$0.3_{-}$					$0.6_{-}$	$0.6_{-}$		$0.4_{-}$	$0.4_{-}$		$0.3_{-}$	$0.5_{-}$		0.6			$0.7_{-}$	$0.7_{-}$					0.3	0.7					$0.4_{-}$			$0.4_{-}$			$0.5_{-}$	$0.5_{-}$		0.4			0.6			$0.4_{-}$	$0.5_{-}$			$0.4_{-}$				
	Fair+vigorous							$0.7_{-}$		$0.4_{-}$			0.6		$0.3_{-}$	$0.4_{-}$			0.6					0.7			$0.6_{-}$	$0.4_{-}$		0.5	0.6			0.5											$0.5_{-}$	$0.6_{-}$					0.6	0.5		$0.3_{-}$	$0.4_{-}$							
	Vigorous							$0.6_{-}$		0.6			0.6		$0.3_{-}$							0.8	$0.7_{-}$	0.7			$0.4_{-}$	$0.3_{-}$						0.7		0.3	0.4				$0.5_{-}$				$0.4_{-}$	$0.4_{-}$						0.6		$0.3_{-}$								
	Active								$0.5_{-}$	$0.8_{-}$		$0.5_{-}$	$0.5_{-}$		$0.3_{-}$	$0.5_{-}$	$0.5_{-}$	$0.3_{-}$	$0.5_{-}$		$0.8_{-}$	$0.8_{-}$			$0.6_{-}$		$0.4_{-}$	$0.6_{-}$	$0.6_{-}$	$0.4_{-}$	$0.6_{-}$		$0.6_{-}$	$0.7_{-}$	$0.5_{-}$		$0.5_{-}$	$0.6_{-}$	$0.7_{-}$	$0.6_{-}$	$0.6_{-}$											$0.7_{-}$		$0.5_{-}$	$0.5_{-}$		$0.5_{-}$	$0.6_{-}$		$0.5_{-}$	$0.6_{-}$	
	Sleep					$0.3_{-}$		$0.5_{-}$			$0.5_{-}$	$0.3_{-}$		$0.5_{-}$				0.3	0.4										$0.6_{-}$		0.4														$0.5_{-}$		$0.5_{-}$				0.4	0.4			0.3							
CLR PA	Sedentary									0.4		0.4	0.5		0.7	0.6		0.3			0.5	0.4		$0.5_{-}$			0.7	0.5											0.5		0.5	0.5			0.4			$0.4_{-}$	$0.5_{-}$					0.4	0.5					0.4		
	Light		0.5	0.6		0.4	0.4	0.7							0.4							$0.8_{-}$		$0.6_{-}$			0.5			$0.6_{-}$			$0.4_{-}$	$0.8_{-}$								0.4			0.6	0.5					$0.5_{-}$	$0.6_{-}$		0.5			0.4					
	Fair							0.6				0.3	0.3								0.5			0.5	0.6		$0.6_{-}$		$0.5_{-}$	0.6	0.6			0.6		$0.6_{-}$	$0.5_{-}$		0.4	$0.3_{-}$		0.3	0.5		0.6	$0.5_{-}$		0.4	0.4		$0.4_{-}$						0.6	0.6				
	Vigorous								0.8	0.3		0.5	0.7		$0.4_{-}$			$0.5_{-}$	$0.5_{-}$					0.5		0.6	$0.5_{-}$			0.7	0.6		0.7	0.6		$0.3_{-}$	$0.4_{-}$								0.6	0.7					0.5						$0.4_{-}$	0.5		$0.4_{-}$	$0.5_{-}$	
CLR PA+S	Sedentary				0.5					0.5			0.5		0.5	0.6					0.4	0.4	0.5	$0.7_{-}$			0.4	0.5	0.6	$0.6_{-}$	$0.7_{-}$			$0.5_{-}$					0.5		0.5						0.5				$0.6_{-}$	$0.6_{-}$		0.6	0.8							
	Light							0.7							0.8												0.6									$0.3_{-}$	$0.3_{-}$					0.6	0.6		0.8	0.4		$0.5_{-}$						0.7			0.6	0.5		0.5		
	Fair											0.4	0.4		0.5						0.5			$0.5_{-}$	0.4	0.6	0.4			0.6	0.4		$0.4_{-}$	0.5		$0.7_{-}$	$0.5_{-}$		0.6	0.4		0.6	0.5		$0.6_{-}$	$0.4_{-}$	0.6	0.5	0.4		$0.4_{-}$	$0.6_{-}$		0.5	0.5		0.5	$0.5_{-}$		0.5		
	Vigorous		$0.5_{-}$						0.6	0.6	0.5	0.4	0.6		0.3	0.5					$0.4_{-}$	0.8		0.4						$0.8_{-}$	0.5		$0.4_{-}$	0.5		0.5	0.5			0.5		0.4			0.7	0.5		$0.4_{-}$			$0.4_{-}$				0.5		0.5	0.5		0.4	0.5	
	Sleep		0.5			0.6	0.6		0.5	0.5	$0.6_{-}$	0.6		$0.5_{-}$	0.7	0.7		0.6	0.7		0.3	0.4			0.6	0.7	0.6	0.6		0.5	0.4		0.5						0.7	0.7		0.7	0.7		0.5	0.8					0.4	0.5		0.5	0.5		0.8	0.5		0.6	0.6	
Raw	Total				1		1	2			2		1	1			2		1				2			1		1	2			2			2			1			1			1						1			3			1			1			1
Processed	Total							6	1	4	2	2	3	2	1	2	1		4		1	5	1	4	2		2	3	3	3	4	1	3	6	1	2	2	1	1	2	2				5	2	1			2	4	4		1	3	1	1	2		2	2	
CLR PA	Total		1	1				2	1			1	2		1	1		1	1		2	1		4	1	1	4	1	1	3	2		1	3		1	1		1		1	1	1		3	3			1		2	1		1	1		1	2			1	
CLR PA+S	Total		2		1	1	1	1	2	3	2	1	2	1	4	3		1	1		1	1	1	2	1	2	2	2	1	4	2		1	3		2	2		3	2	1	3	3		4	2	2	2			1	3		4	4		4	4		3	2	
All Families	Total		3	1	2	1	2	11	4	7	6	4	8	4	6	6	3	2	7		4	7	4	10	4	4	8	7	7	10	8	3	5	12	3	5	5	2	5	4	5	4	4	1	12	7	3	2	1	3	7	8	3	6	8	2	6	8	1	5	5	1

Color coding: from orange (weaker correlation/fewer total correlations) to yellow to green (stronger correlation/more total correlations). Only significant correlations are shown. Only coefficients of 0.5 or above are highlighted.

**Table A16:** Correlation coefficient (Spearman $r_{S}$) between TechROs from Fitbit (rows) and PROs of Mediterranean Nutrition on the PREDIMED scale (columns).

	PRO		Q2: Olive oil use			Q3: Vegetables use			Q4: Fruits use			Q5: Red meat, hamburger, or meat use			Q6: Butter, margarine, or cream use			Q7: Sweet/carbonated beverage use			Q8: Wine use			Q9: Legumes use			Q10: Fish or shellfish use			Q11: Commercial sweets or pastries			Q12: Nuts use			Q14: Sofrito use			Numeric score			Categorical score		
Family	TechRO	Health	Both	Healthy	Diseased	Both	Healthy	Diseased	Both	Healthy	Diseased	Both	Healthy	Diseased	Both	Healthy	Diseased	Both	Healthy	Diseased	Both	Healthy	Diseased	Both	Healthy	Diseased	Both	Healthy	Diseased	Both	Healthy	Diseased	Both	Healthy	Diseased	Both	Healthy	Diseased	Both	Healthy	Diseased	Both	Healthy	Diseased
Raw	Energy								$0.4_{-}$	$0.4_{-}$				0.8							0.4	0.4																						$0.6_{-}$
	Steps		0.3			0.4	0.4		$0.4_{-}$	$0.5_{-}$					$0.3_{-}$	$0.4_{-}$					0.5	0.4					0.3						0.5	0.6					0.4	0.4		0.5	0.4	
	Heart rate		0.4	0.5					0.4						$0.6_{-}$	$0.4_{-}$					$0.4_{-}$			$0.4_{-}$		$0.6_{-}$	0.3			0.3		0.8	0.6	0.4					0.5	0.4		0.5	0.4	
Processed	Sedentary					$0.5_{-}$			0.5	0.4								0.5	0.6		$0.3_{-}$	$0.6_{-}$		$0.4_{-}$																$0.6_{-}$			$0.6_{-}$	
	Sedentary+light		0.3	0.5		$0.4_{-}$			0.5	0.6			0.5		$0.4_{-}$										0.4		0.4												0.3					
	Light						0.5											$0.4_{-}$			0.5	0.6					0.3						0.5	0.4					0.6	0.6		0.5	0.5	
	Light+fair					$0.4_{-}$												0.4	0.5		0.4	0.6					0.3						0.3											
	Fair		0.4							0.7		$0.4_{-}$	$0.6_{-}$								0.4												$0.9_{-}$			$0.5_{-}$	$0.6_{-}$			0.4		$0.6_{-}$		
	Fair+vigorous									0.6																										$0.6_{-}$			$0.7_{-}$			$0.7_{-}$		
	Vigorous														0.6																								$0.8_{-}$			$0.7_{-}$		
	Active					$0.4_{-}$	$0.5_{-}$											0.5	0.5		0.6			0.6						0.4														
	Sleep					0.4			$0.4_{-}$	$0.4_{-}$		$0.5_{-}$	$0.5_{-}$		0.4																								$0.3_{-}$			$0.3_{-}$		
CLR PA	Sedentary									$0.6_{-}$																							0.5	0.7			0.5		0.6			0.7	0.6	
	Light		0.7									0.6	0.5																	$0.6_{-}$			0.4						0.8			0.7		
	Fair					$0.6_{-}$	$0.8_{-}$		$0.4_{-}$	$0.6_{-}$		0.4			$0.6_{-}$			0.4							$0.5_{-}$					0.5									0.6					
	Vigorous								$0.5_{-}$						0.4	0.5		0.4	0.5		$0.4_{-}$						$0.6_{-}$	$0.7_{-}$		0.5									$0.5_{-}$	$0.7_{-}$		$0.5_{-}$	$0.6_{-}$	
CLR PA+S	Sedentary																										0.6			$0.6_{-}$			0.6	0.6		0.6	0.6		0.6			0.6	0.6	
	Light			0.6					0.6	$0.5_{-}$		0.6	0.5								$0.6_{-}$			$0.4_{-}$			$0.6_{-}$			$0.6_{-}$			0.8			0.6				$0.6_{-}$				
	Fair					$0.4_{-}$	$0.8_{-}$		$0.5_{-}$			0.6			$0.6_{-}$									$0.5_{-}$	$0.5_{-}$		0.6									0.7	0.7							
	Vigorous		$0.6_{-}$	0.6		$0.5_{-}$	$0.4_{-}$					0.4				0.6		0.4												$0.7_{-}$						0.7	0.6			$0.4_{-}$			$0.5_{-}$	
	Sleep																				$0.7_{-}$	$0.7_{-}$		$0.6_{-}$			$0.6_{-}$	$0.6_{-}$								0.6								
Raw	Total			1						1				1	1						1					1						1	2	1					1			2		1
Processed	Total			1		1	2		2	3		1	3		1			2	3		2	3		1									2			2	1		3	2		4	2	
CLR PA	Total		1			1	1		1	2		1	1		1	1			1						1		1	1		3			1	1			1		4	1		3	2	
CLR PA+S	Total		1	2		1	1		2	1		2	1		1	1					2	1		2	1		4	1		3			2	1		5	3		1	1		1	2	
All Families	Total		2	4		3	4		5	7		4	5	1	4	2		2	4		5	4		3	2	1	5	2		6		1	7	3		7	5		9	4		10	6	1

Color coding: from orange (weaker correlation/fewer total correlations) to yellow to green (stronger correlation/more total correlations). Only significant correlations are shown. Only coefficients of 0.5 or above are highlighted.

**Table A17:** Correlation coefficient (Spearman $r_{S}$) between TechROs from Fitbit (rows) and PROs of Nutrition on the SelfMNA scale (columns).

	PRO		Q1: Food Intake Declined			Q2: Weight Lost			Q4: Stressed or Severely Ill			Q5: Dementia and/or Severe Sadness			Numeric Score			Categorical Score		
Family	TechRO	Health	Both	Healthy	Diseased	Both	Healthy	Diseased	Both	Healthy	Diseased	Both	Healthy	Diseased	Both	Healthy	Diseased	Both	Healthy	Diseased
Raw	Energy				$0.5_{-}$			$0.5_{-}$			0.4									
	Steps																			
	Heart rate																			
Processed	Sedentary				0.5			0.6			$0.8_{-}$				0.4					
	Sedentary+light				0.5			0.6												
	Light																		$0.5_{-}$	
	Light+fair																	$0.5_{-}$		
	Fair																	0.6		
	Fair+vigorous																	0.4		
	Vigorous														0.4			0.4		
	Active							$0.5_{-}$			0.6									
	Sleep										0.5				0.4			0.4		
CLR PA	Sedentary																	$0.6_{-}$	$0.6_{-}$	
	Light																	$0.6_{-}$		
	Fair				$0.5_{-}$			$0.5_{-}$								0.6				
	Vigorous															0.7		0.4	0.5	
CLR PA+S	Sedentary																	$0.6_{-}$		
	Light																		$0.5_{-}$	
	Fair															$0.7_{-}$				
	Vigorous				$0.7_{-}$			$0.7_{-}$												
	Sleep				$0.8_{-}$			$0.8_{-}$			0.6							0.6	0.5	
Raw	Total				1			1												
Processed	Total				2			3			3							2	1	
CLR PA	Total				1			1								2		2	2	
CLR PA+S	Total				2			2			1					1		2	2	
All Families	Total				6			7			4					3		6	5	

Color coding: from orange (weaker correlation/fewer total correlations) to yellow to green (stronger correlation/more total correlations). Only significant correlations are shown. Only coefficients of 0.5 or above are highlighted.

**Table A18:** Correlation coefficient (Spearman $r_{S}$) between TechROs from Fitbit (rows) and PROs of Memory on the MFE scale (columns).

	PRO		Q1: Forgetting objects put			Q2: Failing to recognise places			Q3: Finding a television story difficult			Q4: Not remembering a change in daily routine			Q5: Checking whether something was done			Q6: Forgetting the time of events			Q7: Completely forgetting to take things			Q8: Being reminded about things			Q9: Reading anew something already read			Q10: Letting ramble about unimportant things			Q11: Failing to recognise close relatives or friends			Q12: Having difficulty picking up a new skill			Q13: Finding word is “on the tip of the tongue”			Q14: Forgetting to do planned things			Q15: Forgetting important details of done things			Q16: Forgetting the topic of an ongoing conversation			Q17: Failing to follow a story in a newspaper			Q18: Forgetting to tell somebody something important			Q20: Getting told details mixed up and confused			Q21: Telling someone a story or joke repeatedly			Q22: Forgetting details of things you do regularly			Q23: Finding famous faces unfamiliar			Q24: Forgetting where things are normally kept			Q25: Getting lost where you have OFTEN been before			Q26: Getting lost where you have been RARELY before			Q27: Doing some routine thing twice by mistake			Q28: Repeating to someone what you have just told them			Numeric score			Categorical score		
Family	TechRO	Health	Both	Healthy	Diseased	Both	Healthy	Diseased	Both	Healthy	Diseased	Both	Healthy	Diseased	Both	Healthy	Diseased	Both	Healthy	Diseased	Both	Healthy	Diseased	Both	Healthy	Diseased	Both	Healthy	Diseased	Both	Healthy	Diseased	Both	Healthy	Diseased	Both	Healthy	Diseased	Both	Healthy	Diseased	Both	Healthy	Diseased	Both	Healthy	Diseased	Both	Healthy	Diseased	Both	Healthy	Diseased	Both	Healthy	Diseased	Both	Healthy	Diseased	Both	Healthy	Diseased	Both	Healthy	Diseased	Both	Healthy	Diseased	Both	Healthy	Diseased	Both	Healthy	Diseased	Both	Healthy	Diseased	Both	Healthy	Diseased	Both	Healthy	Diseased	Both	Healthy	Diseased	Both	Healthy	Diseased
Raw	Energy										$0.5_{-}$			$0.5_{-}$																						$0.4_{-}$		$0.6_{-}$	$0.4_{-}$	$0.4_{-}$	$0.7_{-}$																$0.3_{-}$	$0.6_{-}$														0.4												$0.2_{-}$		$0.5_{-}$	$0.2_{-}$		$0.5_{-}$
	Steps		$0.3_{-}$						$0.2_{-}$			$0.4_{-}$			$0.6_{-}$	$0.6_{-}$		$0.4_{-}$	$0.5_{-}$		$0.4_{-}$	$0.6_{-}$	0.5		$0.3_{-}$	0.5																$0.3_{-}$	$0.5_{-}$								$0.3_{-}$	$0.3_{-}$								$0.4_{-}$										$0.3_{-}$	0.6	0.3				$0.5_{-}$								$0.5_{-}$	$0.6_{-}$		$0.4_{-}$	$0.5_{-}$	
	Heart rate												$0.4_{-}$	0.5	0.3	0.4				0.8																0.3			0.4	0.4		$0.4_{-}$												0.3	0.4			0.5				$0.5_{-}$	$0.4_{-}$		$0.5_{-}$							$0.3_{-}$						0.5	0.5		0.3	0.4							
Processed	Sedentary											0.4				0.4		0.6	0.5					0.4					0.7							0.6	0.6				0.7										0.3	0.3																				$0.4_{-}$		$0.6_{-}$										0.6	0.5		0.4	0.4	
	Sedentary+light			$0.5_{-}$	0.6	0.4	0.3		0.5		0.7			0.6	0.2	0.3				0.7		$0.4_{-}$	0.7	$0.3_{-}$	$0.4_{-}$					$0.3_{-}$	$0.5_{-}$					0.6	0.5	0.6	0.3	$0.4_{-}$	0.6		$0.4_{-}$			$0.7_{-}$					0.6	0.6			$0.6_{-}$		$0.4_{-}$				$0.6_{-}$									$0.3_{-}$		$0.4_{-}$		$0.6_{-}$											$0.3_{-}$	0.7		$0.5_{-}$	
	Light				0.6							$0.4_{-}$	$0.6_{-}$		$0.3_{-}$	$0.6_{-}$	0.6	$0.5_{-}$	$0.6_{-}$	0.8	$0.3_{-}$	$0.5_{-}$	0.7		$0.3_{-}$	0.6										$0.5_{-}$	$0.6_{-}$					$0.6_{-}$	$0.5_{-}$	$0.5_{-}$	$0.4_{-}$	$0.5_{-}$						$0.3_{-}$				0.6	$0.3_{-}$	$0.5_{-}$																										$0.6_{-}$	$0.7_{-}$	0.6	$0.5_{-}$	$0.5_{-}$	0.5
	Light+fair					0.4	0.4					$0.5_{-}$	$0.6_{-}$					$0.6_{-}$	$0.5_{-}$			$0.4_{-}$			$0.6_{-}$	0.8				$0.6_{-}$	$0.8_{-}$						$0.5_{-}$		0.3		0.5	$0.7_{-}$	$0.5_{-}$		$0.7_{-}$	$0.6_{-}$	0.7	$0.3_{-}$							$0.5_{-}$		$0.5_{-}$							$0.5_{-}$		$0.4_{-}$	$0.5_{-}$							$0.5_{-}$										$0.4_{-}$	$0.7_{-}$	0.6		$0.6_{-}$	0.6
	Fair					0.4	0.4								0.5	0.4			0.6	$0.6_{-}$				$0.3_{-}$	$0.4_{-}$		0.4									$0.5_{-}$	$0.6_{-}$		$0.5_{-}$							0.7		$0.6_{-}$	$0.8_{-}$			$0.4_{-}$			0.5	$0.8_{-}$							$0.6_{-}$	$0.6_{-}$				$0.6_{-}$		0.7		0.6			0.5									$0.3_{-}$					$0.5_{-}$
	Fair+vigorous			0.6	$0.5_{-}$	0.3	0.4			$0.5_{-}$					0.6	0.7			0.7	$0.5_{-}$	$0.3_{-}$	0.5	$0.6_{-}$																$0.5_{-}$		$0.6_{-}$	0.5	0.8						$0.4_{-}$					$0.3_{-}$		$0.8_{-}$					0.7		$0.3_{-}$					$0.6_{-}$		0.6		0.4		0.6	0.6		0.6								0.6	$0.6_{-}$			$0.5_{-}$
	Vigorous		$0.4_{-}$		$0.5_{-}$	0.3				$0.4_{-}$			0.7		0.6		$0.5_{-}$		0.6		$0.3_{-}$	0.7	$0.6_{-}$			$0.5_{-}$					0.7								$0.6_{-}$		$0.7_{-}$	0.5	0.8			0.6								$0.3_{-}$		$0.8_{-}$					0.7		$0.3_{-}$		$0.6_{-}$							0.4		0.7	0.7										0.7	$0.6_{-}$			$0.6_{-}$
	Active				$0.7_{-}$	0.5	0.4					$0.5_{-}$	$0.5_{-}$					$0.7_{-}$	$0.6_{-}$		$0.3_{-}$	$0.4_{-}$		$0.6_{-}$						$0.7_{-}$	$0.7_{-}$					$0.6_{-}$	$0.7_{-}$		$0.5_{-}$		$0.8_{-}$	$0.4_{-}$	$0.6_{-}$		$0.7_{-}$	$0.7_{-}$								$0.5_{-}$	$0.5_{-}$					$0.7_{-}$			$0.5_{-}$	$0.6_{-}$		$0.5_{-}$	$0.5_{-}$		$0.4_{-}$			0.5		0.6	0.4			$0.4_{-}$	$0.6_{-}$					$0.8_{-}$	$0.7_{-}$		$0.6_{-}$	$0.7_{-}$	
	Sleep			0.6	$0.6_{-}$	$0.4_{-}$	$0.4_{-}$		$0.4_{-}$						$0.3_{-}$	$0.3_{-}$		$0.3_{-}$		$0.8_{-}$		0.4	$0.5_{-}$		0.4	$0.6_{-}$	$0.5_{-}$		$0.8_{-}$	0.3	0.3					$0.5_{-}$					$0.6_{-}$	0.5	0.5	0.5	0.5	0.5	$0.6_{-}$				$0.5_{-}$	$0.6_{-}$	$0.6_{-}$	0.6	0.5		0.4	0.4			0.5								0.5	0.7		0.3		0.7												$0.6_{-}$	0.5	0.6	
CLR PA	Sedentary			$0.5_{-}$	0.6	$0.3_{-}$	$0.5_{-}$							0.6			0.5			0.6	0.3		0.6	0.4		0.6				0.5						0.6	0.7		0.6		0.7				0.3	0.4			0.4			0.3		0.3		0.8				0.4			0.7	0.7	0.6			0.5		$0.6_{-}$		$0.4_{-}$		$0.7_{-}$	$0.6_{-}$		$0.6_{-}$	0.5	0.6							0.6			
	Light											$0.6_{-}$	$0.5_{-}$		$0.6_{-}$		0.6	$0.7_{-}$	$0.7_{-}$				0.5			0.6					$0.7_{-}$								0.3		0.6	$0.7_{-}$	$0.6_{-}$		$0.6_{-}$	$0.5_{-}$				$0.6_{-}$						0.8	$0.4_{-}$	$0.5_{-}$		$0.6_{-}$	$0.7_{-}$											$0.5_{-}$		$0.6_{-}$	$0.5_{-}$										$0.8_{-}$	0.6	$0.5_{-}$	$0.5_{-}$	0.6
	Fair		0.6		$0.7_{-}$				$0.3_{-}$		$0.6_{-}$	0.3				0.4		0.7	0.6		0.6			0.3	$0.7_{-}$					$0.4_{-}$						0.4	0.5		0.4		0.6	0.6	0.7		0.7			$0.7_{-}$	$0.5_{-}$											0.7			$0.5_{-}$	$0.5_{-}$		0.5	0.4	$0.6_{-}$	0.8	0.5		$0.3_{-}$		$0.6_{-}$				0.3	0.4		0.6					0.7			
	Vigorous				$0.8_{-}$				0.3						0.5			0.7	0.7			0.5		$0.5_{-}$	$0.7_{-}$					0.3	0.5					0.3	0.5	0.5							0.7	0.7											0.4	0.6			0.7	0.6	$0.3_{-}$		$0.5_{-}$												0.6	0.6	0.6					0.5	0.7		0.6	0.6	
CLR PA+S	Sedentary				0.6	$0.3_{-}$	$0.4_{-}$										0.6			0.7	0.4		0.7			0.7				0.5						0.6	0.7		0.5		0.8		$0.6_{-}$					0.5	0.4			0.4				0.8	$0.5_{-}$					0.5	0.7	0.7	0.6					$0.6_{-}$		$0.4_{-}$		$0.7_{-}$	$0.6_{-}$		$0.6_{-}$	0.4	0.4	0.6	0.5			0.3		0.7			
	Light								0.3				$0.4_{-}$				0.6		$0.6_{-}$		0.4	0.6					$0.5_{-}$			$0.5_{-}$	$0.6_{-}$					$0.8_{-}$					0.7				$0.4_{-}$	$0.5_{-}$										0.7	$0.4_{-}$				$0.5_{-}$					$0.6_{-}$	$0.7_{-}$					$0.4_{-}$		$0.7_{-}$			$0.6_{-}$				0.4							$0.3_{-}$	0.6
	Fair			$0.6_{-}$	$0.7_{-}$				$0.4_{-}$				0.5					$0.7_{-}$	$0.7_{-}$			$0.5_{-}$		0.6			0.3			0.4	0.5											$0.7_{-}$	$0.6_{-}$		$0.7_{-}$	$0.7_{-}$			0.5									$0.5_{-}$		$0.6_{-}$	$0.7_{-}$	0.6	0.5			0.5	0.6		$0.8_{-}$	$0.8_{-}$									0.3								$0.6_{-}$	$0.6_{-}$	
	Vigorous								0.3	0.4					0.5	0.5		$0.5_{-}$	$0.5_{-}$								$0.3_{-}$			0.6	0.7					0.6	0.5					$0.6_{-}$	$0.4_{-}$		$0.4_{-}$	$0.5_{-}$								$0.7_{-}$								0.6	$0.3_{-}$						$0.3_{-}$									0.6	0.5						0.5			0.5	
	Sleep											0.5				0.5					0.8	0.8														0.6	0.7		0.8			0.5	0.6		0.3	0.5				$0.5_{-}$				0.5						0.6						0.6	0.5								$0.4_{-}$			0.6	0.5		0.5	0.4		0.5	0.6		0.4	0.4	
Raw	Total										1			2	1	1			1	1		1	1			1												1			1		1															2				1			1						1					1		1	1					1	1	1		1	1
Processed	Total			3	6	1			1	1	1	2	4	1	3	2	2	4	7	5		3	5	1	1	4	1		2	2	4					6	6	1	4		7	5	6	2	3	7	2	1	1		2	2	1	2	5	4	1	1		1	4		2	3	1	1	2	2	1	3		2		7	3		1		1					3	6	6	3	5	5
CLR PA	Total		1	1	3		1				1	1	1	1	2		2	3	3	1	1	1	2	1	2	2				1	2					1	3	1	1		3	2	2		3	2		1	1	1						2		2		2	2	1	2	2	2	1		2	1	2		1		3	2		2	2	2		1			1	2	3	2	2	1
CLR PA+S	Total			1	2							1	1		1	2	2	2	3	1	1	3	1	1		1	1			3	3					4	3		2		2	3	3		1	4		1	1	1				2		2	1	1		2	2	3	2	1	1	3	3		1	2				2	1		2	2	2	1	2			1	2	1	1	2	1
All Families	Total		1	5	11	1	1		1	1	3	4	6	4	7	5	6	9	14	8	2	8	9	3	3	8	2		2	6	9					11	12	3	7		13	10	12	2	7	13	2	3	3	2	2	2	1	4	5	8	2	6		5	8	5	6	6	5	5	5	4	3	7	1	3		12	6	1	5	5	6	1	3			6	11	11	6	10	8

Color coding: from orange (weaker correlation/fewer total correlations) to yellow to green (stronger correlation/more total correlations). Only significant correlations are shown. Only coefficients of 0.5 or above are highlighted.

**Table A19:** Correlation coefficient (Spearman $r_{S}$) between TechROs from Fitbit (rows) and PROs of Sleep on the PSQI scale (columns).

	PRO		Q1: time gone to bed at night			Q2: duration taken to fall asleep			Q3: time gotten up in the morning			Q4: duration of actual sleep			Q5A: trouble sleeping: cannot get to sleep			Q5B: trouble sleeping: wake up in the middle of the night			Q5C: trouble sleeping: use the bathroom			Q5D: trouble sleeping: cannot breathe comfortably			Q5E: trouble sleeping: cough or snore loudly			Q5F: trouble sleeping: too cold			Q5G: trouble sleeping: too hot			Q5H: trouble sleeping: bad dreams			Q5I: trouble sleeping: pain			Q5J: trouble sleeping for other reason(s)			Q6: Frequency of medicine to help you sleep			Q7: Trouble staying awake while driving, eating, or socializing			Q8: Problem with keeping up enthusiasm to get things done			Q9: sleep quality overall			Q11: Duration stayed in bed			Numeric score			Categorical score			Quality numeric sub-score			Latency numeric sub-score			Duration numeric sub-score			Efficiency numeric sub-score			Disturbance numeric sub-score			Medication numeric sub-score			Daily dysfunction numeric sub-score			Efficiency numeric sub-score		
Family	TechRO	Health	Both	Healthy	Diseased	Both	Healthy	Diseased	Both	Healthy	Diseased	Both	Healthy	Diseased	Both	Healthy	Diseased	Both	Healthy	Diseased	Both	Healthy	Diseased	Both	Healthy	Diseased	Both	Healthy	Diseased	Both	Healthy	Diseased	Both	Healthy	Diseased	Both	Healthy	Diseased	Both	Healthy	Diseased	Both	Healthy	Diseased	Both	Healthy	Diseased	Both	Healthy	Diseased	Both	Healthy	Diseased	Both	Healthy	Diseased	Both	Healthy	Diseased	Both	Healthy	Diseased	Both	Healthy	Diseased	Both	Healthy	Diseased	Both	Healthy	Diseased	Both	Healthy	Diseased	Both	Healthy	Diseased	Both	Healthy	Diseased	Both	Healthy	Diseased	Both	Healthy	Diseased	Both	Healthy	Diseased
Raw	Energy		$0.4_{-}$	$0.5_{-}$		$0.3_{-}$	$0.4_{-}$	$0.6_{-}$	$0.5_{-}$	$0.8_{-}$			$0.4_{-}$		$0.4_{-}$	$0.5_{-}$				$0.8_{-}$	$0.4_{-}$		$0.8_{-}$				$0.4_{-}$		$0.6_{-}$							$0.4_{-}$	$0.4_{-}$											0.6	0.7		0.3	0.4				$0.6_{-}$			$0.6_{-}$			$0.6_{-}$			0.6			$0.6_{-}$	$0.3_{-}$	$0.4_{-}$								$0.3_{-}$		$0.7_{-}$				0.4	0.6				
	Steps		0.3	0.5		$0.3_{-}$	$0.3_{-}$	$0.7_{-}$									$0.7_{-}$			$0.6_{-}$	$0.3_{-}$		$0.7_{-}$		$0.4_{-}$						0.4	$0.6_{-}$				$0.3_{-}$	$0.3_{-}$																$0.5_{-}$		$0.3_{-}$							$0.6_{-}$					$0.3_{-}$				$0.6_{-}$					$0.3_{-}$		$0.3_{-}$		$0.6_{-}$						$0.6_{-}$			
	Heart rate		0.4	0.4		0.3	0.6		0.6	0.6	0.7	0.5	0.5			0.4									0.4			0.6	$0.6_{-}$					0.3	$0.7_{-}$		0.4	$0.5_{-}$			$0.6_{-}$	$0.4_{-}$	$0.4_{-}$		0.4		0.5	$0.5_{-}$	$0.6_{-}$		$0.5_{-}$			$0.4_{-}$		$0.5_{-}$	0.3	0.3	0.6	$0.4_{-}$						$0.4_{-}$		$0.5_{-}$		0.5		$0.5_{-}$	$0.5_{-}$		$0.3_{-}$		$0.5_{-}$		0.5		0.4		0.5	$0.6_{-}$	$0.5_{-}$	$0.5_{-}$	0.3	0.4	
Processed	Sedentary				0.7	0.3			0.2		0.6			0.5							0.5	0.5					0.4									0.3	0.4		$0.4_{-}$	$0.4_{-}$								$0.4_{-}$				$0.3_{-}$			0.4												0.4							$0.5_{-}$				0.4											
	Sedentary+light		0.4				$0.3_{-}$		0.3			0.3	0.3			$0.4_{-}$							0.5	$0.3_{-}$							$0.3_{-}$							$0.6_{-}$	$0.5_{-}$	$0.5_{-}$								$0.5_{-}$			$0.3_{-}$	$0.3_{-}$						0.3			$0.5_{-}$			0.4						$0.4_{-}$		$0.3_{-}$												$0.4_{-}$		$0.6_{-}$	0.3	0.4	
	Light		0.3	0.4								$0.3_{-}$										$0.5_{-}$		$0.4_{-}$	$0.4_{-}$											$0.4_{-}$	$0.5_{-}$	$0.6_{-}$				$0.4_{-}$	$0.4_{-}$							$0.5_{-}$					$0.5_{-}$		$0.3_{-}$	$0.4_{-}$					0.4	0.5			$0.5_{-}$								$0.3_{-}$	$0.3_{-}$										$0.7_{-}$			
	Light+fair											0.7	0.7		$0.4_{-}$		$0.6_{-}$	$0.6_{-}$	$0.7_{-}$		$0.3_{-}$	$0.8_{-}$		$0.3_{-}$												$0.5_{-}$	$0.4_{-}$				$0.6_{-}$	$0.5_{-}$	$0.5_{-}$					$0.6_{-}$	$0.6_{-}$					$0.5_{-}$	$0.7_{-}$					$0.6_{-}$	$0.6_{-}$	$0.6_{-}$	0.6	0.6		$0.5_{-}$	$0.7_{-}$					$0.7_{-}$	$0.7_{-}$					$0.3_{-}$	$0.4_{-}$					$0.5_{-}$		$0.6_{-}$	0.5		
	Fair		$0.5_{-}$	0.3		$0.6_{-}$	$0.4_{-}$					0.5	$0.6_{-}$	0.8	$0.5_{-}$	$0.5_{-}$					$0.6_{-}$			0.5			$0.4_{-}$												0.7	0.7		$0.3_{-}$	$0.4_{-}$					0.5	0.5		0.6	0.4		$0.5_{-}$	$0.6_{-}$		0.4	0.6								$0.5_{-}$	$0.6_{-}$		$0.5_{-}$	$0.5_{-}$														0.5	0.5				
	Fair+vigorous		$0.6_{-}$				$0.5_{-}$					$0.6_{-}$	$0.5_{-}$	0.8							$0.5_{-}$			0.5			$0.6_{-}$	$0.4_{-}$						$0.7_{-}$														0.5	0.5	0.6	0.3	0.5								0.6	0.8			$0.7_{-}$								0.6	0.7		0.6	0.7								0.5	0.6		$0.7_{-}$	$0.7_{-}$	
	Vigorous		$0.6_{-}$									$0.6_{-}$	$0.6_{-}$	0.9										0.5			$0.4_{-}$	$0.4_{-}$						$0.6_{-}$									0.7					0.7	0.5	0.6	0.4	0.7			0.5	$0.6_{-}$				0.4	0.6			$0.5_{-}$			0.5	$0.6_{-}$				0.6	0.7			0.5								0.8	0.6	0.6	$0.5_{-}$	$0.6_{-}$	
	Active					$0.4_{-}$		$0.7_{-}$				0.6	0.6		$0.3_{-}$		$0.7_{-}$	$0.4_{-}$	$0.7_{-}$		$0.7_{-}$	$0.7_{-}$														$0.4_{-}$	$0.4_{-}$		0.6									0.3	$0.6_{-}$			0.4		$0.5_{-}$	$0.7_{-}$	$0.6_{-}$					$0.6_{-}$	$0.6_{-}$		0.6		$0.5_{-}$	$0.7_{-}$	$0.6_{-}$			$0.7_{-}$							$0.3_{-}$	$0.4_{-}$										
	Sleep		$0.5_{-}$	$0.4_{-}$	$0.8_{-}$																	0.4	$0.6_{-}$	0.3												0.5			0.4											0.6							0.3	0.5		0.4	0.5																												
CLR PA	Sedentary					0.4				$0.6_{-}$			0.5	$0.7_{-}$				0.4	0.4		0.7	0.7					0.6	0.5						0.6			0.4		$0.6_{-}$	$0.5_{-}$			$0.6_{-}$					$0.5_{-}$	$0.6_{-}$	$0.6_{-}$	$0.5_{-}$	$0.6_{-}$		0.3	0.6	0.6										0.3	0.6	0.6		$0.6_{-}$		$0.3_{-}$	$0.5_{-}$			$0.6_{-}$		0.6	0.5					$0.6_{-}$	$0.7_{-}$			0.7	
	Light		0.5				0.5		0.7			0.7	0.4	$0.7_{-}$	0.5	0.5	$0.6_{-}$		$0.6_{-}$			$0.8_{-}$		$0.3_{-}$			0.4									$0.5_{-}$		$0.6_{-}$										$0.8_{-}$	$0.6_{-}$								$0.5_{-}$	$0.6_{-}$											0.6	0.6		$0.7_{-}$			$0.6_{-}$									$0.8_{-}$	$0.5_{-}$	$0.7_{-}$	0.6		
	Fair					$0.6_{-}$	0.4	$0.6_{-}$				$0.7_{-}$	$0.6_{-}$									$0.5_{-}$					$0.5_{-}$	$0.6_{-}$									$0.4_{-}$									0.5		0.6			$0.4_{-}$						0.6	0.5		0.6	0.6		$0.6_{-}$								$0.6_{-}$	0.7	0.6		0.8	0.8						0.5		0.6	0.7		$0.7_{-}$	$0.7_{-}$	
	Vigorous					0.3	0.7					$0.6_{-}$	$0.6_{-}$					$0.6_{-}$	$0.6_{-}$			$0.6_{-}$					$0.8_{-}$	0.7			0.4					$0.7_{-}$	$0.7_{-}$											0.6	0.5					0.5	0.4		$0.7_{-}$	0.6		0.6			$0.6_{-}$			0.5	0.4					0.5						$0.6_{-}$	$0.6_{-}$								$0.5_{-}$		
CLR PA+S	Sedentary		0.4	0.5		0.5						$0.5_{-}$	0.5		0.5						0.7	0.6								0.6									$0.7_{-}$	$0.6_{-}$			$0.5_{-}$					$0.3_{-}$			$0.6_{-}$	$0.5_{-}$		0.4	0.6			$0.6_{-}$								0.4	0.6		0.5				$0.5_{-}$											$0.6_{-}$					
	Light					0.6	0.6															$0.4_{-}$		$0.3_{-}$			0.7	0.8		0.8			0.6			$0.3_{-}$			$0.6_{-}$			$0.3_{-}$	$0.5_{-}$					$0.5_{-}$	$0.4_{-}$		0.4	0.5												$0.5_{-}$											$0.3_{-}$									$0.3_{-}$					
	Fair					0.5			$0.4_{-}$	$0.6_{-}$		0.4	0.6			0.5														0.6			$0.6_{-}$	$0.6_{-}$		$0.5_{-}$												$0.6_{-}$			$0.4_{-}$	0.4		0.4	$0.5_{-}$		0.3	0.5		$0.4_{-}$	$0.5_{-}$			0.4		0.4	$0.5_{-}$			0.5		0.3	0.5		$0.6_{-}$	0.5		$0.3_{-}$						$0.4_{-}$	0.4		0.6	$0.6_{-}$	
	Vigorous					0.6			0.4				$0.4_{-}$		0.4	0.4		0.6	0.8		0.5	0.7					0.7	0.7								0.6			$0.4_{-}$									$0.4_{-}$	$0.4_{-}$		$0.4_{-}$	0.5		$0.5_{-}$	0.4		$0.6_{-}$			$0.6_{-}$	0.4					$0.5_{-}$	0.4			0.5		0.3	0.4					$0.4_{-}$										$0.4_{-}$	
	Sleep		0.6	$0.6_{-}$		0.7	0.8		0.6	$0.5_{-}$		0.5	0.4		0.8	0.8		0.7			0.3						0.7	0.5		0.7			$0.6_{-}$	0.6		$0.6_{-}$	0.7			0.4								$0.8_{-}$	$0.4_{-}$		$0.6_{-}$	$0.6_{-}$		0.3	0.4			0.8					$0.3_{-}$			0.3	0.4		0.8	0.8		$0.5_{-}$												$0.8_{-}$	$0.7_{-}$		0.5		
Raw	Total			2			1	2	2	2	1	1	1			1	1			2			2					1	2			1			1			1			1						1	2	2		1		1			2			2			2			1			2		1	1	1	1				1		1	2			1	1	2	2			
Processed	Total		4		2	1	1	1			1	5	5	4	1	1	2	1	2		4	4	2	3			1							2		2	1	2	3	2	1	1	2					5	5	4	1	2		3	5	2		2		2	6	2	1	5		3	5	2	1	1	1	3	3	1	1	2								4	3	4	3	2	
CLR PA	Total		1			1	2	1	1	1		3	3	2	1	1	1	1	2		1	4					3	3						1		2	1	1	1	1			1			1		4	3	1	1	1		1	1	1	3	3		2	1		2			1	1	1	1	2	1	3	2		2	2		2	2			1		3	3	1	3	2	
CLR PA+S	Total		1	2		5	2		1	2		2	2		2	2		2	1		2	2					3	3		4			3	2		3	1		2	1			2					3			2	4		1	2		1	3		1	1			1		1	2		2	3		1	2		1	1								2	1		2	1	
All Families	Total		6	4	2	7	6	4	4	5	2	11	11	6	4	5	4	4	5	2	7	10	4	3			7	7	2	4		1	3	5	1	7	3	4	6	4	2	1	5			1	1	14	10	5	5	7	1	5	8	5	4	8	2	5	8	4	3	6	1	5	8	5	4	7	3	8	8	1	4	5	1	2	3	2		1	1	10	9	7	8	5	

Color coding: from orange (weaker correlation/fewer total correlations) to yellow to green (stronger correlation/more total correlations). Only significant correlations are shown. Only coefficients of 0.5 or above are highlighted.

**Table A20:** Correlation coefficient (Spearman $r_{S}$) between TechROs from Fitbit (rows) and PROs of Health-Related Quality of Life on the EQ-5D-3L scale (columns).

	PRO		Q1: Mobility			Q2: Self-Care			Q3: Usual Activities			Q4: Pain/Discomfort			Q5: Anxiety/Depression			Health Score			Mobility Numeric Score			Self-Care Numeric Score			Usual aCtivities Numeric Score			Pain/Discomfort Numeric Score			Anxiety/Depression Numeric Score		
Family	TechRO	Health	Both	Healthy	Diseased	Both	Healthy	Diseased	Both	Healthy	Diseased	Both	Healthy	Diseased	Both	Healthy	Diseased	Both	Healthy	Diseased	Both	Healthy	Diseased	Both	Healthy	Diseased	Both	Healthy	Diseased	Both	Healthy	Diseased	Both	Healthy	Diseased
Raw	Energy													$0.5_{-}$			$0.6_{-}$															$0.5_{-}$			$0.6_{-}$
	Steps		$0.4_{-}$	$0.4_{-}$	$0.5_{-}$												$0.5_{-}$				$0.4_{-}$	$0.4_{-}$	$0.5_{-}$												$0.5_{-}$
	Heart rate											$0.3_{-}$						$0.3_{-}$	$0.5_{-}$											$0.3_{-}$					
Processed	Sedentary		0.4	0.5								0.5	0.6		0.5	0.4	0.8	$0.3_{-}$	$0.3_{-}$		0.4	0.5								0.5	0.6		0.5	0.4	0.8
	Sedentary+light		0.3										0.5		0.5	0.4					0.3										0.5		0.5	0.4	
	Light		$0.3_{-}$	$0.3_{-}$										0.6	$0.4_{-}$	$0.4_{-}$			0.4		$0.3_{-}$	$0.3_{-}$										0.6	$0.4_{-}$	$0.4_{-}$	
	Light+fair																																		
	Fair											$0.3_{-}$	$0.5_{-}$	$0.6_{-}$		0.5		$0.5_{-}$	0.4											$0.3_{-}$	$0.5_{-}$	$0.6_{-}$		0.5	
	Fair+vigorous												$0.5_{-}$					$0.5_{-}$													$0.5_{-}$				
	Vigorous															0.4		$0.6_{-}$	$0.5_{-}$															0.4	
	Active		$0.3_{-}$													0.5	$0.6_{-}$	$0.7_{-}$			$0.3_{-}$													0.5	$0.6_{-}$
	Sleep											$0.5_{-}$			$0.5_{-}$	$0.4_{-}$	$0.5_{-}$	0.4												$0.5_{-}$			$0.5_{-}$	$0.4_{-}$	$0.5_{-}$
CLR PA	Sedentary		0.3										0.6		0.4	$0.5_{-}$		0.6			0.3										0.6		0.4	$0.5_{-}$	
	Light														$0.3_{-}$			0.4	0.4														$0.3_{-}$		
	Fair											$0.5_{-}$	$0.6_{-}$						$0.5_{-}$	$0.6_{-}$										$0.5_{-}$	$0.6_{-}$				
	Vigorous																	$0.3_{-}$		$0.6_{-}$															
CLR PA+S	Sedentary												0.6					0.6													0.6				
	Light															$0.4_{-}$			0.4															$0.4_{-}$	
	Fair											$0.6_{-}$						0.6	$0.6_{-}$											$0.6_{-}$					
	Vigorous											0.5						$0.3_{-}$	$0.6_{-}$											0.5					
	Sleep											$0.5_{-}$			$0.3_{-}$			0.5												$0.5_{-}$			$0.3_{-}$		
Raw	Total				1									1			2		1				1									1			2
Processed	Total			1								2	4	2	3	2	3	4	1			1								2	4	2	3	2	3
CLR PA	Total											1	2			1		1	1	2										1	2			1	
CLR PA+S	Total											3	1					3	2											3	1				
All Families	Total			1	1							6	7	3	3	3	5	8	5	2		1	1							6	7	3	3	3	5

Color coding: from orange (weaker correlation/fewer total correlations) to yellow to green (stronger correlation/more total correlations). Only significant correlations are shown. Only coefficients of 0.5 or above are highlighted.
